# Chlorinated Cubane-1,4-dicarboxylic Acids

**DOI:** 10.1021/acs.joc.2c02872

**Published:** 2023-02-01

**Authors:** Adéla Křížková, Guillaume Bastien, Igor Rončević, Ivana Císařová, Jiří Rybáček, Václav Kašička, Jiří Kaleta

**Affiliations:** † Institute of Organic Chemistry and Biochemistry of the Czech Academy of Sciences, Flemingovo nám. 2, 160 00 Prague 6, Czech Republic; ‡ Department of Inorganic Chemistry, Faculty of Science, Charles University in Prague, Hlavova 2030, 128 40 Prague 2, Czech Republic

## Abstract

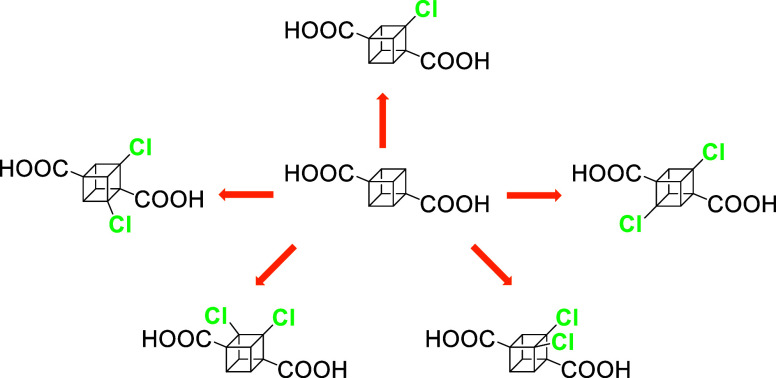

Herein, we report radical chlorination of cubane-1,4-dicarboxylic acid leading preferentially to one monochlorinated cubane dicarboxylate (ca. 70%) that is accompanied by four dichlorinated derivatives (ca. 20% in total). The exact positions of the chlorine atoms have been confirmed by X-ray diffraction of the corresponding single crystals. The acidity constants of all dicarboxylic acids in water were determined by capillary electrophoresis (3.17 ± 0.04 and 4.09 ± 0.05 for monochlorinated and ca. 2.71 ± 0.05 and 3.75 ± 0.05 for dichlorinated cubanes). All chlorinated derivatives as well as the parent diacid showed high thermal stability (decomposition above 250 °C) as documented by differential scanning calorimetry. The probable reaction pathways leading to individual isomers were proposed, and the energies of individual transition states and intermediates were obtained using density functional theory calculations (B3LYP-D3BJ/6-311+G(d,p)). The relative strain energies for all newly prepared derivatives as well as for hypothetical hexahalogenated (fluorinated, chlorinated, brominated, and iodinated) derivatives of cubane-1,4-dicarboxylic acids were predicted using wavefunction theory methods. The hexafluorinated derivative was identified as the most strained compound (57.5 kcal/mol), and the relative strain decreased as the size of halogen atoms increased (23.7 for hexachloro, 16.7 for hexabromo, and 4.0 kcal/mol for the hexaiodo derivative).

## Introduction

The extremely strained yet remarkably stable cubane system^1^ has been attracting unflagging scientific attention^2,3^ since its planful synthesis in 1964.^4−6^ Some of its potential applications include pharmaceuticals (a rigid cubane cage is considered a phenyl bioisoster),^7−9^ fuels,^6^ explosives,^10,11^ building blocks in material chemistry,^12,13^ etc. The recently published decagram synthesis^14^ of one of the key building blocks, dimethyl cubane-1,4-dicarboxylate, in combination with the rapid development of new chemical tools allowing the functionalization of its scaffold^15,16^ clearly indicates that the synthesis of cubane derivatives has not reached its end yet.

**Chart 1:**
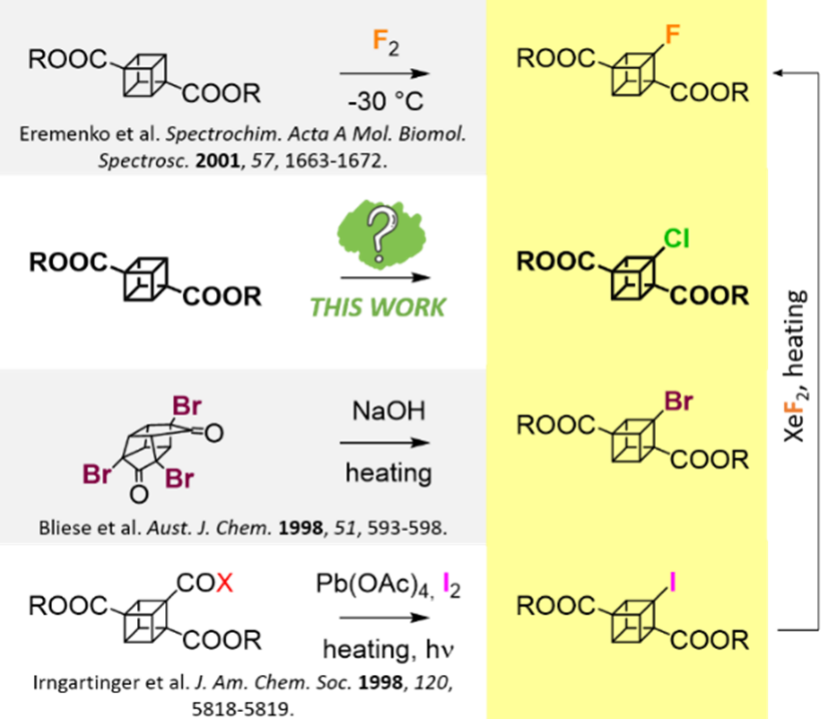
Known Routes toward Monofluorinated, Brominated, and Iodinated Derivatives of Cubane-1,4-dicarboxylic Acids

Halogenated cubane-1,4-dicarboxylic acids represent an interesting subclass of cubane derivatives. The presence of halogen atoms increases the volume of this unit, allowing for the fine tuning of its final geometric properties. This makes halogenated cubanes of potential interest for example in medicinal or supramolecular chemistry. Current synthetic methods offer tools for the construction of fluorinated,^17,18^ brominated,^19^ and iodinated^18^ cubane diacids/diesters. Somewhat surprisingly, the corresponding chlorinated analogues are unknown to the scientific community (Chart 1).

**Chart 2:**
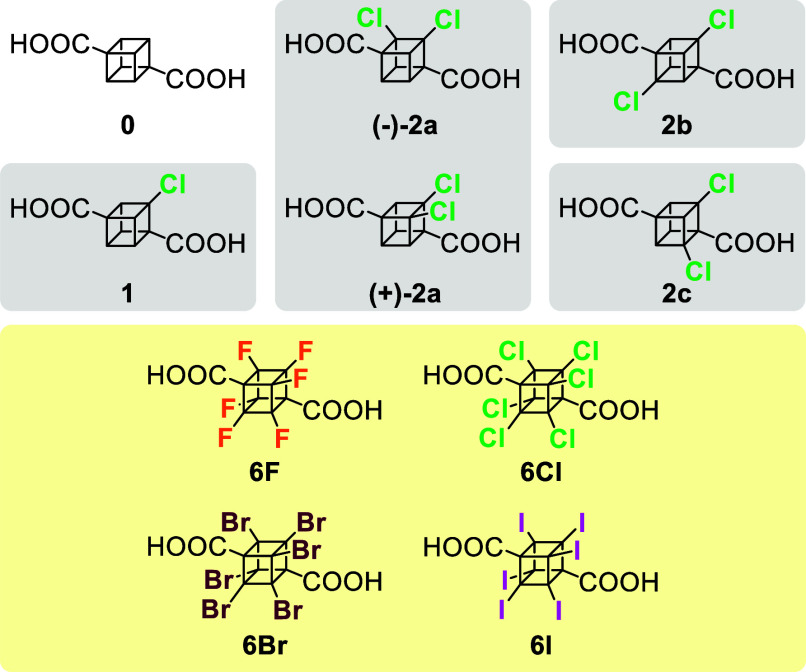
Structure of Cubane-1,4-dicarboxylic Acid (**0**), Newly Synthesized Chlorinated Derivatives **1**, **(−)-2a**, **(+)-2a**, **2b**, and **2c**, and Hypothetical Perhalogenated Diacids **6F**, **6Cl**, **6Br**, and **6I**

We decided to fill this gap by taking inspiration from the direct halogenations of parent cubane^20,21^ and utilizing our extensive experience with chlorination reactions of strained bicyclo[1.1.1]pentane cages.^22,23^ Our motivation was a desire to prepare and determine structural and physical properties of these unknown structures in order to use them as unique building blocks in our ongoing research focused on the study of (dipolar) rotors in the solid state.^24−27^ Herein, we report the synthesis of mono- **1** and all four possible dichlorinated cubane-1,4-dicarboxylates (**(+)-2a**, **(−)-2a**, **2b**, and **2c**), the electronic circular dichroism (ECD) spectra and optical rotation of both enantiomers of **2a**, the crystal structures of these five compounds, the relative strain energies of newly synthesized structures as well as of hypothetical hexahalogenated diacids (**6F**, **6Cl**, **6Br**, and **6I**), and acidity constants for the newly prepared diacids (Chart 2). The formation of individual dichlorinated isomers was rationalized by density functional theory (DFT) calculations.

The Arabic numbers indicate the number of halogen atoms in the molecule, the suffixes “**a**–**c**” represent individual isomers, and “Me” stands for the corresponding dimethyl ester.

## Results and Discussion

**Scheme 1:**
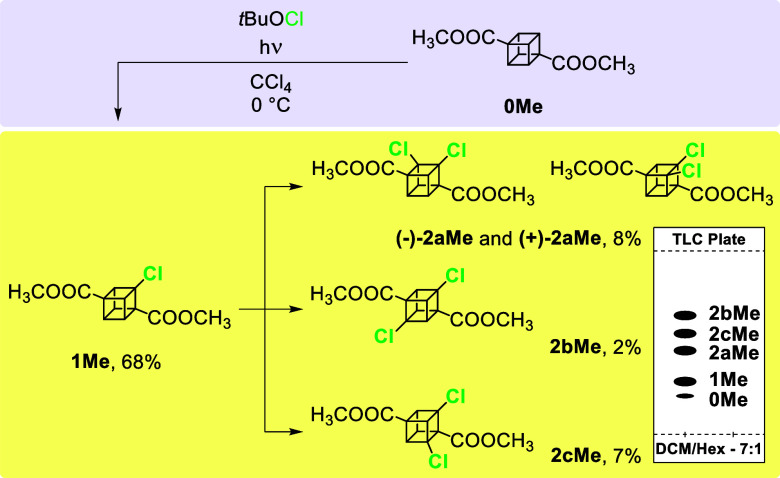
One-Pot Radical Chlorination of **0Me** Resulting in a Mixture of **1Me** and Products of Its Subsequent Halogenation (**2aMe**, **2bMe**, and **2cMe**) with the Yellow Box Representing the Composition of the Reaction Mixture

The UV-light initiated radical chlorination of **0Me** using *t-*BuOCl in CCl_4_ (this protocol^28^ was already applied to various strained polycyclic hydrocarbons^29^ including parent cubane^20^) at 0 °C under optimized reaction conditions resulted in the preferential formation of monochlorinated **1Me** (68%) that was accompanied by a mixture of dichlorinated cages **2aMe**–**2cMe** in an approximate 4:1:3.5 ratio (Scheme 1). The **2aMe**–**2cMe** were products of the subsequent halogenation of **1Me**, and their concentration did not exceed 10% each by following the given protocol. Individual compounds were easily separated using silica gel column chromatography. All attempts to completely convert **1Me** into **2aMe**–**2cMe** as well as to force a higher degree of chlorination failed. We speculate that the further chlorination of already dichlorinated structures leads to the destruction of the strained cubane cage judging based on the complex ^1^H NMR spectra of the crude reaction mixtures (containing among others vinylic-like resonances suggesting cage opening).

Performing this reaction at higher temperatures (above 10 °C) resulted in fast formation of a significant amount of undesired (most likely rearranged) side-products that complicated separation of expected chlorinated cages. Substitution of relatively toxic CCl_4_ by CHCl_3_ or 1,2-dichloroethane was usually accompanied by notably lower yields. Direct light-initiated chlorination using saturated solution of Cl_2_ in CCl_4_ even at low temperature caused degradation of the starting material. The same observation was made also for the parent cubane.^20^

**Scheme 2:**
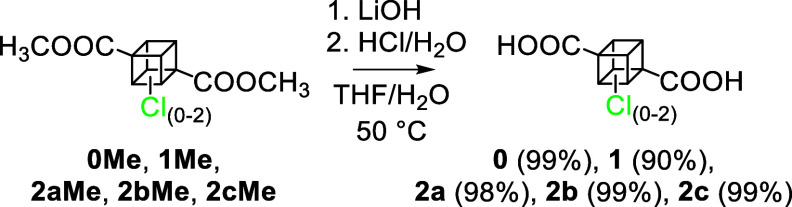
Basic Hydrolysis of Dimethyl Esters to the Corresponding Diacids

All five esters were hydrolyzed to corresponding diacids, and products were isolated in high (90–99%) yields (Scheme 2). It is worth mentioning that in comparison to parent **0**, the chlorination markedly enhanced the solubility of corresponding diacids in common organic solvents.

**Figure 1:**
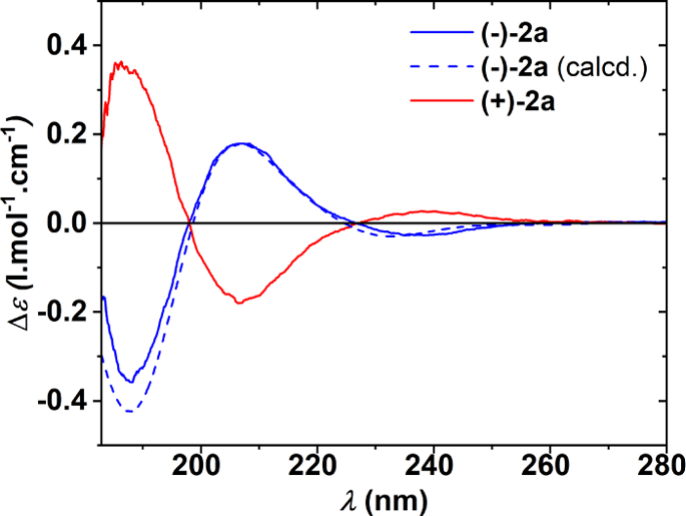
Experimental ECD spectra of **(−)-2a** (solid blue line) and **(+)-2a** (solid red line) together with the calculated spectrum of **(−)-2a** (dashed blue line). The spectra were recorded in the 2:1 mixture of 2,2,2-trifluoroethanol and methanol.

The chlorination afforded **2aMe** as a racemic mixture of two enantiomers **(+)-2aMe** and **(−)-2aMe**. We succeeded to separate them in the form of corresponding diacids using high-performance liquid chromatography (HPLC) on a chiral column (Figure S3). The first eluted enantiomer, **(−)-2a** ([α]^20^_589_ = −4.7), was optically pure (>99% *ee*), whereas the second species, **(+)-2a** ([α]^20^_589_ = +5.4), was enantioenriched to ca. 93% *ee* (Figure S4). Determination of the absolute configuration of compounds **(+)-2a** and **(−)-2a** was based on good agreement between the ECD spectrum obtained experimentally for the HPLC-resolved (−)-**2a** enantiomer and the spectrum simulated by TD-DFT for the corresponding enantiomer (Figure 1).

**Figure 2:**
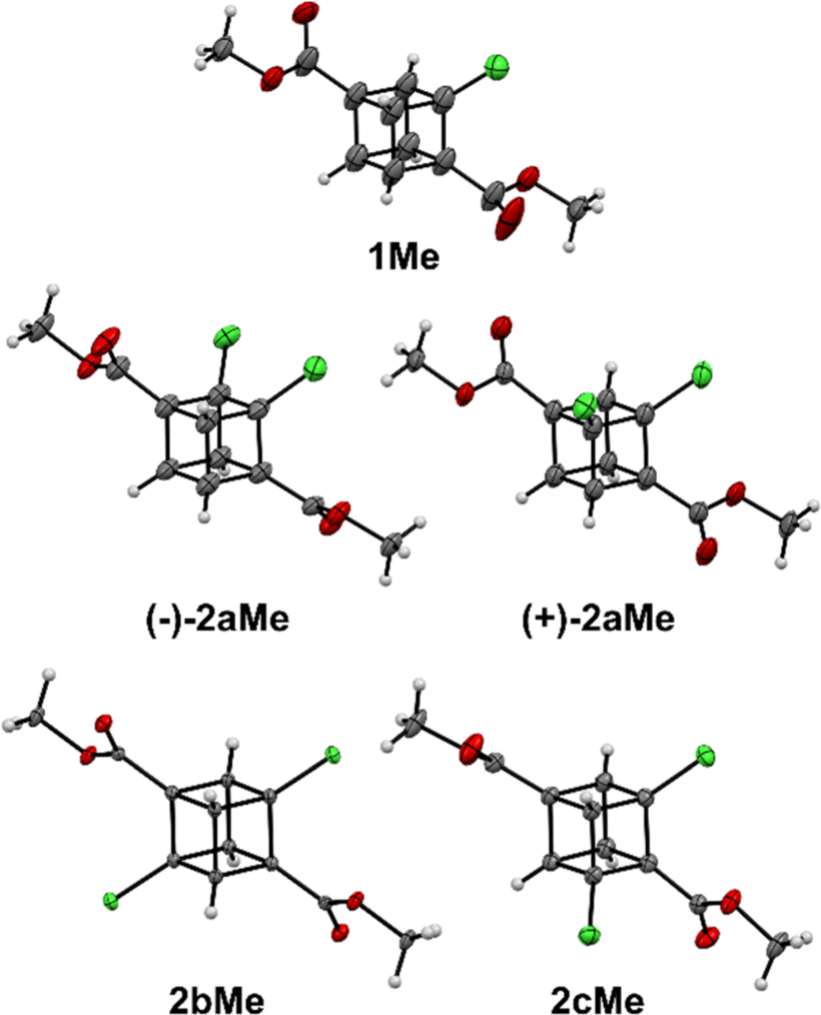
ORTEP representation of cubane derivatives **1Me**, **(−)-2aMe**, **(+)-2aMe**, **2bMe**, and **2cMe**. Thermal ellipsoids are shown with 30% probability. Atom labeling: hydrogen = white, carbon = gray, oxygen = red, and chlorine = green.

The single crystals of all synthesized chlorinated cubanes were successfully prepared, and subsequent X-ray analysis helped to unequivocally assign the position of chlorine atoms within individual derivatives (Figure 2). Compounds **(−)-2aMe** and **(+)-2aMe** cocrystallized like a racemic mixture.

The presence of an electronegative element on the cubane cage affects the acidity of carboxylic functions, which prompted us to determine the acidity constants (p*K*_a_) of parent **0** and all chlorinated systems using capillary electrophoresis (Table 1 and Table S3). The comparison of individual p*K*_a_ values allows us to draw the following two conclusions: (i) the attachment of the first and second chlorine atom reduces p*K*_a,1_ by ∼0.5 and p*K*_a,2_ by ∼0.4 and (ii) the mutual position of chlorine atoms within **2a**–**2c** has a nearly zero effect on the p*K*_a_ values.

**Table 1:** Experimentally Determined Acidity Constants (p*K*_a_) of **0**, **1**, **2a**, **2b**, and **2c**^a^

compd.	p*K*_a,1_th	p*K*_a,2_th
**0**	3.63 ± 0.03	4.76 ± 0.04
**1**	3.17 ± 0.04	4.09 ± 0.05
**2a**	2.70 ± 0.04	3.73 ± 0.05
**2b**	2.73 ± 0.04	3.68 ± 0.05
**2c**	2.71 ± 0.03	3.83 ± 0.04

a p*K*_a_^th^, the thermodynamic acidity constant (at zero ionic strength). All values are related to the temperature of 25 °C.

Differential scanning calorimetry (DSC) revealed that all analyzed diacids do not melt, and their decomposition is characterized by exotherms at 248–274 °C. The diacids **0**, **1**, **2a**, and **2c** showed slightly lower thermal stability (decomposition at 248–257 °C) compared to **2b**, which decomposed at 274 °C (Figure S1A in the Supporting Information). All five methyl esters melted as documented by sharp endotherms, and the melting points of chlorinated esters were noticeably lower (119 °C for **1Me**, 123 °C for **2aMe**, and 151 °C for **2cMe**) compared to the starting **0Me** (160 °C). The only exception was **2bMe** that melted at 214 °C. All esters decomposed in the relatively narrow region of 257–263 °C, as documented by characteristic exotherms (Figure S1B in the Supporting Information).

**Scheme 3:**
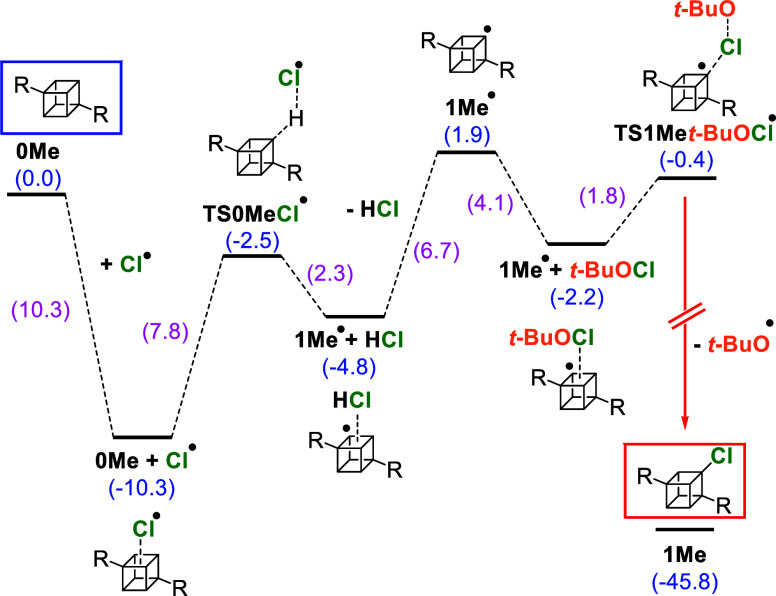
Radical Chlorination of **0Me** (Framed in Blue) and the Subsequent Formation of **1Me** (Framed in Red) Relative energies including ZPVE (in blue) as well as differences between two neighboring states (in purple) are in kcal/mol. For clarity, the ester group (COOCH_3_) has been replaced with “R”.

**Scheme 4:**
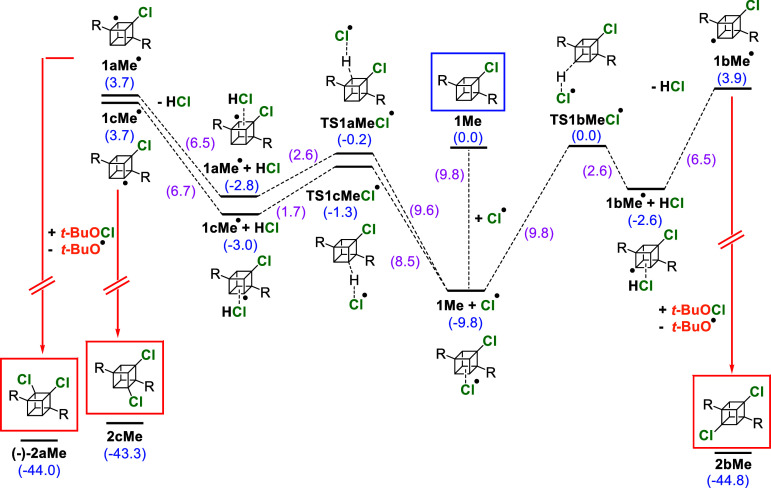
Radical Chlorination of **1Me** (Framed in Blue), Leading to **2aMe** (only the **(−)-2aMe** Enantiomer Is Depicted for Clarity), **2bMe,** and **2cMe** (Framed in Red) Relative energies including ZPVE (in blue) as well as differences between two neighboring states (in purple) are in kcal/mol. For clarity, the ester group (COOCH_3_) has been replaced with “R”.

The proposed mechanisms of radical chlorinations were explored computationally using DFT, with results shown in Schemes 3 and 4. The radical chlorination of **0Me** (Scheme 3) starts with the formation of a relatively stable (−10.3 kcal/mol) complex between **0Me** and a chlorine radical (Cl^•^). The chlorine atom then abstracts a hydrogen atom from the cage (*E*_TS_ = −2.5 kcal/mol), which results in the formation of HCl and the **1Me^•^** radical (1.9 kcal/mol). The abstraction of a hydrogen atom from **0Me** by *t*-BuO^**•**^ was also considered (Scheme S1 in the Supporting Information), but it was found to have a higher barrier (2.4 kcal/mol). The formed **1Me^•^** radical then reacts with *t*-BuOCl (*E*_TS_ = −0.4 kcal/mol) to irreversibly form **1Me** and *t*-BuO^**•**^ (−45.8 kcal/mol).

The addition of the second chlorine to **1Me** can lead to **2aMe**, **2bMe**, or **2cMe** (Scheme 4). DFT predicts the formation of a **1Me** + Cl^•^ intermediate (−9.8 kcal/mol relative to **1Me**). From this, the hydrogen atom transfer can occur in three ways: by forming a **1aMe**^•^ radical (*E*_TS_ = −0.2 kcal/mol), a **1bMe**^•^ radical (*E*_TS_ = 0.0 kcal/mol), or a **1cMe**^•^ radical (*E*_TS_ = −1.3 kcal/mol). These radicals then abstract a chlorine atom from *t*-BuOCl in a manner similar to **1Me**^•^ to form the corresponding dichlorinated cubanes **2aMe**, **2bMe**, or **2cMe**. These results are in reasonable agreement with experiments in which the kinetically and thermodynamically favored **2aMe** and **2cMe** are formed in larger amounts than **2bMe**.

**Scheme 5:**
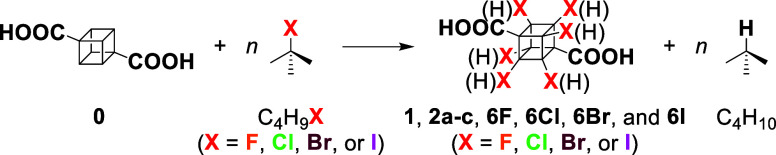
Hyperhomodesmotic Reaction Scheme Used for the Calculation of Relative Strain Energies

The strain energies relative to **0** were calculated at the DLPNO-CCSD(T)/cc-PVQZ level of theory on geometries obtained using both DLPNO-SCS-MP2/cc-PVQZ (Table 2) and B3LYP/6-311+G(d,p) (Table S1 in the Supporting Information). A hyperhomodesmotic^30^ reaction scheme preserves the number and hybridization of all carbon atoms (Scheme 5). Similar reaction schemes had been used for the cubanes^31,32^ as well as for the bicyclo[1.1.1]pentanes.^22,23^

**Table 2:**
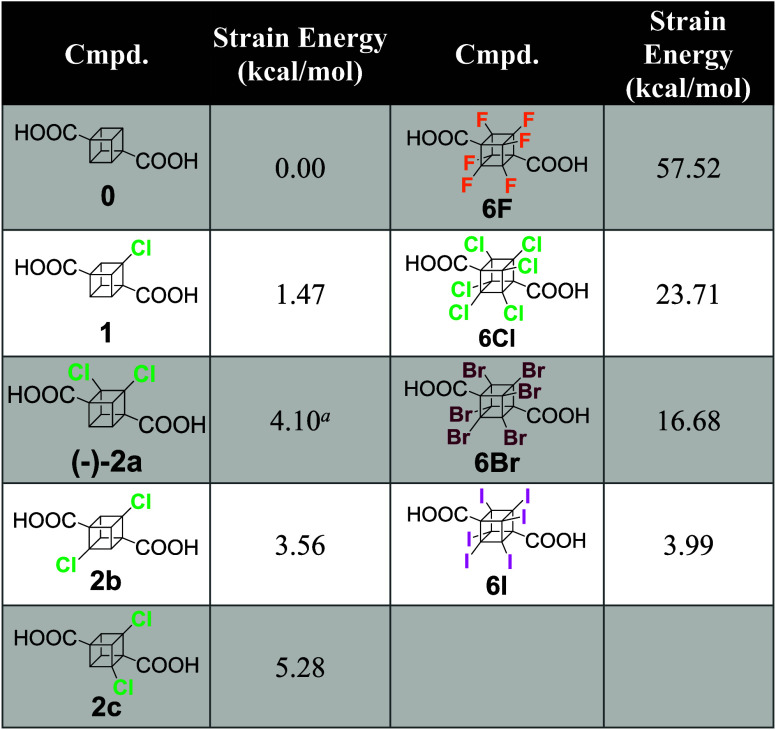
Calculated Relative Strain Energies (kcal/mol) in Cubane-1,4-dicarboxylic Acids at the DLPNO-CCSD(T)/cc-PVQZ Level of Theory, Obtained Using Geometries Optimized at DLPNO-SCS-MP2/cc-PVQZ^a^

a Only one enantiomer is depicted for clarity.

The calculated relative strain energies (Table 2) show that the chlorination of **0** is associated with a small (1.47 kcal/mol) increase in the strain. Introducing a second chlorine (**2a**–**c**) more than doubles the strain, with **2c** being significantly more strained (5.28 kcal/mol) than **2b** (3.56 kcal/mol) and **2a** (4.10 kcal/mol). This is likely due to the fact that the carboxylic group is flanked by two chlorines which introduce repulsive Cl•••O interactions and which are not present in **2a** or **2b**.

The expected trend in the increase of the relative strain energy as a function of attached chlorine substituents prompted us to computationally explore also the perhalogenated diacids that were intuitively expected to be the most strained members of individual series. As a side note, the hexafluorinated cubane **6F** was recently synthesized (in the form of corresponding diester),^33^ while **6Cl**, **6Br**, and **6I** are hypothetical structures so far. The calculations reveal a very large amount of strain in **6F** (57.52 kcal/mol) which rapidly decreases as we go down the group (23.71 kcal/mol in **6Cl**). The mild strain in **6Br** (13.65 kcal/mol) and the very small strain in **6I** (3.99 kcal/mol) are especially interesting and cannot be reproduced by dispersion-corrected B3LYP (which gives results within 0.5 kcal/mol for **0**-**2c**, and within 1.5 kcal/mol for **6F** and **6Cl**; see Table S1 in the Supporting Information). The cause for the loss of strain in the **6F**-**6I** series is a combination of two factors: (i) the decrease in the electrostatic repulsion between the halogens resulting from the decrease in electronegativity and increase in the X•••X distance, and (ii) the onset of attractive halogen-halogen interactions which are strongest in the case of iodine pairs.^34^ These interactions are also the cause of the stabilization in **6I**, where the closest X•••X distance is 3.9 Å which is very close to the ideal value of about 4.0 Å.^34^

In conclusion, we have developed a synthetic procedure which allows for a smooth chlorination of **0Me** that predominantly provides **1Me**, accompanied by small amounts of **2aMe**–**2cMe**. The reaction pathways as well as the preferential formations of certain products were explored using DFT calculations. The structures of all cubane derivatives were confirmed by X-ray diffraction. Acidity constants p*K*_a_ of corresponding diacids were determined by capillary electrophoresis, and stability was explored by DSC.

## Experimental Part

### Materials

All reactions were carried out under a nitrogen atmosphere with dry solvents freshly distilled under anhydrous conditions. Yields refer to isolated, chromatographically, and spectroscopically homogeneous materials.

Dimethyl cubane-1,4-dicarboxylate (**0Me**) was prepared according to the already published procedure.^6^ All other reagents were used as supplied unless otherwise stated.

The lamp used for the photochemical reactions is a 450 W mercury lamp from the company Hanovia with no filter. The reaction mixtures were kept in a quartz tube (external diameter 2 cm) and irradiated from 5 cm.

### X-ray Diffraction

Suitable crystals of all diesters were obtained by slow diffusion of pentane into corresponding dichloromethane solutions.

The crystallographic data for samples **1Me**, **2aMe**, **2bMe**, and **2cMe** were obtained from measurement on a Bruker D8 VENTURE Kappa Duo diffractometer with a PHOTON III detector, using an X-ray source IμS micro-focus sealed tube either CuKα (λ = 1.54178) radiation (**2aMe**, **2cMe**) or MoKα (0.71073 Å) radiation (**1Me**, **2bMe**). All crystals were cooled at a temperature of 120(2)K during the experiment. The structures were solved by direct methods (XT)^35^ and refined by full matrix least-squares based on *F*^2^ (SHELXL2018).^36^

The precision of results of **1Me** is hampered by disorder in the position of the Cl atom as well as one oxygen of the carboxyl moiety. On the other hand, the crystals with two Cl atoms on the cubane moiety are well ordered.

The hydrogen atoms on carbon atoms were fixed into idealized positions (riding model) and assigned temperature factors, either H_iso_(H) = 1.2 U_eq_(pivot atom) or H_iso_(H) = 1.5 U_eq_ (pivot atom) for the methyl moiety.

### High-Performance Liquid Chromatography (HPLC)

Enantiomers of **2a** were separated on an Interchim puriFlash 5.250 system using a CHIRALPAK IE (5 μm, 20 mm ID × 250 mm) column and 5% isopropyl alcohol in *n*-heptane (0.1% TFA) as the mobile phase at a flow rate of 20 mL/min.

### Ultrahigh-Performance Supercritical Fluid Chromatography (UHPSFC)

Enantiomeric excess (*ee*) was determined via supercritical fluid chromatography (SFC) using a UHPSFC/MS system (Waters UPC^2^ with DAD and QDa detectors). LC–MS grade isopropyl alcohol in CO_2_ (99.995%) 8:92 was used as the mobile phase (total flow rate = 1.5 mL/min, temperature = 35 °C, backpressure = 2000 psi, column = Chiralpak IE-3 (3 μm, 3 mm ID × 150 mm, DAICEL)). Chromatograms were collected by the ESI-MS detector in negative mode with selective ions recording at *m/z* = 259 using a make-up solvent of 10 mM aqueous formic acid – LCMS grade methanol 10:90.

### Electronic Circular Dichroism

The ECD spectra were measured on a Jasco-815. The ECD and absorption spectra were measured over a spectral range of 180 nm to 300 nm in solution of the mixture of 2,2,2-trifluoroethanol and methanol in 2:1 ratio and 8.9 × 10^–3^ M concentration. The measurements were made in a cylindrical quartz cell with a 0.2 mm path length using a scanning speed of 10 nm/min, a response time of 8 s, and standard instrument sensitivity. The final spectrum was obtained as result of three scans. After a baseline correction, spectra were expressed in terms of differential molar extinction (Δε) and molar extinction (ε), respectively.

### Optical Rotation (OR)

The OR was obtained using AUTOPOL IV (Rudolph Research Analytical, USA) in the mixture of 2,2,2-trifluoroethanol and methanol in the 2:1 ratio and 8.9 × 10^–3^ M concentration (same as used for ECD).

### Differential Scanning Calorimetry

DSC traces were recorded on a DSC 250 apparatus from TA instrument company. All samples were analyzed from +25 to +300 °C then back to +25 °C with a ramp of 10 °C/min except compound **0Me** which was heated only up to 280 °C.

### Capillary Electrophoresis (CE)

CE experiments were performed on a CE analyzer P/ACE^TM^ MDQ DNA System (Beckman-Coulter, Fullerton, CA, USA) equipped with a UV–vis spectrophotometric diode array detector set at 195, 200, and 210 nm wavelengths. A bare fused silica capillary with an outer polyimide coating (id/od 50/375 μm, total/effective (to the detector) length 398/297 mm was provided by Polymicro Technologies (Phoenix, AZ, USA).

### Calculations

All geometries were optimized using the B3LYP-D3BJ/6-311+G(d,p) method^37,38^ which we have found to produce good results for similar cage compounds.^22,23,39^ SDD pseudopotentials were used for core electrons of Br and I.^40^ Harmonic frequency calculations were run on all structures, in order to confirm that minima have zero imaginary vibrational frequencies, and that transition structures have exactly one. All reaction paths include zero-point vibrational energies and solvation energy (calculated using the SMD model^41^), making them fully comparable with our previous work on bicyclo[1.1.1]pentanes.^22,23^ These calculations were done using Gaussian16.^42^

Strain energies were calculated using the DLPNO-CCSD(T) method^43^ (domain-based local pair natural orbital coupled cluster singles doubles perturbative triples) and the cc-PVQZ basis set (and SK-MCDHF-RSC pseudopotentials for Br and I).^44,45^ As large discrepancies between B3LYP and coupled cluster strain energies were found for **6Br** and **6I**, geometries were reoptimized using DLPNO-SCS-MP2^46^ (domain-based local pair natural orbital spin component-scaled second order perturbation theory), which gave strain energies more similar to coupled clusters. Convergence with respect to a complete basis was checked by performing DLPNO-CCSD(T)/cc-PVTZ calculations with F12 corrections,^47^ which revealed errors around 0.2 kcal/mol due to basis set incompleteness (Table S2). Coupled clusters and MP2 calculations were done using ORCA.^48^

ECD Spectrum Calculations: Gaussian 16^42^ software package was used to optimize the geometry of (*−*)-**2a** at the CAM-B3LYP^49^/cc-pVTZ^44^/GD3^50^/PCM^51,52^ (2,2,2-trifluoroethanol) level of theory with subsequent frequency analysis, which confirmed the absence of any negative vibrational frequencies. Finally, ECD transitions were extracted from TD-DFT calculation performed at the same level of theory for 150 lowest-energy singlet states and the corresponding spectrum constructed by application of Gaussian broadening and red-shifted by 45 nm.

### Procedures

Analytical thin-layer chromatography (TLC) was performed using precoated TLC aluminum sheets (Silica gel 60 F_254_). TLC spots were visualized using either UV light (254 nm) or a 5% solution of phosphomolybdic acid in ethanol, and heat (400 *°*C) as a developing agent. Flash chromatography was performed using silica gel (high purity grade, pore size 60 Å, 70–230 mesh). Melting points are reported uncorrected. Chemical shifts in ^1^H and ^13^C spectra are reported in ppm on the δ scale relative to CHCl_3_ (δ = 7.26 ppm for ^1^H and δ = 77.2 ppm for ^13^C), acetone-*d*_6_ (δ = 2.05 ppm for ^1^H, and δ = 29.8 ppm for ^13^C), DMSO-*d*_6_ (δ = 2.50 ppm for ^1^H NMR and δ = 39.5 ppm for ^13^C NMR) as internal references. Structural assignments were made with additional information from gCOSY, gHSQC, and gHMBC experiments. The volume/volume (v/v) ratios of solvents were used to prepare mobile phases for column chromatography.

High-resolution mass spectra (HRMS) using electrospray ionization (ESI) were obtained on a mass analyzer combining linear ion trap and the Orbitrap, and those using electron impact ionization (EI) mode were taken on a time-of-flight mass spectrometer.

The chlorine substituted compounds show a characteristic ^35^Cl/^37^Cl pattern in the mass spectra. Only the most intense peaks from these complex isotopic patterns are reported in the case of dichlorinated derivatives.

### Dimethyl Cubane-1,4-dicarboxylate (**0Me**)^6^

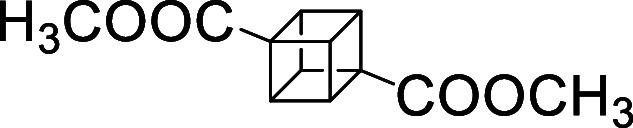



Diacid **0** (2.00 g, 10.41 mmol) was dissolved in methanol (50 mL), and SOCl_2_ (5 mL) was added dropwise at room temperature for 5 min. The colorless reaction mixture was stirred at room temperature for additional 16 h. The solvent was removed under reduced pressure yielding **0Me** as a white crystalline solid (2.29 g, 10.40 mmol, 100%) that usually did not require additional purification.

Mp 162.1–164.3 °C (DSC: 160.3 °C). ^1^H NMR (400 MHz, CDCl_3_): δ 4.22 (s, 6H), 3.70 (s, 6H). ^13^C {^1^H} NMR (100 MHz, CDCl_3_): δ 172.1, 55.9, 51.7, 47.2. IR (KBr): 3003, 2955, 2852, 1722, 1697, 1441, 1370, 1326, 1220, 1200, 1093, 1031, 967, 913, 845, 836, 824, 790, 732, 614, 431 cm^–1^. MS, *m/z* (%): 221.1 (85, M + H), 189.1 (100, M – 2 × COOCH_3_ + H). HRMS (APCI) *m/z*: [M + H]^+^ calcd for C_12_H_13_O_4_^+^ 221.0808; found 221.0809. Anal. calcd for C_12_H_12_O_4_: C, 64.45; H, 5.49. Found: C, 64.60; H, 5.36.

### Radical Chlorination of **0Me**

Freshly synthesized *t*-BuOCl^21^ (10 mL, 87.5 mmol) was added into a solution of **0Me** (1.20 g, 5.45 mmol) in dry CCl_4_ (9 mL), and the clear yellow reaction mixture was irradiated for 30 min at 0 °C using a 450 W mercury lamp. Progress of the reaction was monitored by ^1^H NMR (the reaction usually stopped spontaneously in less than 30 min due to rapid decomposition of *t*-BuOCl). In the case that starting **0Me** was still present, a second portion of *t*-BuOCl (10 mL, 87.5 mmol) was injected, and irradiation was prolonged for additional 30 min at 0 °C. Subsequently, solvents were removed under reduced pressure, and the yellowish oily residue was chromatographed on silica gel (CH_2_Cl_2_/hexane – 7:1) yielding two fractions: the first one containing a mixture of dichlorinated cubanes **2aMe**–**2cMe** and the second fraction containing **1Me** as an off-white solid (940 mg, 3.69 mmol, 68%). If **1Me** appears to be contaminated by traces of **0Me**, a second column chromatography on silica gel (CH_2_Cl_2_) delivered clean **1Me**. The dichlorinated cubanes **2aMe**–**2cMe** were separated also by column chromatography on silica gel (hexane/ethyl acetate – 10:1) eluting first **2bMe** (25 mg, 0.09 mmol, 2%), followed by **2cMe** (105 mg, 0.36 mmol, 7%), and finally **2aMe** (120 mg, 0.42 mmol, 8%) as white crystalline solids.

### Dimethyl 2-Chlorocubane-1,4-dicarboxylate (**1Me**)

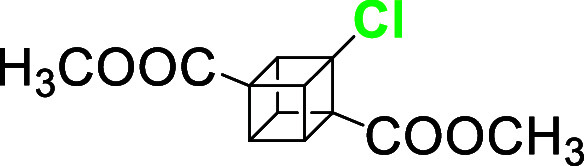



Mp 119.9–122.8 °C (DSC: 118.8 °C). ^1^H NMR (400 MHz, CDCl_3_): δ 4.41–4.37 (m, 2H), 4.27–4.19 (m, 3H), 3.76 (s, 3H), 3.72 (s, 3H). ^13^C {^1^H} NMR (100 MHz, CDCl_3_): δ 170.5, 168.5, 69.6, 62.9, 56.3, 52.9, 52.1, 52.0, 48.1, 44.1. IR (KBr): 3009, 2959, 2850, 1718, 1460, 1438, 1330, 1221, 1158, 1107, 1092, 980, 906, 845, 793 cm^–1^. MS, *m/z* (%): 257.0 (20, M), 255.0 (100, M), 236.0 (25), 223.1 (45, M-2×CH_3_), 219.1 (20, M – Cl), 197.0 (15, M-COOCH_3_), 195.0 (70, M-COOCH_3_). HRMS (APCI) *m/z*: [M + H]^+^ calcd for C_12_H_12_ClO_4_^+^ 255.0419; found 255.0417. Anal. calcd for C_12_H_11_ClO_4_: C, 56.60; H, 4.35. Found: C, 56.94; H, 4.46.

### Dimethyl (1*S*,2*R*,3*R*,4*S*,5*R*,6*R*,7*R*,8*R*)-2,3-Dichlorocubane-1,4-dicarboxylate (**(−)-2aMe**) and Dimethyl (1*R*,2*S*,3*S*,4*R*,5*S*,6*S*,7*S*,8*S*)-2,3-Dichlorocubane-1,4-dicarboxylate (**(+)-2aMe**)

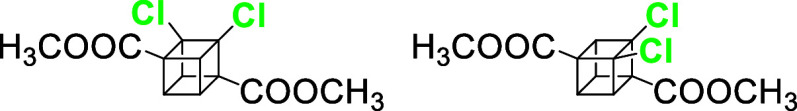



Mp 125.2–126.2 °C (DSC: 123.1 °C). ^1^H NMR (400 MHz, CDCl_3_): δ 4.43–4.41 (m, 2H), 4.26–4.24 (m, 2H), 3.78 (s, 6H). ^13^C {^1^H} NMR (100 MHz, CDCl_3_): δ 167.4, 74.3, 60.6, 53.8, 52.3, 45.1. IR (KBr): 3017, 2958, 2853, 1725, 1439, 1370, 1335, 1218, 1177, 1161, 1151, 1103, 1029, 1007, 989, 950, 920, 861, 810, 776, 700, 634, 440, 427 cm^–1^. MS, *m/z* (%): 291.0 (18, M + H), 289.0 (30, M + H), 253.0 (45, M-Cl), 231.0 (12, M-COOCH_3_), 229.0 (30, M-COOCH_3_), 172.0 (65, M – 2 × COOCH_3_), 170.0 (100, M – 2 × COOCH_3_). HRMS (APCI) *m/z*: [M– H]^+^ calcd for C_12_H_11_O_4_Cl_2_^+^ 289.0029; found 289.0029. Anal. calcd for C_12_H_10_Cl_2_O_4_: C, 49.85; H, 3.49. Found: C, 49.60; H, 3.47.

### Dimethyl 2,5-Dichlorocubane-1,4-dicarboxylate (**2bMe**)

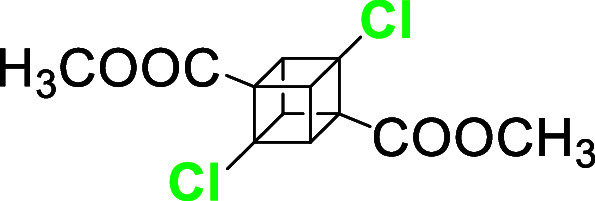



Mp 215.8–216.5 °C (DSC: 213.9 °C). ^1^H NMR (400 MHz, CDCl_3_): δ 4.40 (s, 4H), 3.79 (s, 6H). ^13^C {^1^H} NMR (100 MHz, CDCl_3_): δ 167.2, 70.3, 52.9, 52.4. IR (KBr): 3020, 2961, 2853, 1730, 1440, 1370, 1335, 1223, 1206, 1191, 1128, 1104, 1022, 997, 977, 912, 841, 804, 685, 640, 480, 435 cm^–1^. MS, *m/z* (%): 291.0 (45, M + H), 289.0 (70, M + H), 257.0 (15), 253.0 (100, M – Cl), 231.0 (15, M-COOCH_3_), 229.0 (55, M-COOCH_3_), 221.0 (15). HRMS (APCI) *m/z*: [M – H]^+^ calcd for C_12_H_11_O_4_Cl_2_^+^ 289.0029; found 289.0033. Anal. calcd for C_12_H_10_Cl_2_O_4_: C, 49.85; H, 3.49. Found: C, 50.11; H, 3.50.

### Dimethyl 2,6-Dichlorocubane-1,4-dicarboxylate (**2cMe**)

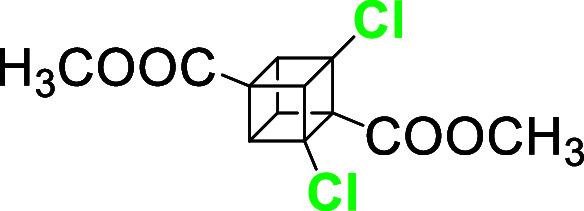



Mp 149.2–152.9 °C (DSC: 150.6 °C). ^1^H NMR (400 MHz, CDCl_3_): δ 4.56–4.54 (m, 1H), 4.39–4.37 (m, 2H), 4.29–4.26 (m, 1H), 3.82 (s, 3H), 3.76 (s, 3H). ^13^C {^1^H} NMR (100 MHz, CDCl_3_): δ 169.2, 165.5, 68.9, 66.2, 64.3, 57.0, 52.4, 52.3, 49.3, 40.5. IR (KBr): 3015, 2954, 2850, 1727, 1460, 1440, 1370, 1332, 1218, 1156, 1130, 1109, 1092, 1080, 1020, 998, 978, 959, 933, 902, 856, 845, 811, 795, 749, 438, 407 cm^–1^. MS, *m/z* (%): 291.0 (45, M + H), 289.0 (75, M + H), 259.0 (20), 257.0 (35), 253.0 (100, M-Cl). HRMS (APCI) *m/z*: [M + H]^+^ calcd for C_12_H_11_O_4_Cl_2_^+^ 289.0029; found 289.0026. Anal. calcd for C_12_H_10_Cl_2_O_4_: C, 49.85; H, 3.49. Found: C, 49.61; H, 3.38.

### General Procedure for Basic Hydrolysis (GP1)

To a solution of diester (1 eq) in the mixture of THF and water in 2:1 ratio was added LiOH·H_2_O (20 eq) The mixture was stirred and heated at 50 °C in an oil bath for 16 h. Then, solvents were removed under reduced pressure. The crude product was dissolved in water (20 mL) and extracted with Et_2_O (1 × 50 mL). The aqueous phase was acidified to pH ∼ 1 by addition of concentrated HCl. The water phase was then extracted with Et_2_O (5 × 30 mL). The combined organic phases were dried over MgSO_4_, filtered, and concentrated under reduced pressure. The solid residue was triturated and centrifuged (3000 rpm for 3 min) with CH_2_Cl_2_ (1 × 5 mL) followed by pentane (2 × 10 mL). The supernatant was removed after every trituration cycle. The solid residue was thoroughly dried using Kugelrohr distillation apparatus (30 min, 80 °C, 600 mTorr) delivering desired diacids as white solids.

### Cubane-1,4-dicarboxylic Acid (**0**)

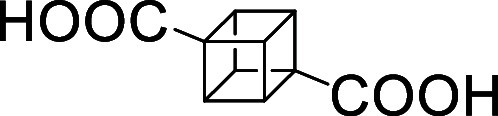



The title product was synthesized from **0Me** (500 mg, 2.27 mmol) and LiOH·H_2_O (1.91 g, 45.41 mmol) in the mixture of THF (20 mL) and H_2_O (10 mL) according to **GP1**. Diacid **0** was obtained as a white crystalline solid (430 mg, 2.24 mmol, 99%).

Mp >250 °C (dec.). ^1^H NMR (400 MHz, DMSO-*d*_6_): δ 12.35 (s, 2H), 4.11 (s, 6H). ^13^C {^1^H} NMR (100 MHz, DMSO-*d*_6_): δ 172.4, 55.5, 46.0. IR (KBr): 3436, 3002, 2828, 2675, 2564, 2532, 1691, 1628, 1435, 1367, 1296, 1254, 1242, 1207, 1107, 1100, 1014, 934, 883, 855, 756, 732, 608, 467, 436 cm^–1^. MS, *m/z* (%): 191.0 (100, M – H). HRMS (ESI−) *m/z*: [M – H]^−^ calcd for C_10_H_7_O_4_^–^ 191.0350; found 191.0349. Anal. calcd for C_10_H_8_O_4_: C, 62.50; H, 4.20. Found: C, 61.85; H, 4.13.

### 2-Chlorocubane-1,4-dicarboxylic Acid (**1**)

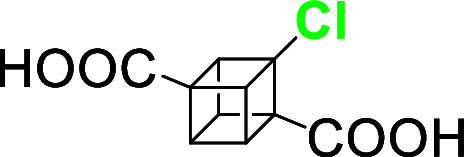



The title compound was synthesized from **1Me** (150 mg, 0.589 mmol) and LiOH·H_2_O (495 mg, 11.78 mmol) in the mixture of THF (10 mL) and H_2_O (5 mL) according to **GP1**. Diacid **1** was obtained as a white crystalline solid (120 mg, 0.530 mmol, 90%).

Mp >250 °C (dec.). ^1^H NMR (400 MHz, acetone-*d*_6_): δ 4.39–4.35 (m, 2H), 4.26–4.20 (m, 6H). ^13^C {^1^H} NMR (100 MHz, acetone-*d*_6_): δ 171.2, 169.0, 70.6, 64.0, 57.0, 53.8, 48.5, 44.6. IR (KBr): 3416, 3021, 2695, 2661, 2564, 1694, 1635, 1436, 1358, 1291, 1235, 1211, 957, 941, 853, 724, 507, 441 cm^–1^. MS, *m/z* (%): 227.0 (10, M - H), 225.0 (100, M – H), 145.0 (10). HRMS (ESI-) *m/z*: [M – H]^−^ calcd for C_10_H_6_O_4_Cl^–^ 224.9960; found 224.9960. Anal. calcd for C_10_H_7_ClO_4_: C, 53.00; H, 3.11. Found: C, 52.74; H, 3.11.

### (1*S*,2*R*,3*R*,4*S*,5*R*,6*R*,7*R*,8*R*)-2,3-Dichlorocubane-1,4-dicarboxylic Acid (**(−)-2a**) and (1*R*,2*S*,3*S*,4*R*,5*S*,6*S*,7*S*,8*S*)-2,3-Dichlorocubane-1,4-dicarboxylic Acid (**(+)-2a**)

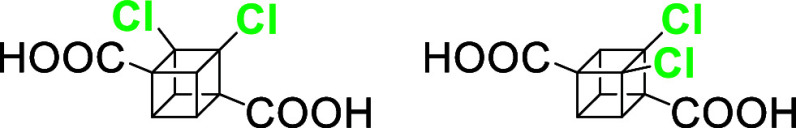



The title compounds were synthesized from racemic **2aMe** (105 mg, 0.363 mmol) and LiOH·H_2_O (305 mg, 7.26 mmol) in the mixture of THF (6 mL) and H_2_O (4 mL) according to GP1. Racemic diacid **2a** was obtained as a white crystalline solid (93 mg, 0.356 mmol, 98%). Resolution of enantiomers was performed using HPLC (IE, isopropyl alcohol/*n*-heptane = 5/95 with 0.1% TFA, flow rate = 20 mL/min, l = 220 nm) *t*_R_ = 7.7 min (**(−)-2a**), 9.0 min (**(+)-2a**).

Mp >250 °C (dec.). ^1^H NMR (400 MHz, acetone-*d*_6_): δ 4.45–4.43 (m, 2H), 4.29–4.27 (m, 2H). ^13^C {^1^H} NMR (100 MHz, acetone-*d*_6_): δ 167.9, 75.1, 61.8, 54.6, 45.8. IR (KBr): 3426, 3022, 2993, 2922, 2852, 2720, 2608, 2532, 1696, 1429, 1324, 1231, 1202, 940 cm^–1^. MS, *m/z* (%): 259.0 (100, M – H, most intense peak of isotope cluster), 215.0 (10, M-COOH, most intense peak of isotope cluster), 179.0 (23, M-COOH-Cl, most intense peak of isotope cluster), 135.0 (22, M-2×COOMe-Cl, most intense peak of isotope cluster). HRMS (ESI−) *m/z*: [M – H]^−^ calcd for C_10_H_5_Cl_2_O_4_^–^ 258.9570; found 258.9571. Anal. calcd for C_10_H_6_Cl_2_O_4_: C, 46.01; H, 2.32. Found: C, 45.85; H, 2.27. **(+)-2a**: [α]^20^_589_ = +5.4 (93% *ee*). **(−)-2a**: [α]^20^_589_ = −4.7 (>99% *ee*).

### 2,5-Dichlorocubane-1,4-dicarboxylic Acid (**2b**)

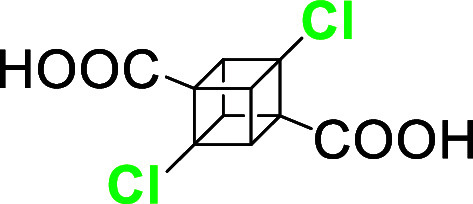



The title compound was synthesized from **2bMe** (19 mg, 0.066 mmol) and LiOH·H_2_O (55 mg, 1.31 mmol) in the mixture of THF (3 mL) and H_2_O (2 mL) according to **GP1**. Diacid **2b** was obtained as a white crystalline solid (17 mg, 0.065 mmol, 99%).

Mp >250 °C (dec.). ^1^H NMR (400 MHz, acetone-*d*_6_): δ 4.44 (s, 4H). ^13^C {^1^H} NMR (100 MHz, acetone-*d*_6_): δ 167.7, 71.0, 60.4, 53.6. IR (KBr): 3432, 3048, 2854, 2560, 1703, 1635, 1452, 1375, 1304, 1231, 1214, 1121, 966, 726, 500, 487 cm^–1^. MS, *m/z* (%): 259.0 (100, M – H, most intense peak of isotope cluster), 215.0 (5, M-COOH, most intense peak of isotope cluster), 179.0 (65, M-COOH-Cl, most intense peak of isotope cluster), 135.0 (55, M-2 × COOH-Cl, most intense peak of isotope cluster). HRMS (ESI−) *m/z*: [M – H]^−^ calcd for C_10_H_5_Cl_2_O_4_^–^ 258.9570; found 258.9571. Anal. calcd for C_10_H_6_Cl_2_O_4_: C, 46.01; H, 2.32. Found: C, 46.15; H, 2.47.

### 2,6-Dichlorocubane-1,4-dicarboxylic Acid (**2c**)

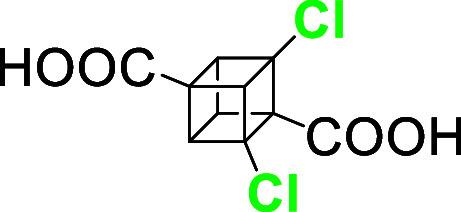



The title compound was synthesized from **2cMe** (95 mg, 0.329 mmol) and LiOH·H_2_O (275 mg, 6.57 mmol) in the mixture of THF (10 mL) and H_2_O (5 mL) according to **GP1**. Diacid **2c** was obtained as a white crystalline solid (85 mg, 0.326 mmol, 99%).

Mp >250 °C (dec.). ^1^H NMR (400 MHz, acetone-*d*_6_): δ 4.61–4.59 (m, 1H), 4.43–4.40 (m, 2H), 4.32–4.29 (m, 1H). ^13^C {^1^H} NMR (100 MHz, acetone-*d*_6_): δ 169.9, 165.9, 69.9, 67.0, 65.2, 57.7, 50.2, 41.0. IR (KBr): 3046, 3024, 2976, 2832, 2686, 2550, 2520, 1701, 1636, 1453, 1368, 1302, 1230, 1207, 957, 812, 732, 521, 437 cm^–1^. MS, *m/z* (%): 259.0 (100, M – H, most intense peak of the isotope cluster), 215.0 (13, M-COOH, most intense peak of the isotope cluster), 179.0 (12, M-COOH-Cl, most intense peak of the isotope cluster), 135.0 (12, M-2×COOH-Cl, most intense peak of the isotope cluster). HRMS (ESI-) *m/z*: [M – H]^−^ calcd for C_10_H_5_Cl_2_O_4_^–^ 258.9570; found 258.9571. Anal. calcd for C_10_H_6_Cl_2_O_4_: C, 46.01; H, 2.32. Found: C, 45.88; H, 2.32.

## Data Availability

The data underlying this study are available in the published article and its Supporting Information.

## Supporting Information Available

Copies of ^1^H and ^13^C NMR spectra of all new compounds, ORTEP views of a single molecule and packing in **1Me**, **2aMe**, **2bMe**, and **2cMe**, parameters of single crystals, relative strain energies, and acidities of all analyzed dicarboxylic acids (PDF)
Geometries and energies of all structures used in Schemes 3 and 4 and Scheme S1 (paths.xyz) (XYZ)
The optimized geometries of all halogenated cubanes that were used for computation of the strain energies (strain.xyz) (XYZ)
Optimized geometries of all halogenated cubanes (mp2_opt.xyz) (XYZ)
Geometry used for calculation of the ECD spectrum of **(−)-2a** (RR-2a_DFT.xyz) (XYZ)

## Supplementary Material

jo2c02872_si_001.pdf

jo2c02872_si_002.xyz

jo2c02872_si_003.xyz

jo2c02872_si_004.xyz

jo2c02872_si_005.xyz
