# Supplementary material for: Chlorinated Cubane-1,4-dicarboxylic Acids
Source: J Org Chem. 2023 Feb 1;89(16):11100–8. doi: 10.1021/acs.joc.2c02872 (PMC11334191; doi:10.1021/acs.joc.2c02872)

Supplementary Information
Chlorinated Cubane-1,4-dicarboxylic Acids

Adéla Křížková,[a] Guillaume Bastien,[a] Igor Rončević,[a] Ivana Císařová,[b] Jiří Rybáček,[a] Václav Kašička,[a] and Jiří Kaleta[a,]*

[a] *Institute of Organic Chemistry and Biochemistry of the Czech Academy of Sciences, Flemingovo nám. 2, 160 00 Prague 6, Czech Republic.*
[b] *Department of Inorganic Chemistry, Faculty of Science, Charles University in Prague, Hlavova 2030, 12840 Prague 2, Czech Republic.*

Table of Contents

Experimental Section......*S2-S7*
Parameters of Single Crystals of **1Me**, **2aMe**, **2bMe**, and **2cMe**......S2
Relative Strain Energies......S3
Alternative Reaction Pathway towards **1Me•**......S4
DSC Traces for **0**, **1**, **2a**, **2b**, **2c**, **0Me**, **1Me**, **2aMe**, **2bMe**, and **2cMe**......S5
Determination of pK_a......S6
Acidities of **0**, **1**, **2a**, **2b**, and **2c** in Water......S7
Separation of Enantiomers......S8
Solution ^{1}H and ^{13}C NMR Spectra......*S9 – S68*
Compound **0Me**......S9
Compound **1Me**......S15
Compound **2aMe**......S21
Compound **2bMe**......S27
Compound **2cMe**......S33
Compound **0**......S39
Compound **1**......S45
Compound **2a**......S51
Compound **2b**......S57
Compound **2c**......S63
ORTEP Visualizations......*S69-S76*
Compound **1Me**......S69
Compound **2aMe**......S71
Compound **2bMe**......S73
Compound **2cMe**......S75

Corresponding Author
Jiří Kaleta (🖂 jiri.kaleta@uochb.cas.cz)

Parameters of Single Crystals

Table S1. Parameters of Single Crystals of 1Me, 2aMe,[a] 2bMe, and 2cMe.

Cmpd.	Crystal System	Space Group	Cell Lengths (Å)	Cell Angles (°)
**1Me**	monoclinic	P $2_1/c$	*a* 7.8442(6)	*α* 90
			b 5.8184(4)	*β* 101.194(3)
			c 12.4742(12)	*γ* 90
**2aMe**	triclinic	P -1	*a* 6.4059(8)	*α* 85.081(6)
			b 8.0838(9)	*β* 83.592(6)
			c 11.8351(13)	*γ* 85.342(6)
**2bMe**	triclinic	P -1	*a* 6.0085(4)	*α* 82.394(2)
			b 6.2509(5)	*β* 82.965(2)
			c 7.7863(6)	*γ* 84.138(2)
**2cMe**	triclinic	P -1	*a* 5.9206(4)	*α* 101.370(3)
			b 8.1195(5)	*β* 92.835(2)
			c 12.7766(8)	*γ* 96.793(2)

[a] Racemic mixture of **(-)-2aMe** and **(+)-2aMe**.

Calculated Relative Strain Energies

Table S2. Calculated Relative Strain Energies (kcal/mol) in Cubane-1,4-dicarboxylic Acids at B3LYP-D3BJ/6-311+G(d,p), DLPNO-SCS-MP2/def2-PVQZ, DLPNO-CCSD(T)/cc-PVQZ at both B3LYP and SCS-MP2 optimized geometries, and DLPNO-CCSD(T)-F12/cc-PVTZ at B3LYP (to check for basis set completeness). In the case of **6X** compounds, calculations with F12 corrections were not tractable.

Cmpd.	Strain Energy (kcal/mol)[a]				
	B3LYP-D3BJ	CCSD(T) @B3LYP	CCSD(T)-F12 @B3LYP	SCS-MP2	CCSD(T) @SCS-MP2
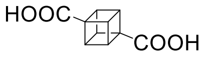 **0**	**0.00**	**0.00**	**0.00**	**0.00**	**0.00**
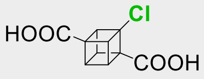 **1**	**1.15**	**1.31**	**1.25**	**1.38**	**1.47**
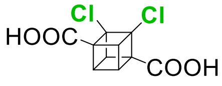 **(-)-2a**	**3.61**	**3.72**	**3.56**	**3.74**	**4.10**
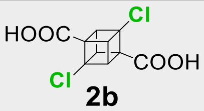 **2b**	**3.02**	**3.21**	**3.09**	**3.29**	**3.56**
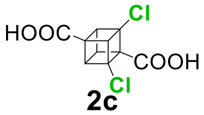 **2c**	**4.49**	**4.93**	**4.72**	**4.76**	**5.28**
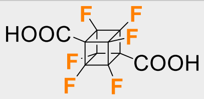 **6F**	**58.34**	**56.82**		**59.01**	**57.52**
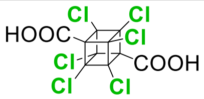 **6Cl**	**23.15**	**22.41**		**22.24**	**23.71**
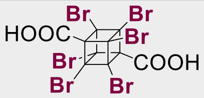 **6Br**	**29.54**	**12.33**		**13.65**	**16.68**
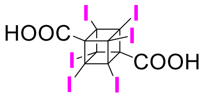 **6I**	**12.35**	**-0.20**		**-2.23**	**3.99**

[a] Only the **(-)-2a** enantiomer is shown for clarity.

Alternative Reaction Pathway towards 1Me$^•$

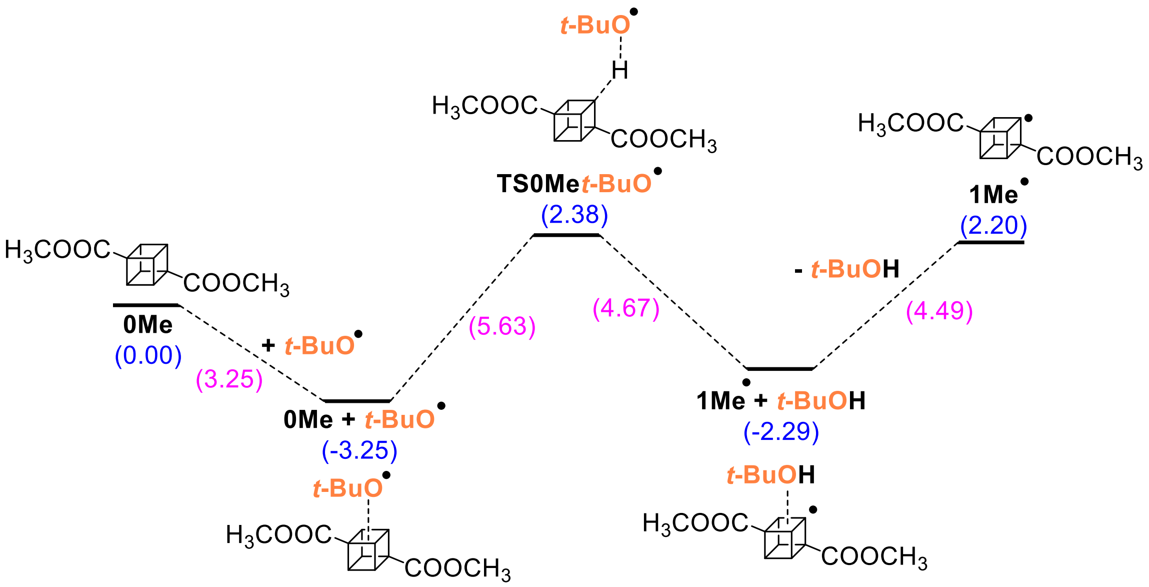

Scheme S1. Alternative Reaction Pathway for the Formation of **1Me$^•$**. The relative energies including ZPVE (blue) as well as the differences between two neighboring states (pink) are in kcal/mol.

Differential Scanning Calorimetry (DSC)

DSC traces were recorded on a DSC 250 apparatus from TA instrument company. All samples were analyzed from +25 °C to +300 °C then back to +25 °C with a ramp of 10 °C/min except compound **0Me** which was heated only up to 280 °C.

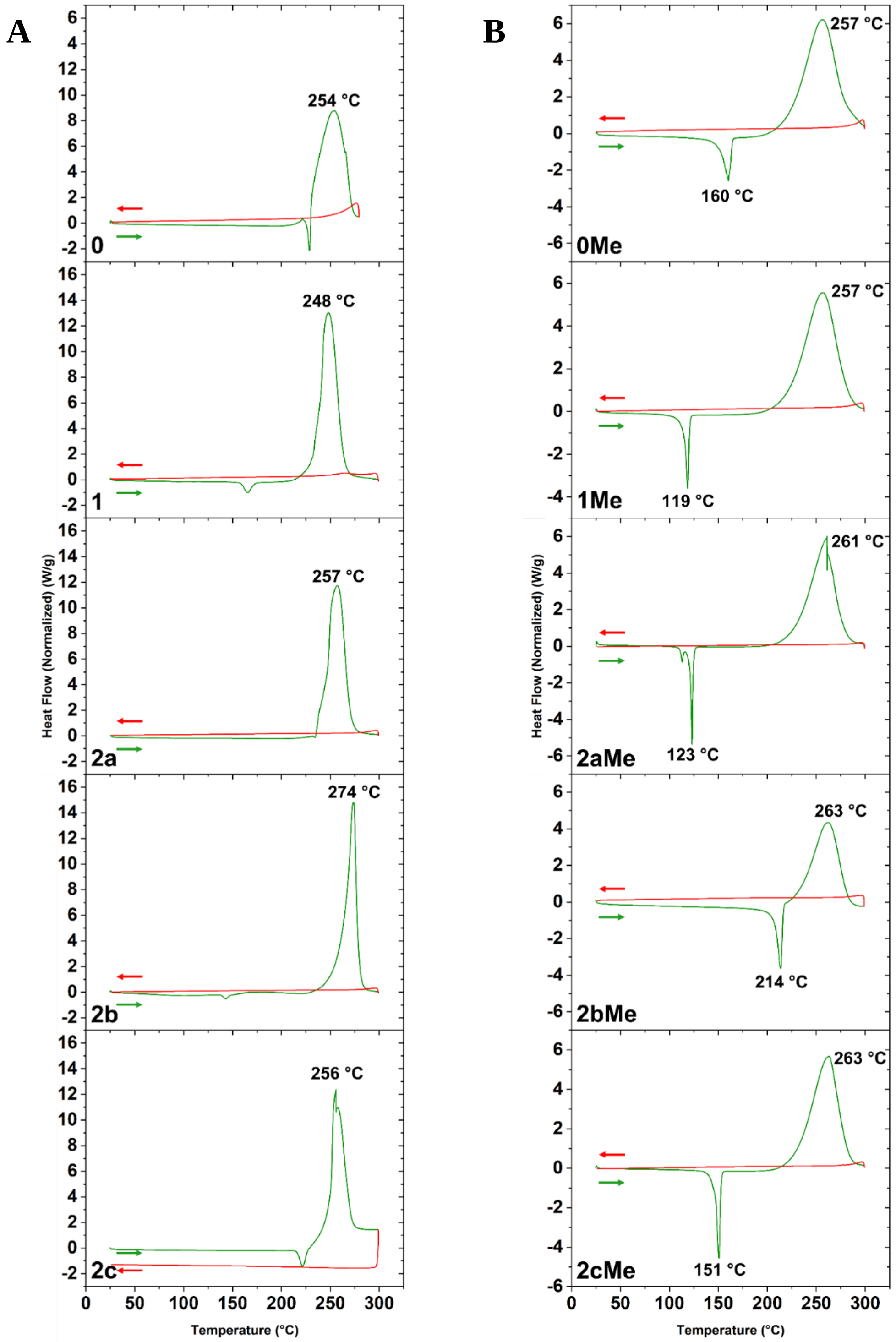

Figure S1. DSC traces for diacids **0**, **1**, **2a**, **2b**, and **2c** (A) and corresponding diesters **0Me**, **1Me**, **2aMe**, **2bMe**, and **2cMe** (B). Exotherms are positive peaks while endotherms are negative.

Determination of pK_a

Capillary electrophoresis (CE) experiments were performed on a CE analyzer P/ACE^{TM} MDQ DNA System (Beckman-Coulter, Fullerton, CA, USA) equipped with a UV−vis spectrophotometric diode array detector set at 195, 200, and 210 nm wavelengths. A bare fused silica capillary with an outer polyimide coating (id/od 50/375 μm, total/effective (to the detector) length 398/297 mm was provided by Polymicro Technologies (Phoenix, AZ, USA).

First, the mixed acidity constants, pK_a^{mix}, (related to the activity of hydroxonium cations and 25 mM ionic strength) and the ionic mobilities of the mono- and divalent anions of the analyzed dicarboxylic acids (μ_{1-} and μ_{2-}) were determined by nonlinear regression analysis of the pH dependence of their effective mobilities measured in a series of the background electrolytes (BGEs) within a wide pH range 0.90–6.52, at constant ionic strength (25 mM) and temperature (25°C). Then, the pK_a^{mix} values were recalculated to the thermodynamic acidity constants pK_a^{th} using the Debye-Hückel theory as described in previously published article.1 The mixed and thermodynamic pK_a values and ionic mobilities of the analyzed acids are presented in Table S3. The pH dependences of the effective mobilities of the analyzed compounds are shown in Figure S2.

Table S3. Experimentally Determined Acidity Constants (pK_a) of **0**, **1**, **2a**,[a] **2b**, and **2c**.[b]

Compd.	p$K_{a,1}^{th}$	p$K_{a,2}^{th}$	p$K_{a,1}^{mix}$	p$K_{a,2}^{mix}$	μ_{1-} ($10^{-9}m^2V^{-1}s^{-1}$)	μ_{2-} ($10^{-9}m^2V^{-1}s^{-1}$)
0	**3.63 ± 0.03**	**4.76 ± 0.04**	3.56 ± 0.03	4.56 ± 0.04	-18.0 ± 0.0	-35.1 ± 0.3
1	**3.17 ± 0.04**	**4.09 ± 0.05**	3.10 ± 0.04	3.89 ± 0.05	-18.0 ± 0.0	-34.3 ± 0.3
2a	**2.70 ± 0.04**	**3.73 ± 0.05**	2.63 ± 0.04	3.52 ± 0.05	-18.0 ± 0.0	-34.5 ± 0.3
2b	**2.73 ± 0.04**	**3.68 ± 0.05**	2.66 ± 0.04	3.48 ± 0.05	-18.0 ± 0.0	-34.4 ± 0.3
2c	**2.71 ± 0.03**	**3.83 ± 0.04**	2.64 ± 0.03	3.62 ± 0.04	-18.0 ± 0.0	-34.2 ± 0.3

[a] Racemic mixture of **(-)-2a** and **(+)-2a**. [b] pK_a^{th}, the thermodynamic acidity constant (at zero ionic strength); pK_a^{mix}, the mixed acidity constant at ionic strength 25 mM; μ_{1-} and μ_{2-}, the ionic mobilities of the univalent and divalent anionic forms of the analyzed acids. All values are related to the temperature of 25 °C.

1. Šolínová, V.; Kašička, V. "Determination of Acidity Constants and Ionic Mobilities of Polyprotic Peptide Hormones by Capillary Zone Electrophoresis." *Electrophoresis* **2013**, *34*, 2655-2665.

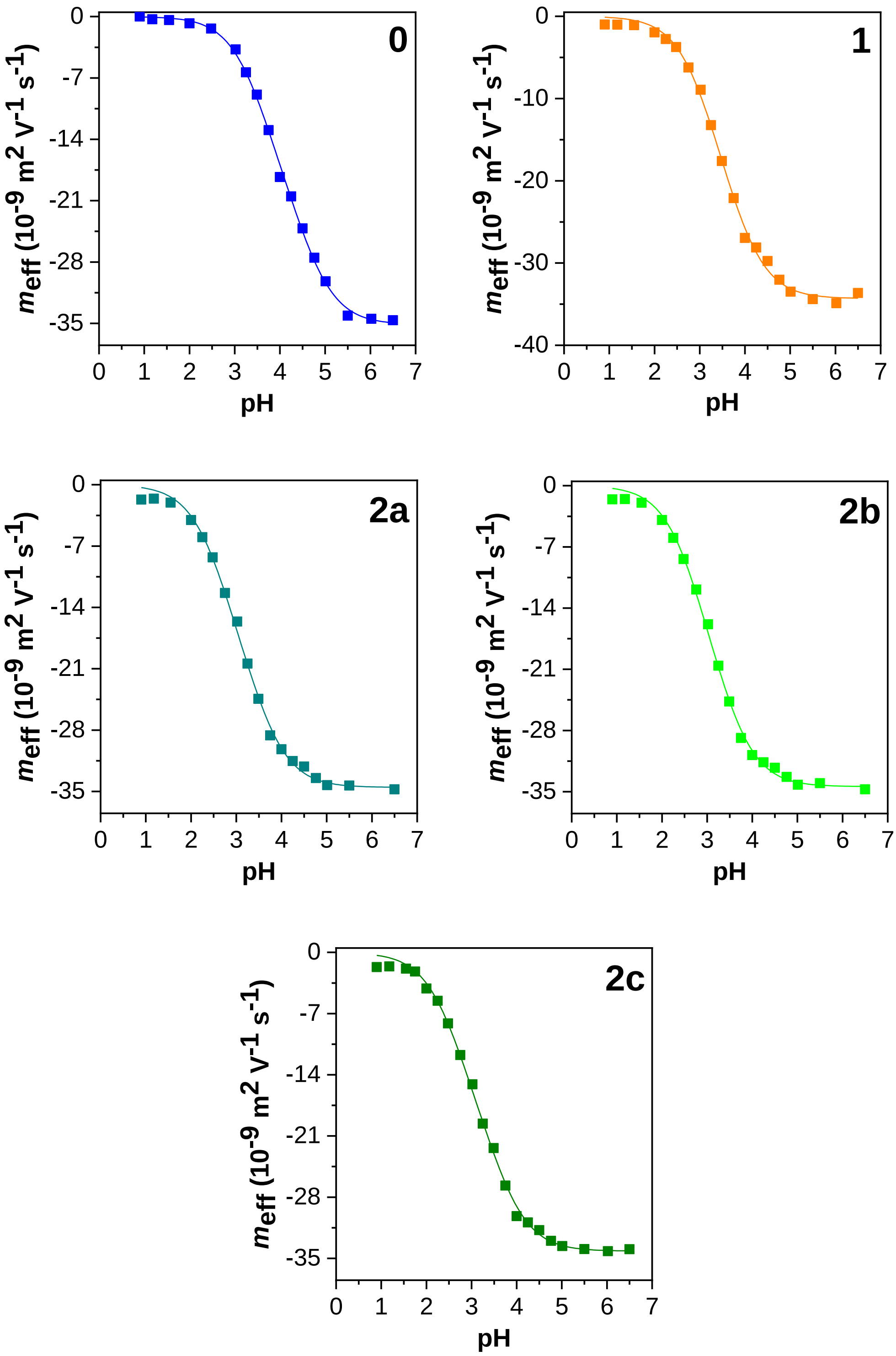

Figure S2. pH dependences of the effective mobilities for diacids **0**, **1**, **2a**, **2b**, and **2c.**

Separation of Enantiomers

Enantiomers of **2a** were separated on Interchim puriFlash 5.250 system using CHIRALPAK IE (5 µm, 20 mm ID x 250 mm) column and 5% isopropyl alcohol in *n*-heptane (0.1% TFA) as mobile phase at a flow rate of 20 mL/min.

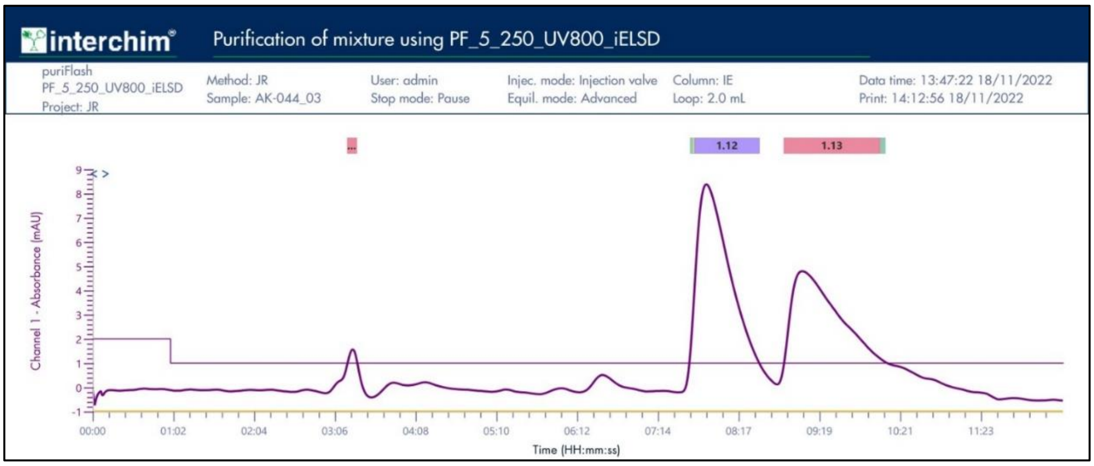

Figure S3. Chromatogram showing separation of **(-)-2a** from **(+)-2a** on a chiral column.

The first eluted enantiomer, **(-)-2a**, was optically pure (>99% *ee*), whereas the second eluted enantiomer, **(+)-2a**, was enantioenriched to ca. 93% *ee* (Figure S4).

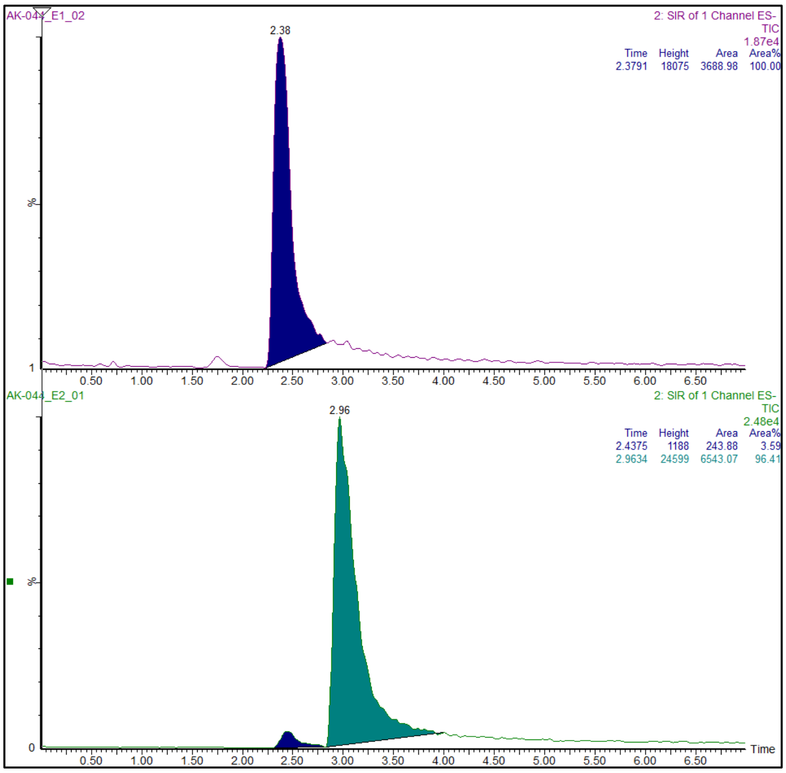

Figure S4. Chromatograms from the UHPSFC/MS system showing the enantiomeric purity of **(-)-2a** (top, >99% *ee*) and **(+)-2a** (bottom, ca. 93% *ee*).

^{1}H NMR (400 MHz, $CDCl_3$): Dimethyl Cubane-1,4-dicarboxylate (**0Me**)

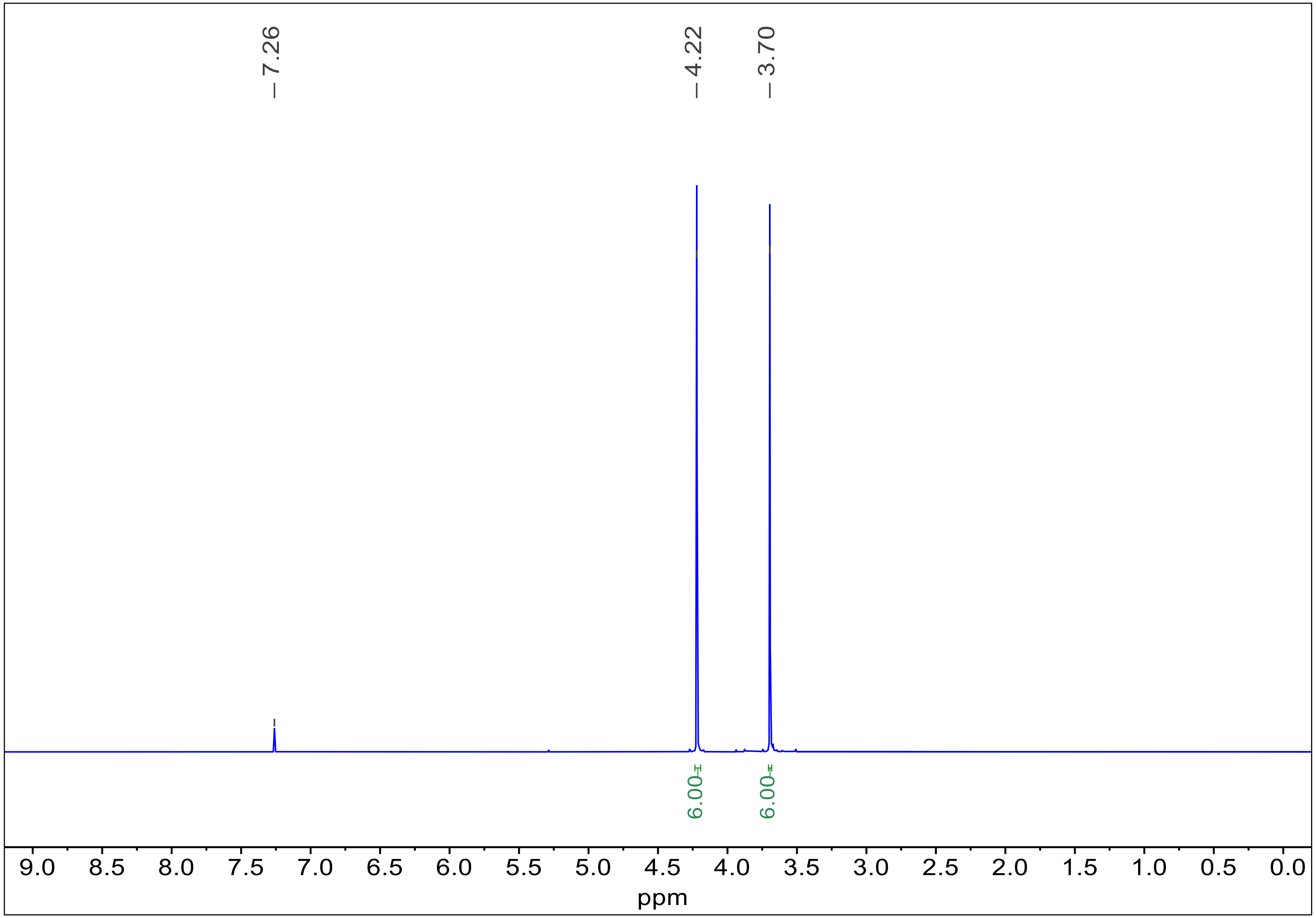

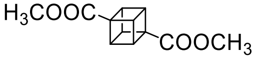

$^{13}C\ \{^{1}H\}$ NMR (100 MHz, $CDCl_3$): Dimethyl Cubane-1,4-dicarboxylate (**0Me**)

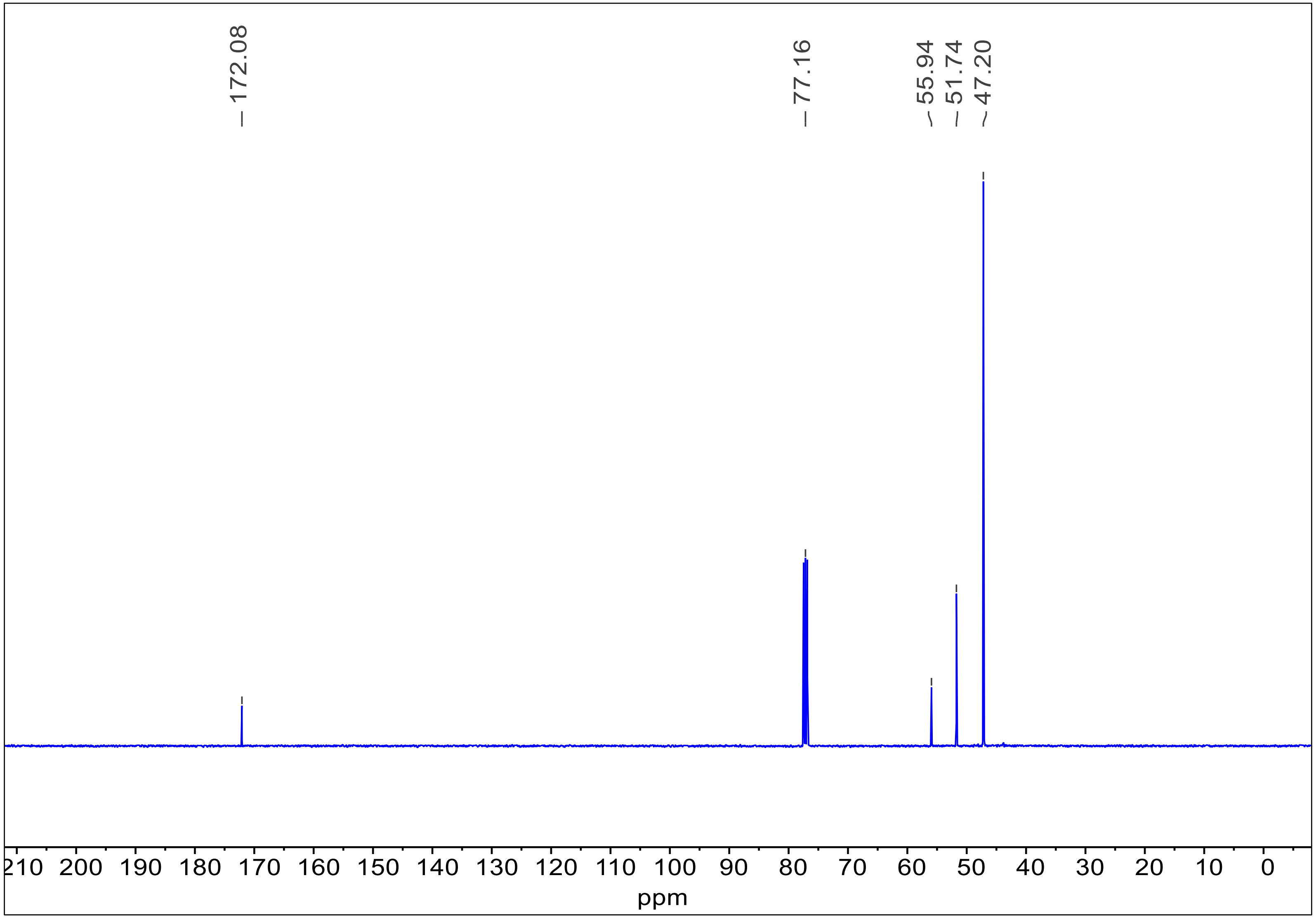

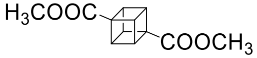

^{13}C APT NMR (100 MHz, $CDCl_3$): Dimethyl Cubane-1,4-dicarboxylate (**0Me**)

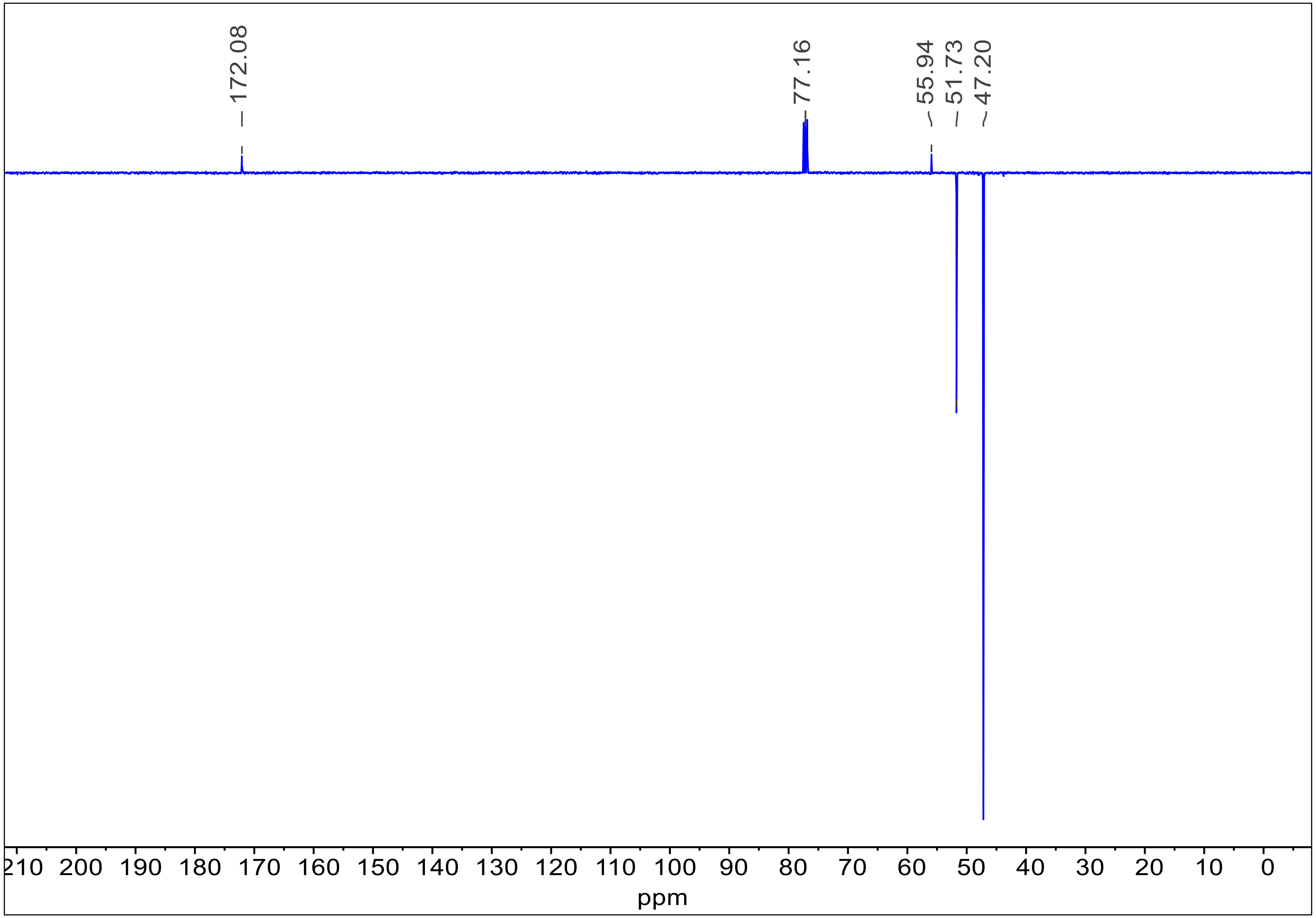

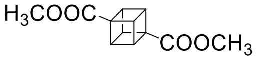

$^{1}H - {}^{1}H$ COSY ($CDCl_3$): Dimethyl Cubane-1,4-dicarboxylate (**0Me**)

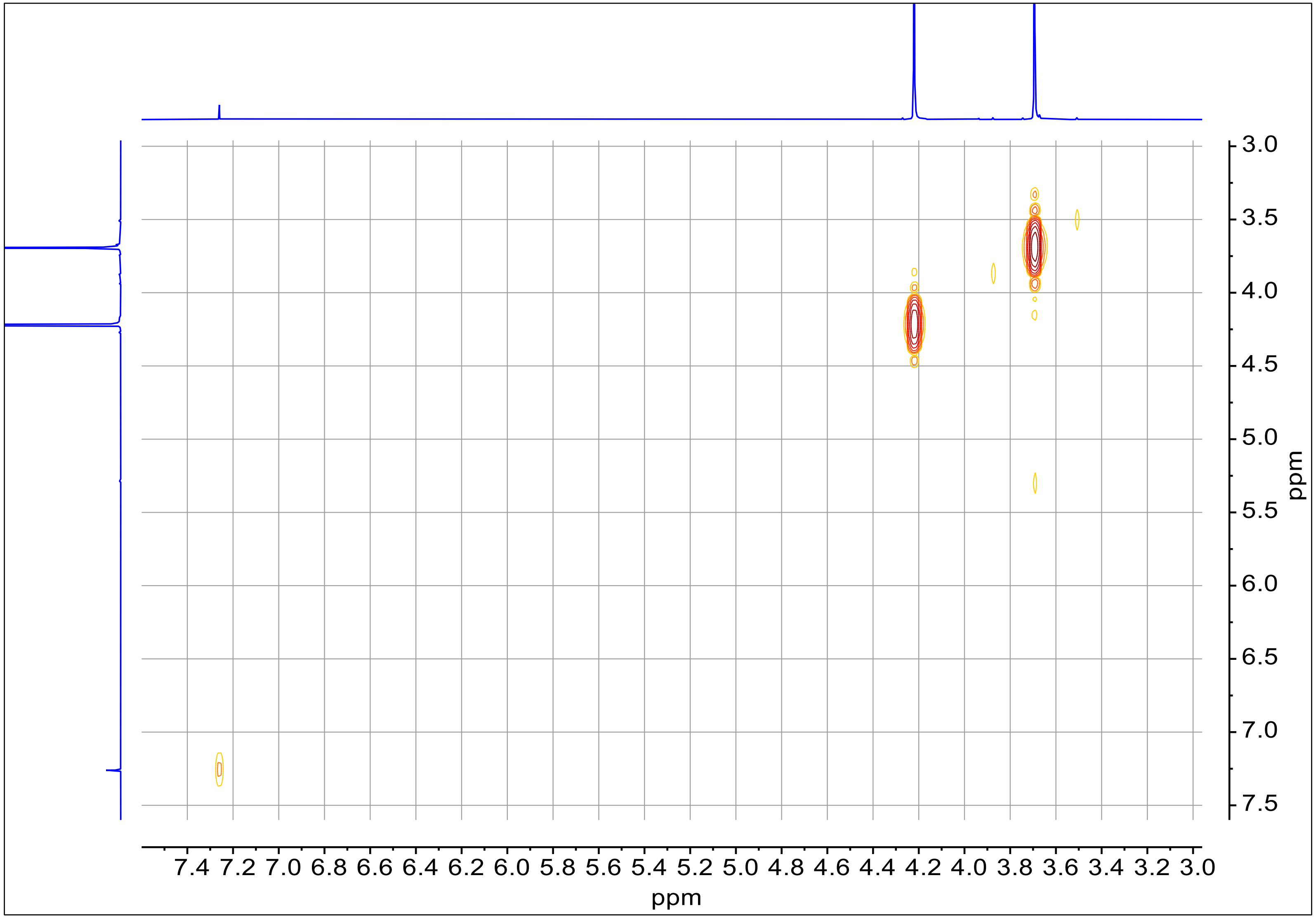

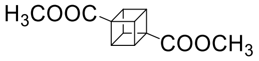

HSQC (CDCl3): Dimethyl Cubane-1,4-dicarboxylate (**0Me**)

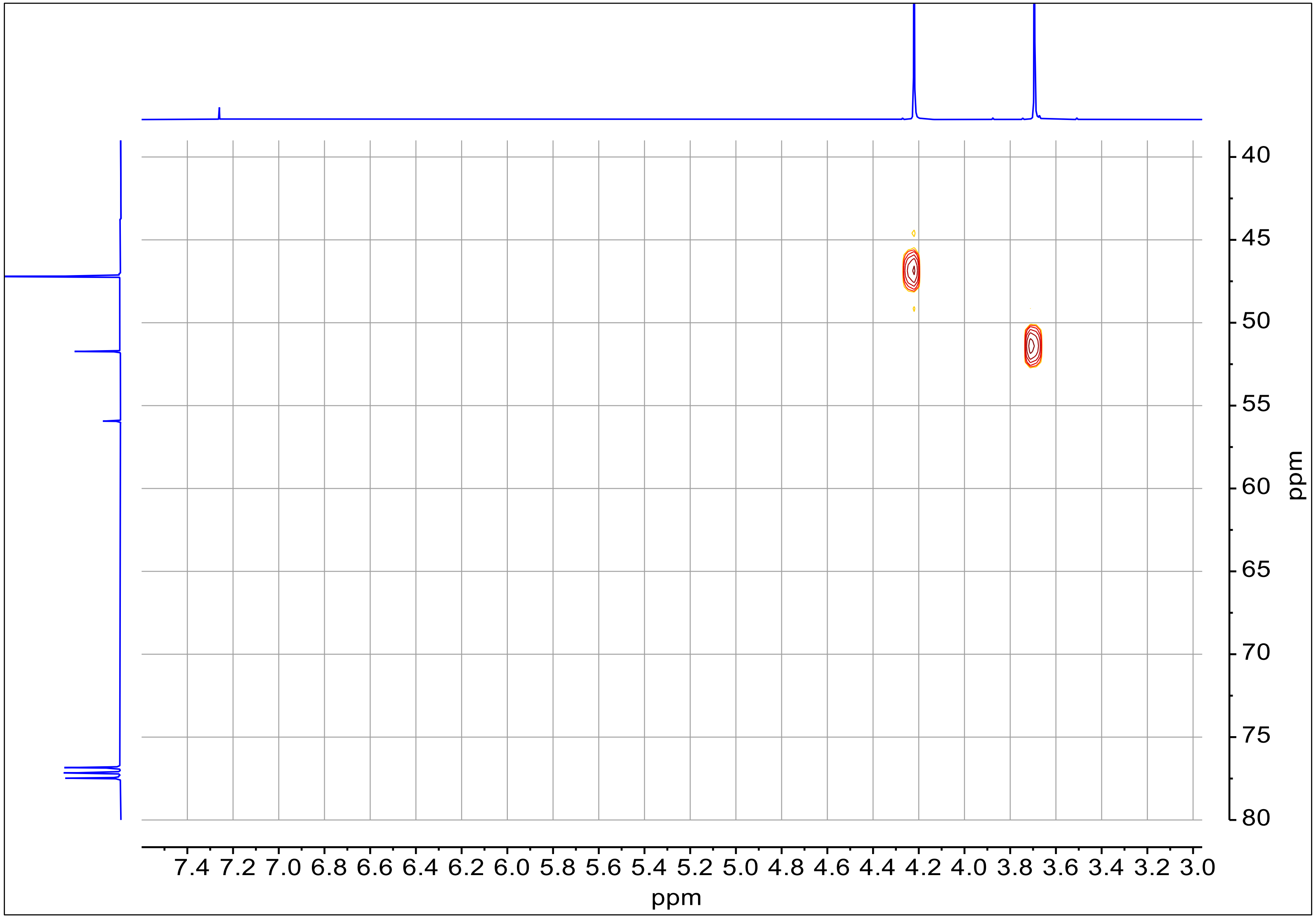

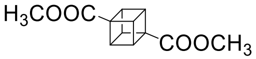

HMBC ($CDCl_3$): Dimethyl Cubane-1,4-dicarboxylate (**0Me**)

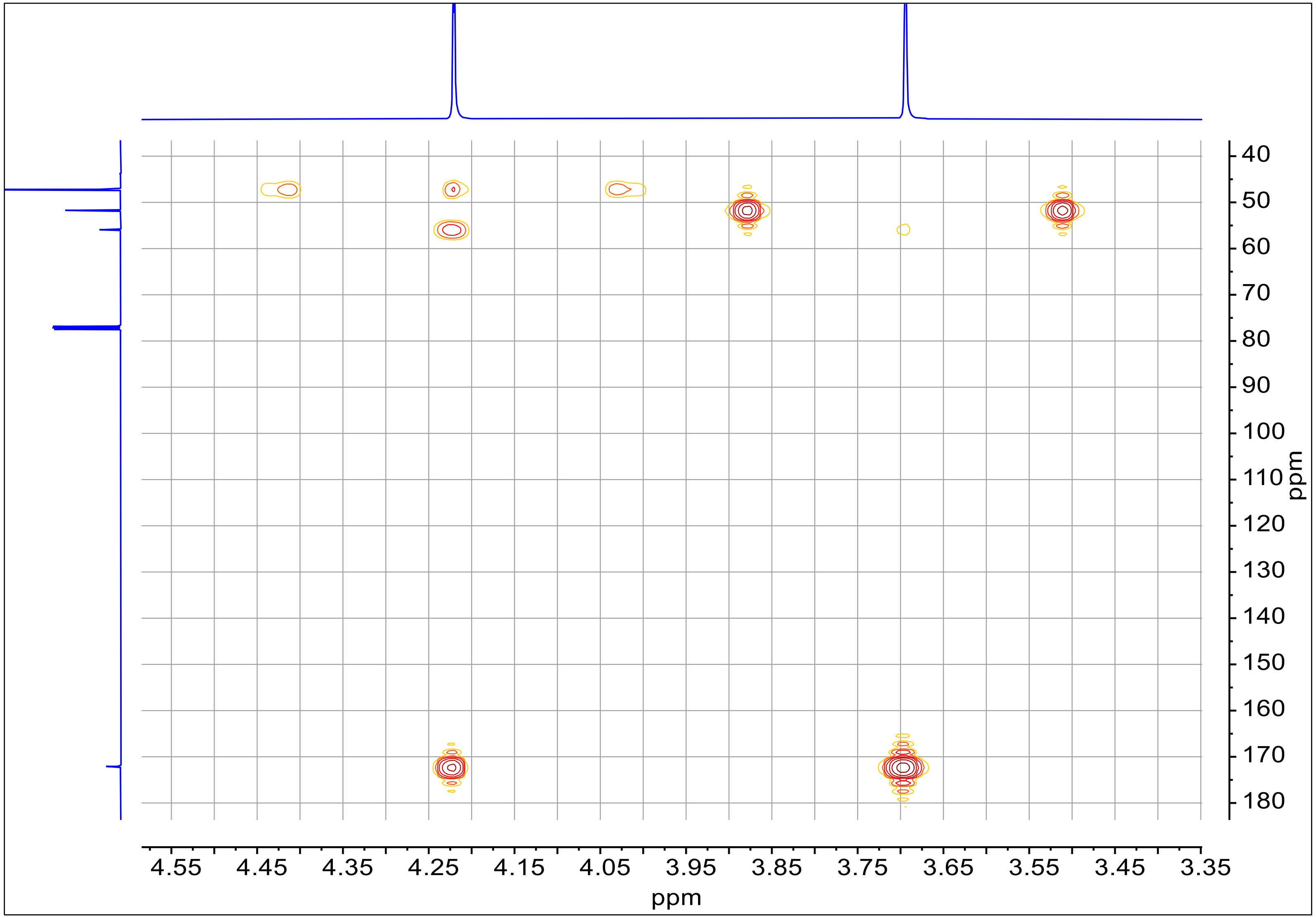

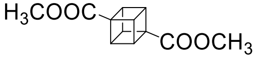

^{1}H NMR (400 MHz, $CDCl_3$): Dimethyl 2-Chlorocubane-1,4-dicarboxylate (**1Me**)

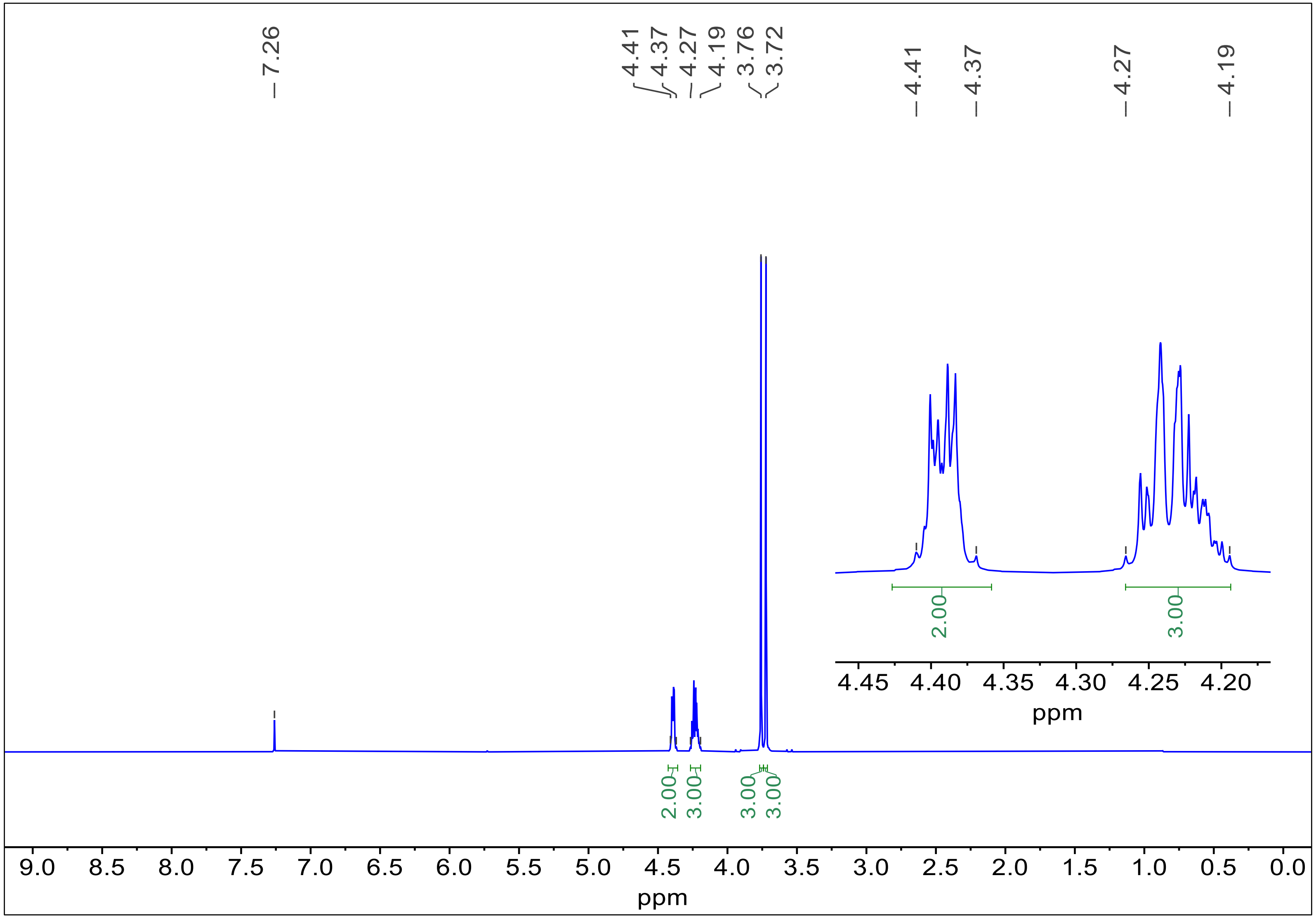

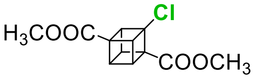

$^{13}C\ \{^{1}H\}$ NMR (100 MHz, $CDCl_3$): Dimethyl 2-Chlorocubane-1,4-dicarboxylate (**1Me**)

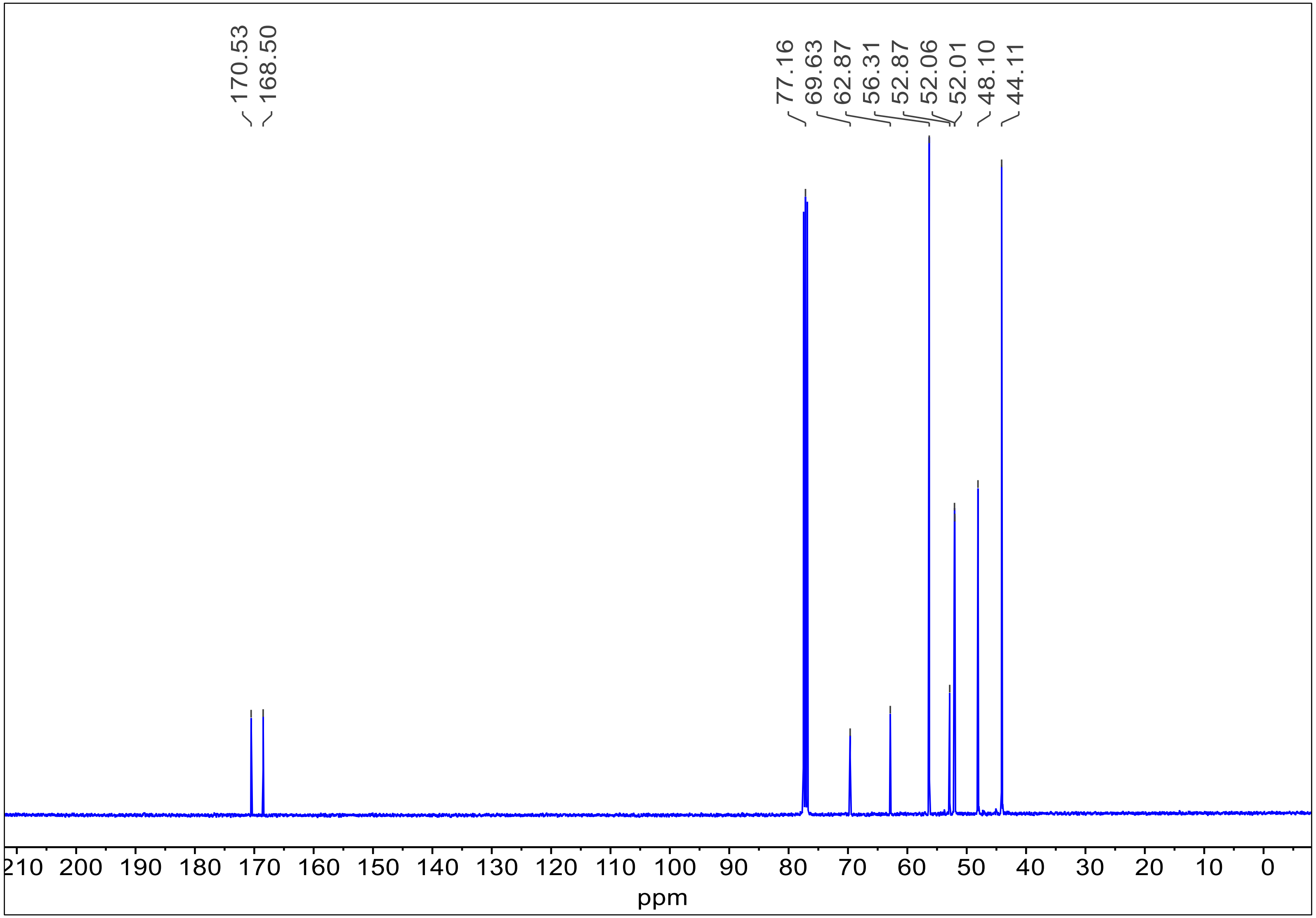

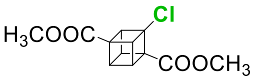

^{13}C APT NMR (100 MHz, $CDCl_3$): Dimethyl 2-Chlorocubane-1,4-dicarboxylate (**1Me**)

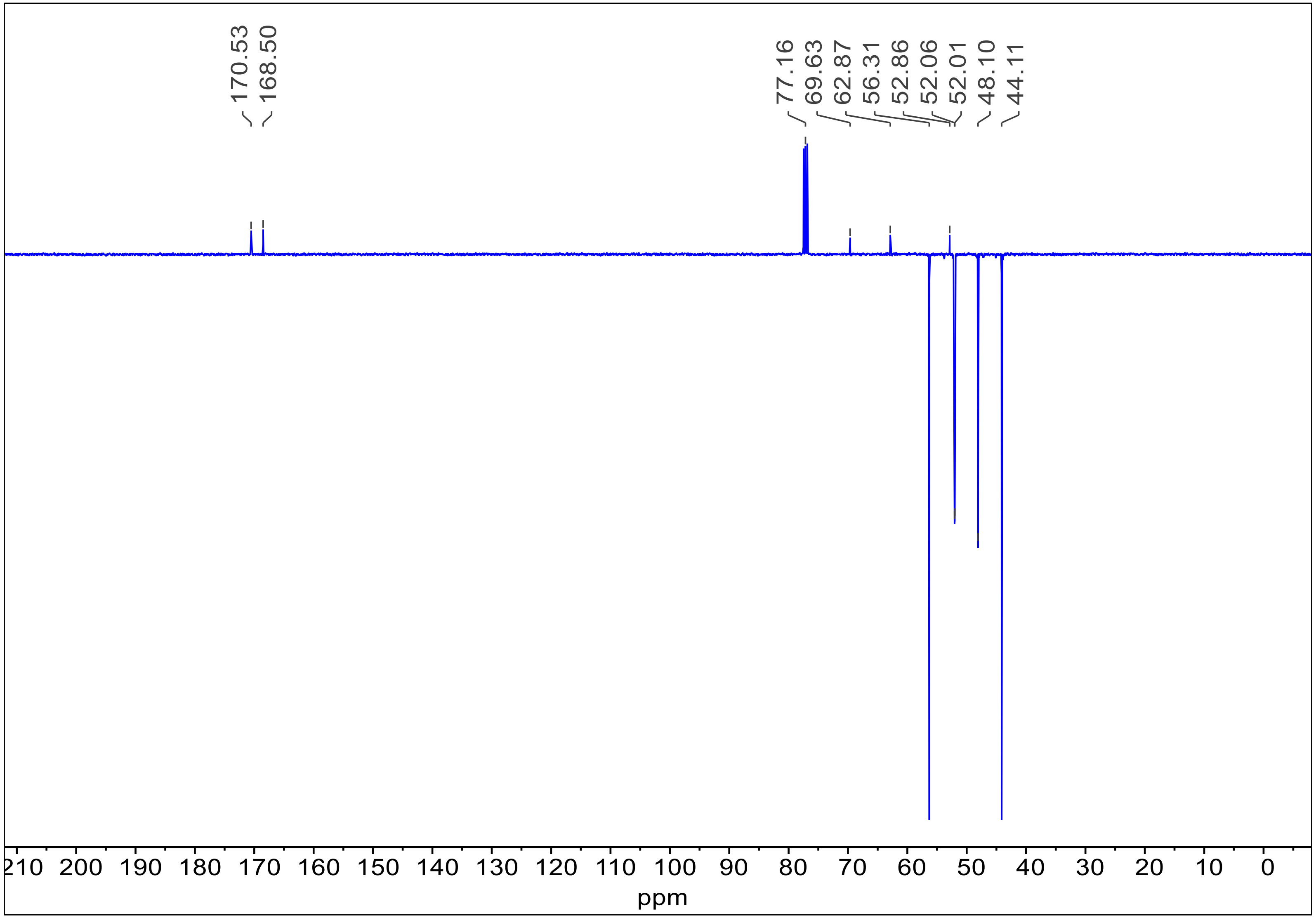

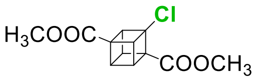

^{1}H – ^{1}H COSY ($CDCl_3$): Dimethyl 2-Chlorocubane-1,4-dicarboxylate (**1Me**)

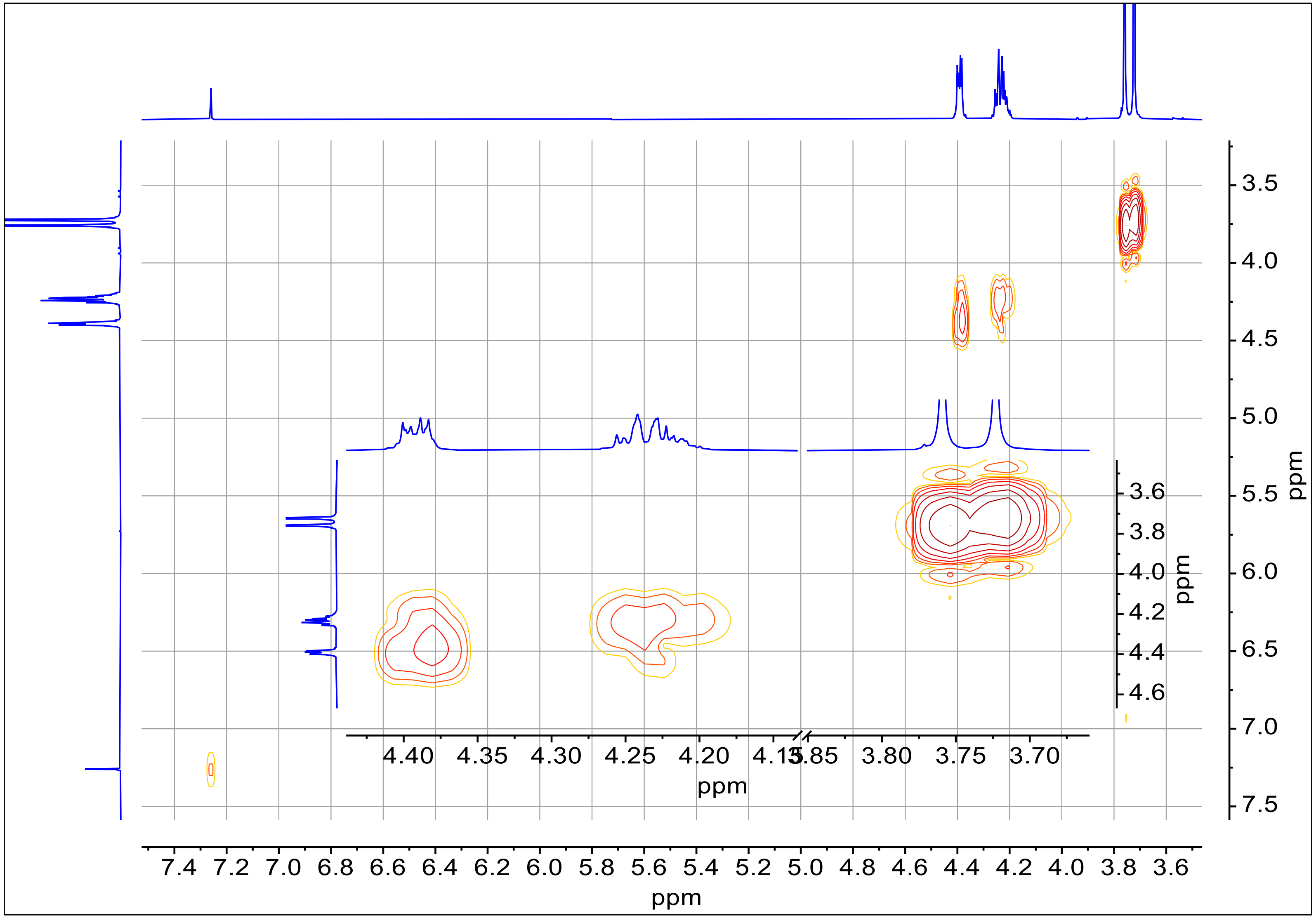

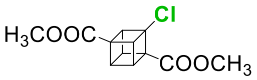

HSQC ($CDCl_3$): Dimethyl 2-Chlorocubane-1,4-dicarboxylate (**1Me**)

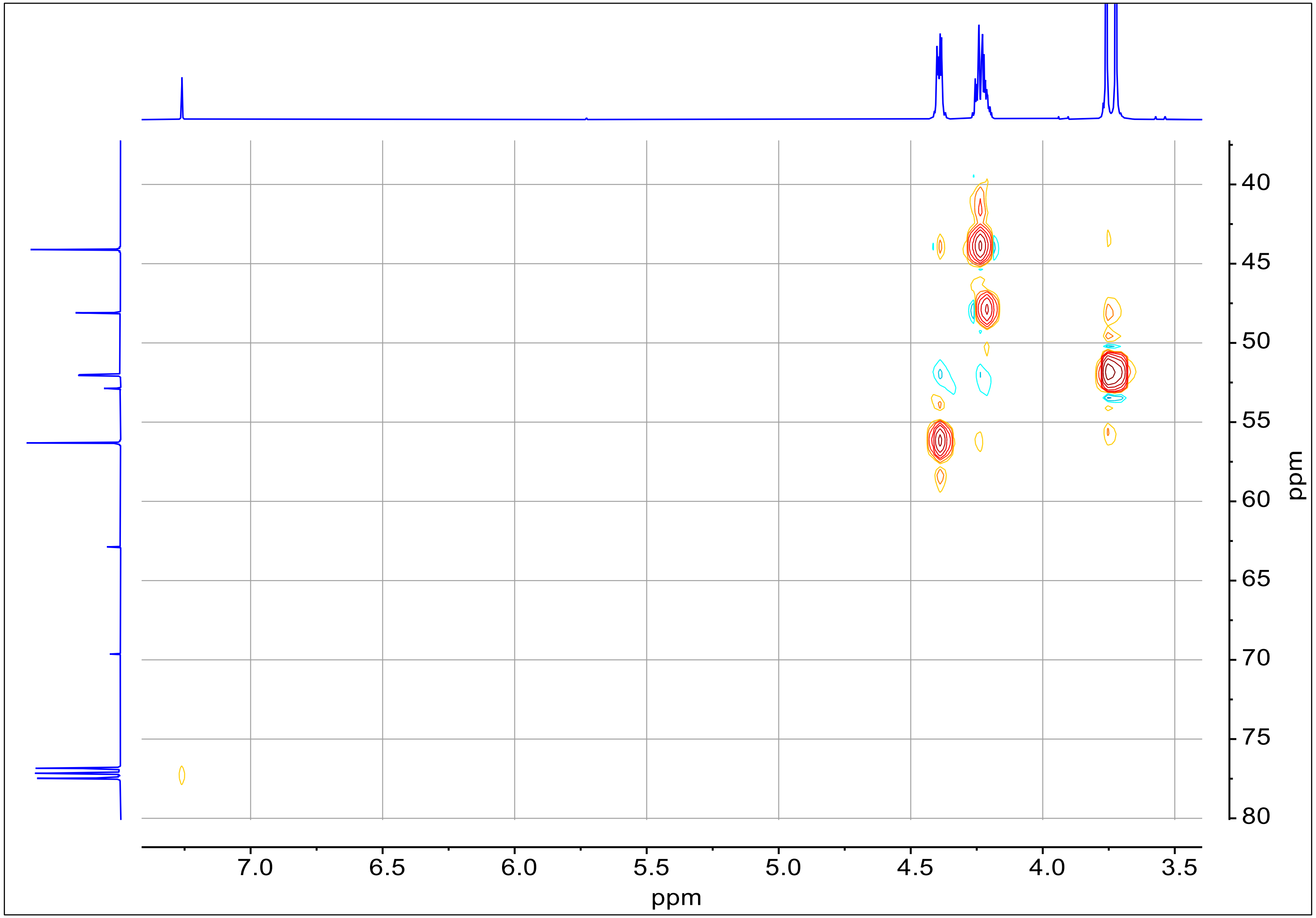

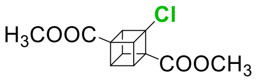

HMBC ($CDCl_3$): Dimethyl 2-Chlorocubane-1,4-dicarboxylate (**1Me**)

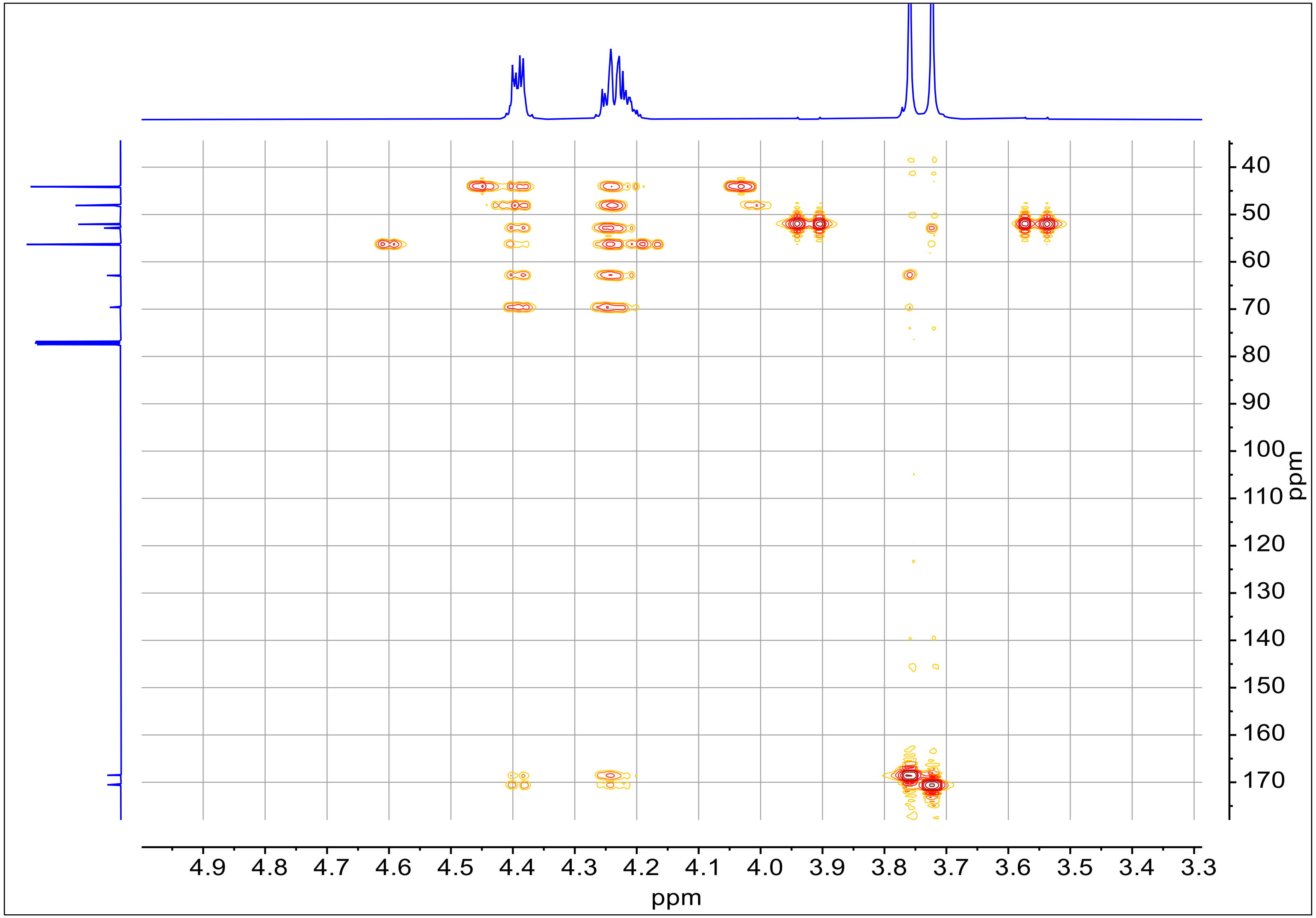

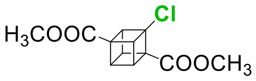

^{1}H NMR (400 MHz, CDCl3): Dimethyl (1*S*,2*R*,3*R*,4*S*,5*R*,6*R*,7*R*,8*R*)-2,3-Dichlorocubane-1,4-dicarboxylate (**(-)-2aMe**) and Dimethyl (1*R*,2*S*,3*S*,4*R*,5*S*,6*S*,7*S*,8*S*)-2,3-Dichlorocubane-1,4-dicarboxylate (**(+)-2aMe**)

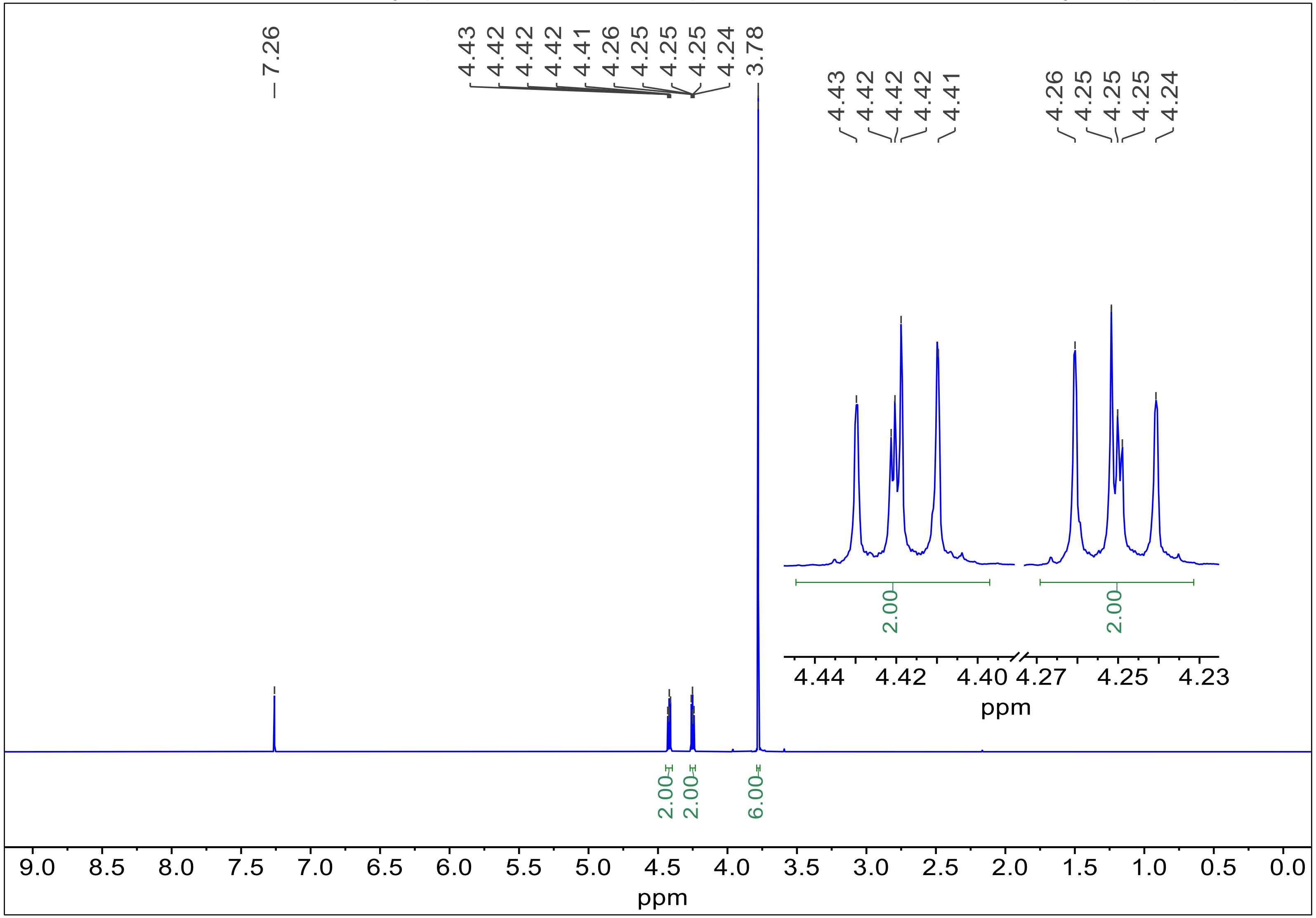

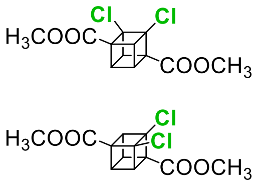

^{13}C {^1H} NMR (100 MHz, $CDCl_3$): Dimethyl (1*S*,2*R*,3*R*,4*S*,5*R*,6*R*,7*R*,8*R*)-2,3-Dichlorocubane-1,4-dicarboxylate (**(-)-2aMe**) and Dimethyl (1*R*,2*S*,3*S*,4*R*,5*S*,6*S*,7*S*,8*S*)-2,3-Dichlorocubane-1,4-dicarboxylate (**(+)-2aMe**)

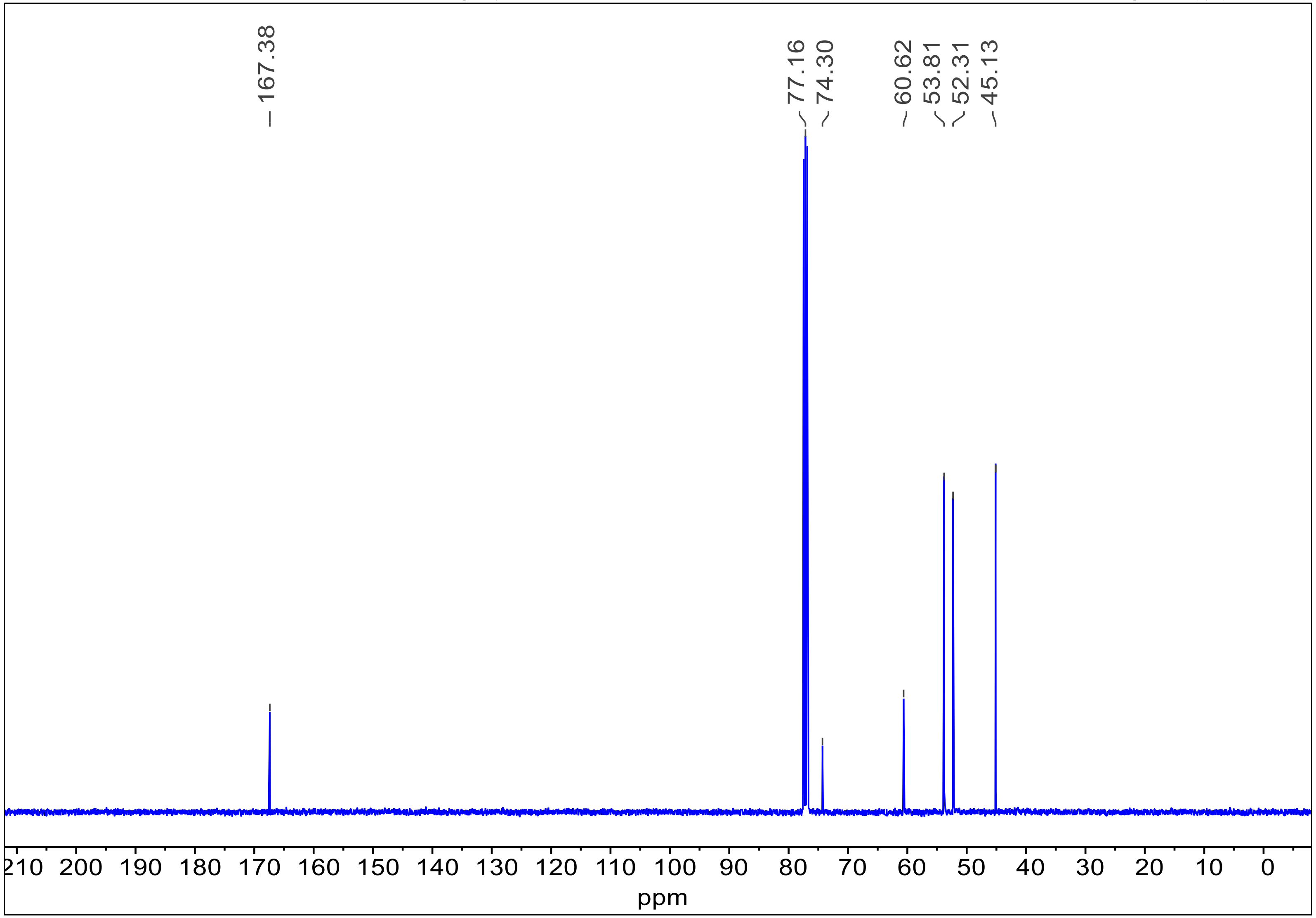

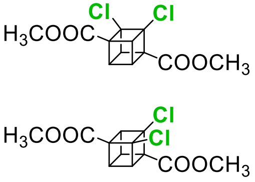

^{13}C APT NMR (100 MHz, $CDCl_3$): Dimethyl (1*S*,2*R*,3*R*,4*S*,5*R*,6*R*,7*R*,8*R*)-2,3-Dichlorocubane-1,4-dicarboxylate (**(-)-2aMe**) and Dimethyl (1*R*,2*S*,3*S*,4*R*,5*S*,6*S*,7*S*,8*S*)-2,3-Dichlorocubane-1,4-dicarboxylate (**(+)-2aMe**)

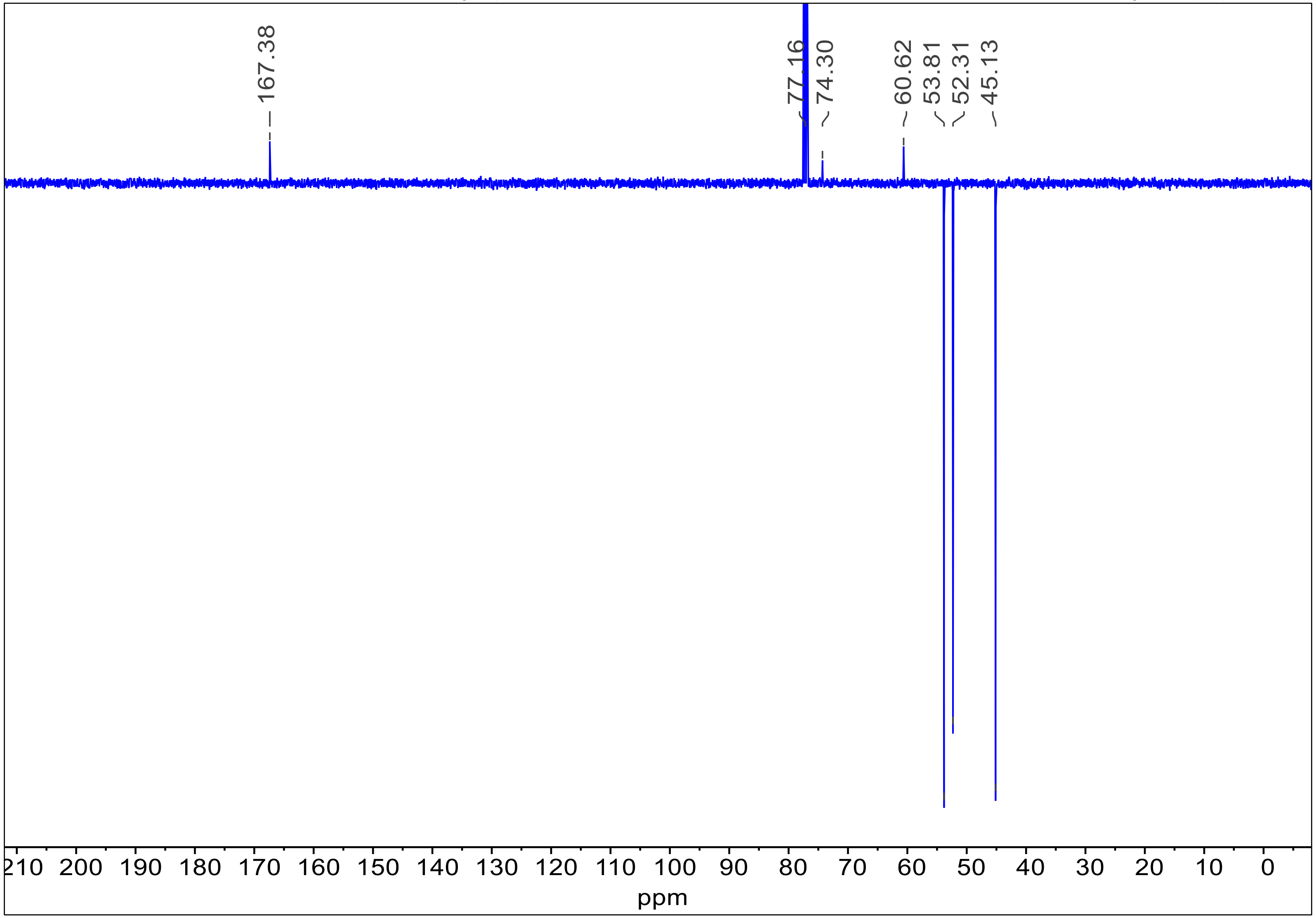

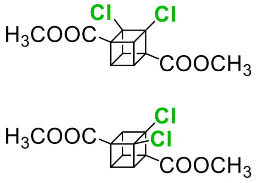

^{1}H – ^{1}H COSY ($CDCl_3$): Dimethyl (1*S*,2*R*,3*R*,4*S*,5*R*,6*R*,7*R*,8*R*)-2,3-Dichlorocubane-1,4-dicarboxylate (**(-)-2aMe**) and Dimethyl (1*R*,2*S*,3*S*,4*R*,5*S*,6*S*,7*S*,8*S*)-2,3-Dichlorocubane-1,4-dicarboxylate (**(+)-2aMe**)

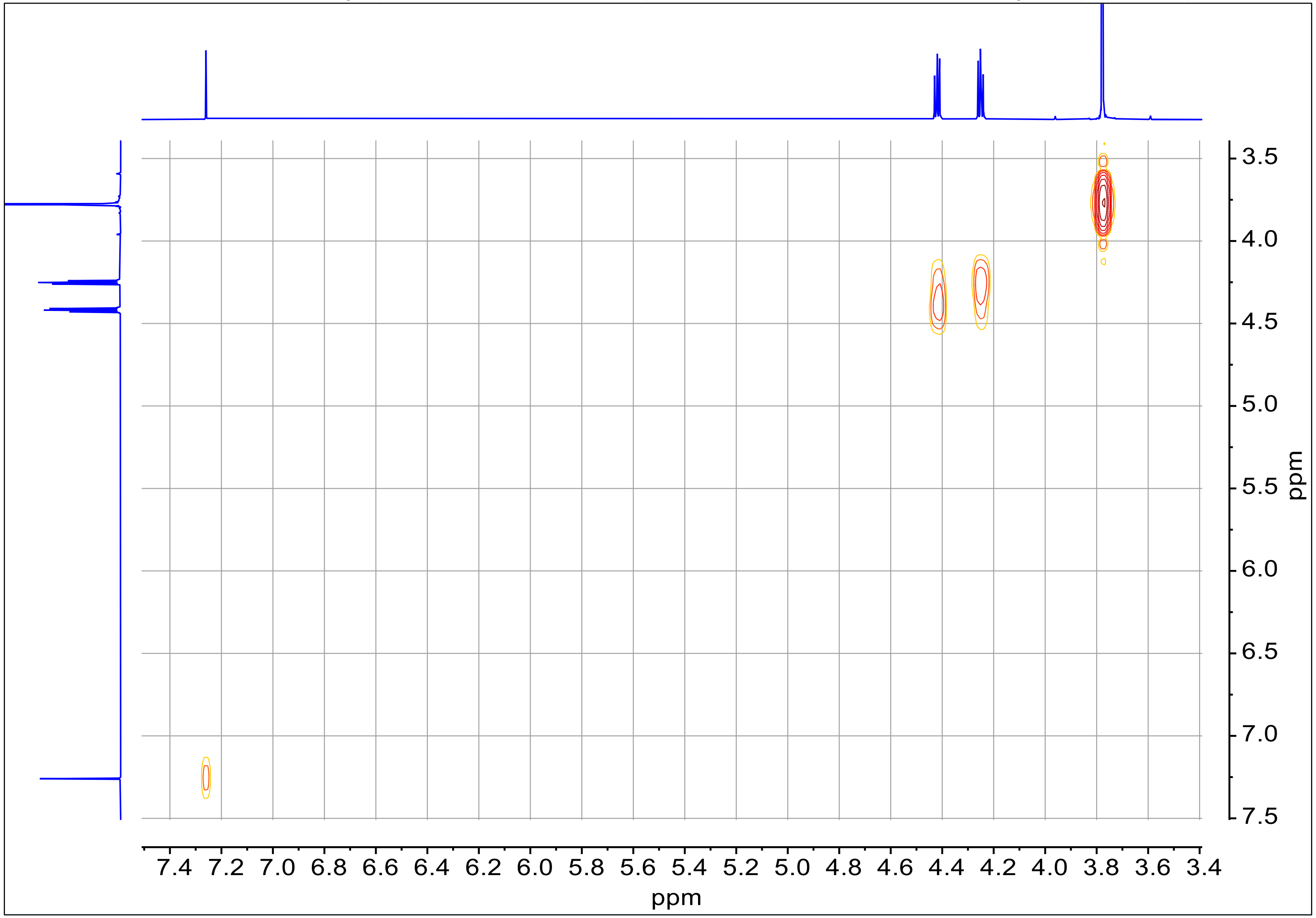

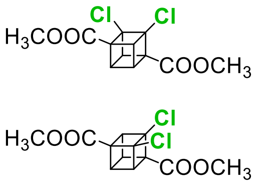

HSQC ($CDCl_3$): Dimethyl (1*S*,2*R*,3*R*,4*S*,5*R*,6*R*,7*R*,8*R*)-2,3-Dichlorocubane-1,4-dicarboxylate (**(-)-2aMe**) and Dimethyl (1*R*,2*S*,3*S*,4*R*,5*S*,6*S*,7*S*,8*S*)-2,3-Dichlorocubane-1,4-dicarboxylate (**(+)-2aMe**)

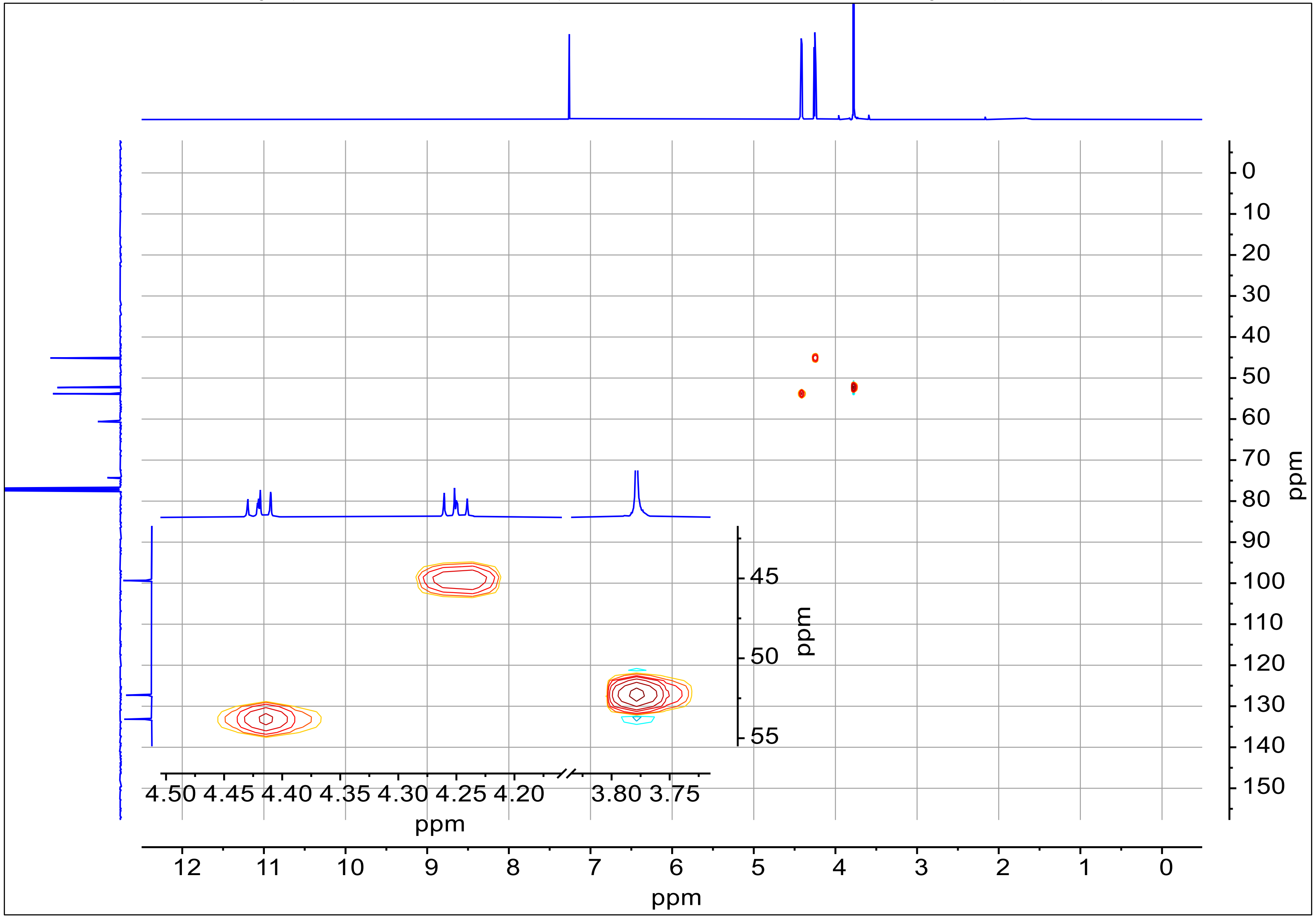

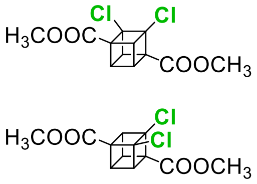

HMBC ($CDCl_3$): Dimethyl (1*S*,2*R*,3*R*,4*S*,5*R*,6*R*,7*R*,8*R*)-2,3-Dichlorocubane-1,4-dicarboxylate (**(-)-2aMe**) and Dimethyl (1*R*,2*S*,3*S*,4*R*,5*S*,6*S*,7*S*,8*S*)-2,3-Dichlorocubane-1,4-dicarboxylate (**(+)-2aMe**)

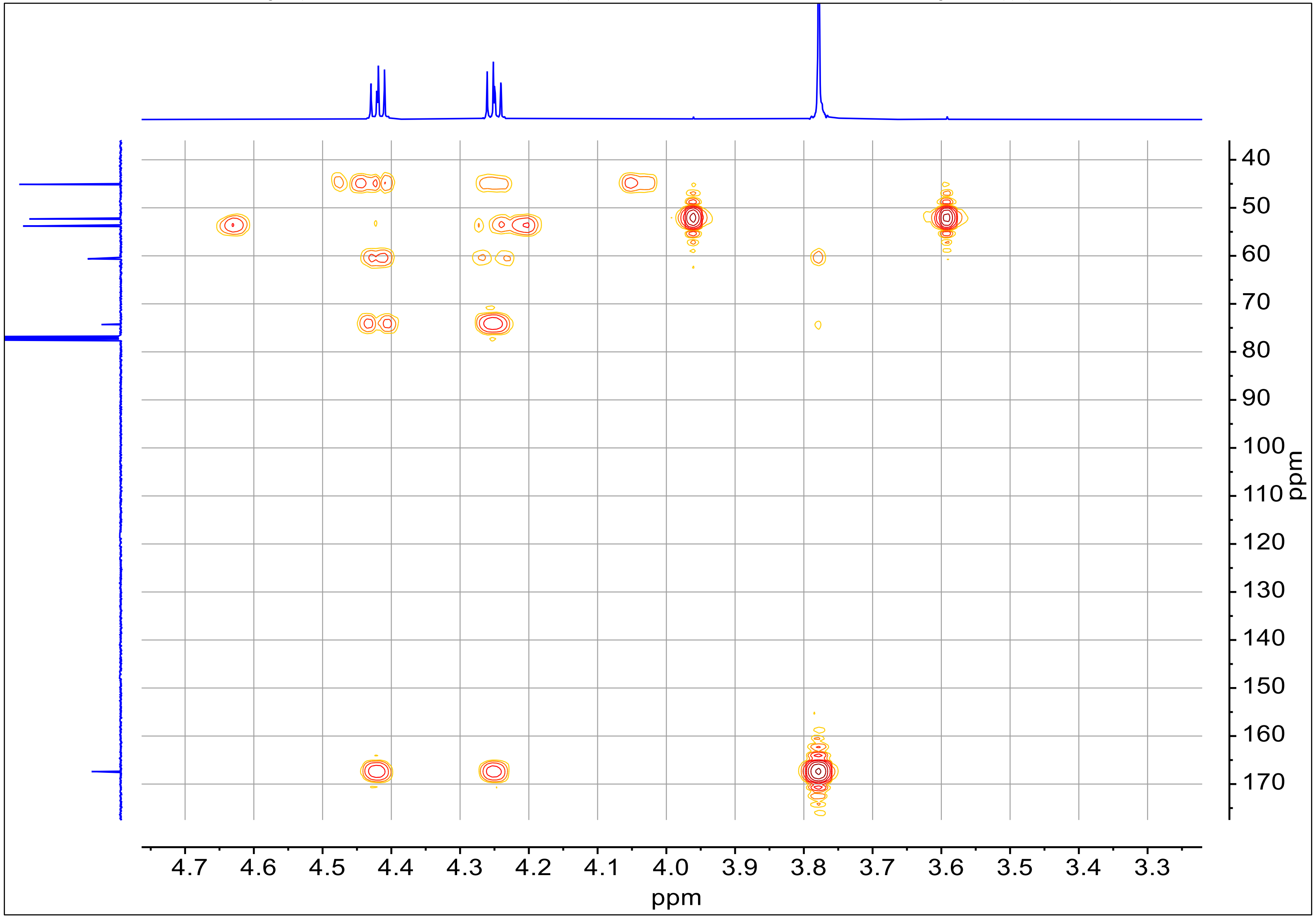

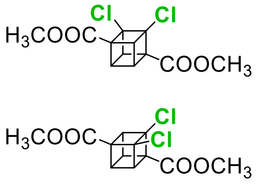

^{1}H NMR (400 MHz, $CDCl_3$): Dimethyl 2,7-Dichlorocubane-1,4-dicarboxylate (**2bMe**)

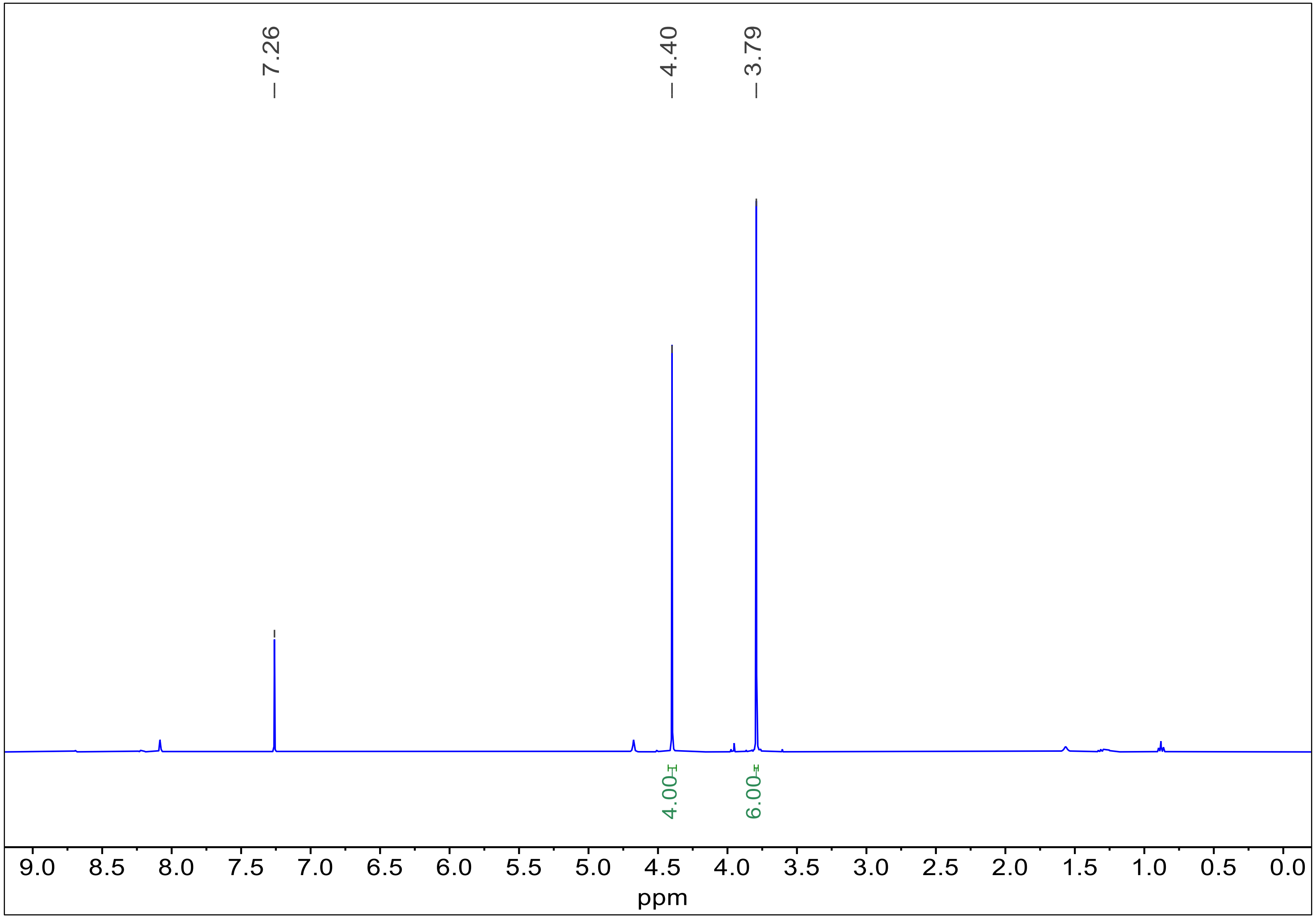

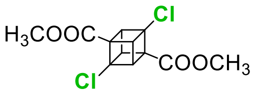

$^{13}C\ \{^{1}H\}$ NMR (100 MHz, $CDCl_3$): Dimethyl 2,7-Dichlorocubane-1,4-dicarboxylate (**2bMe**)

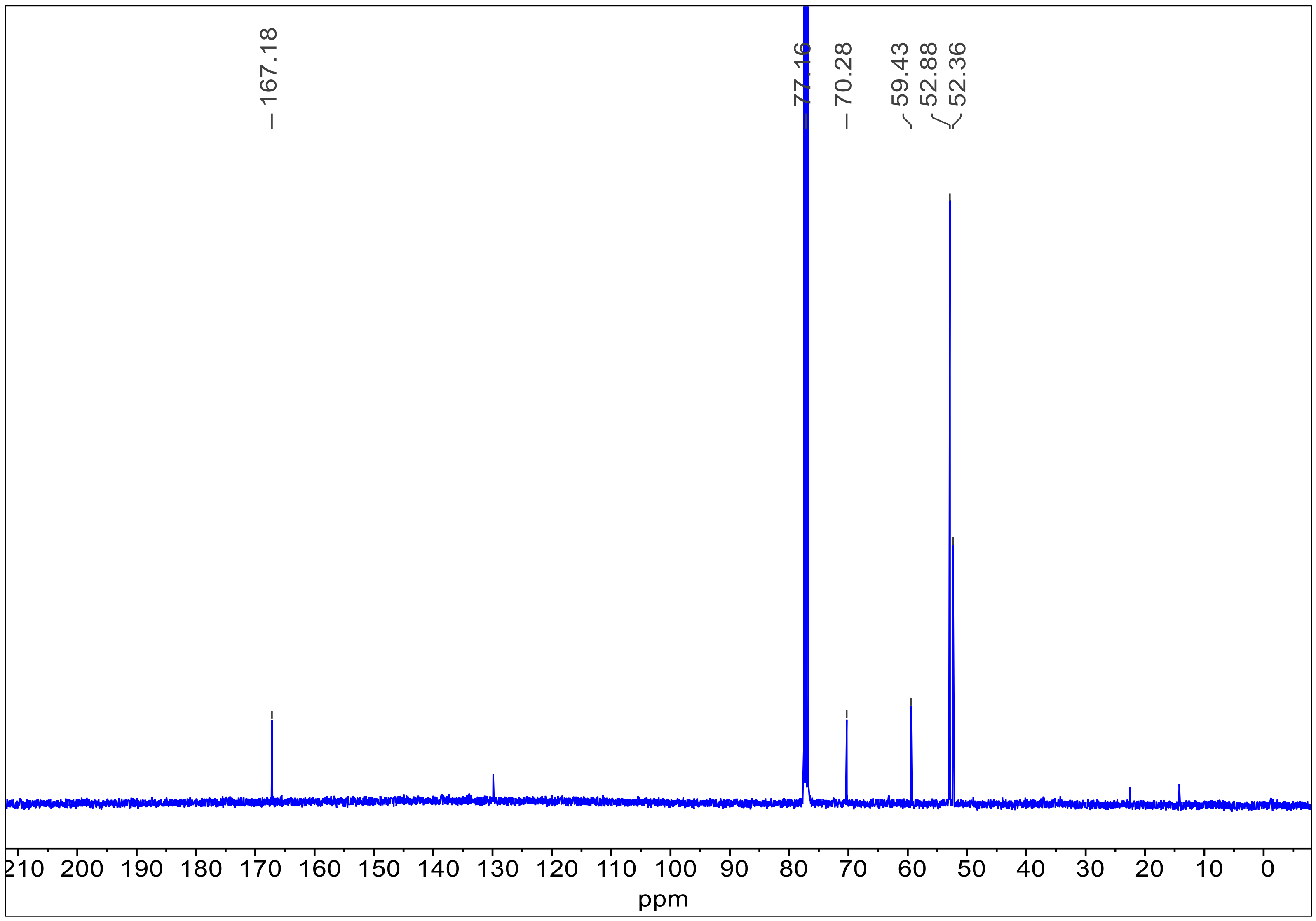

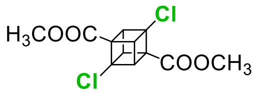

^{13}C APT NMR (100 MHz, $CDCl_3$): Dimethyl 2,7-Dichlorocubane-1,4-dicarboxylate (**2bMe**)

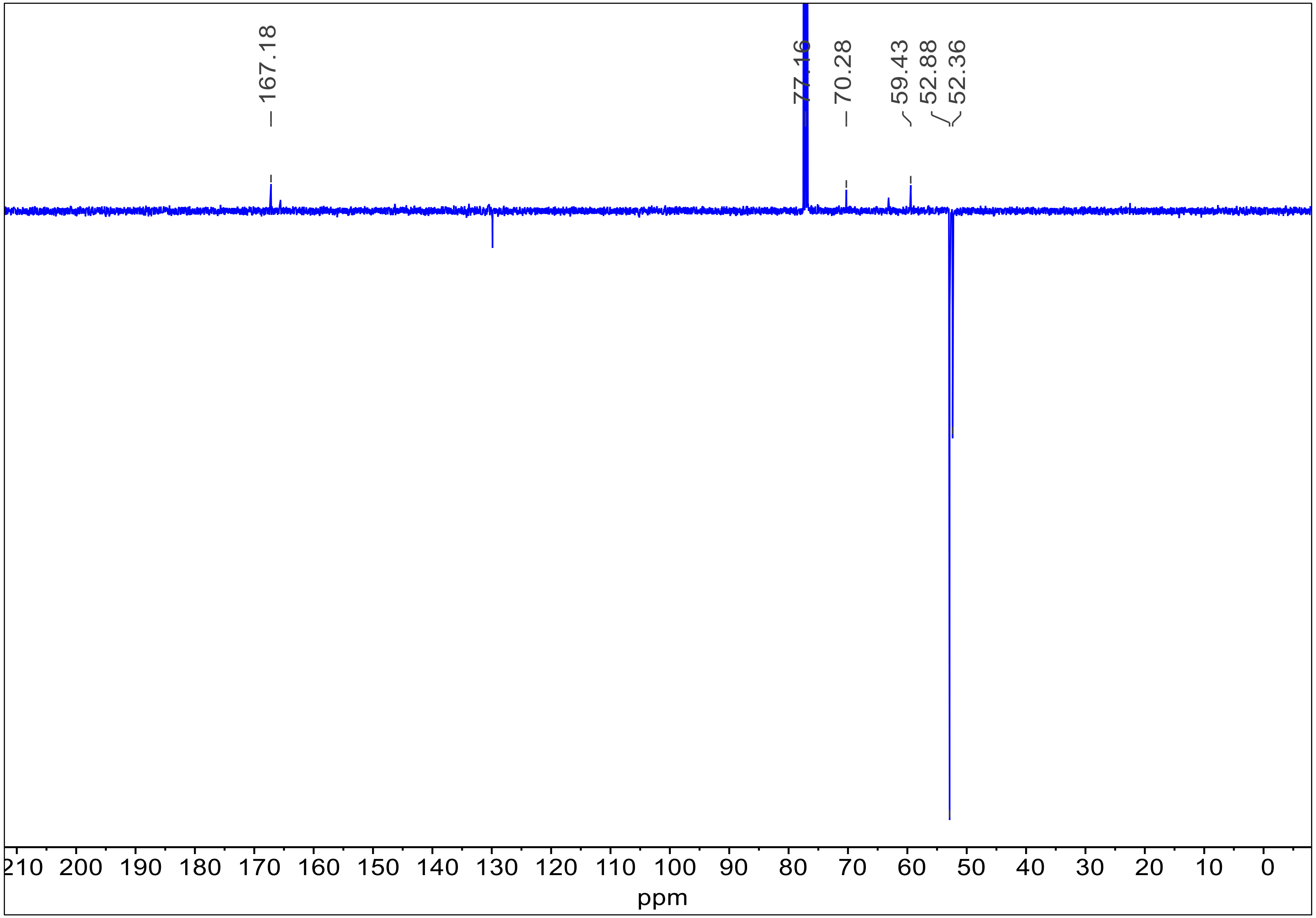

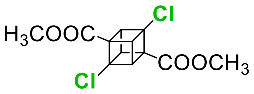

^{1}H – ^{1}H COSY ($CDCl_3$): Dimethyl 2,7-Dichlorocubane-1,4-dicarboxylate (**2bMe**)

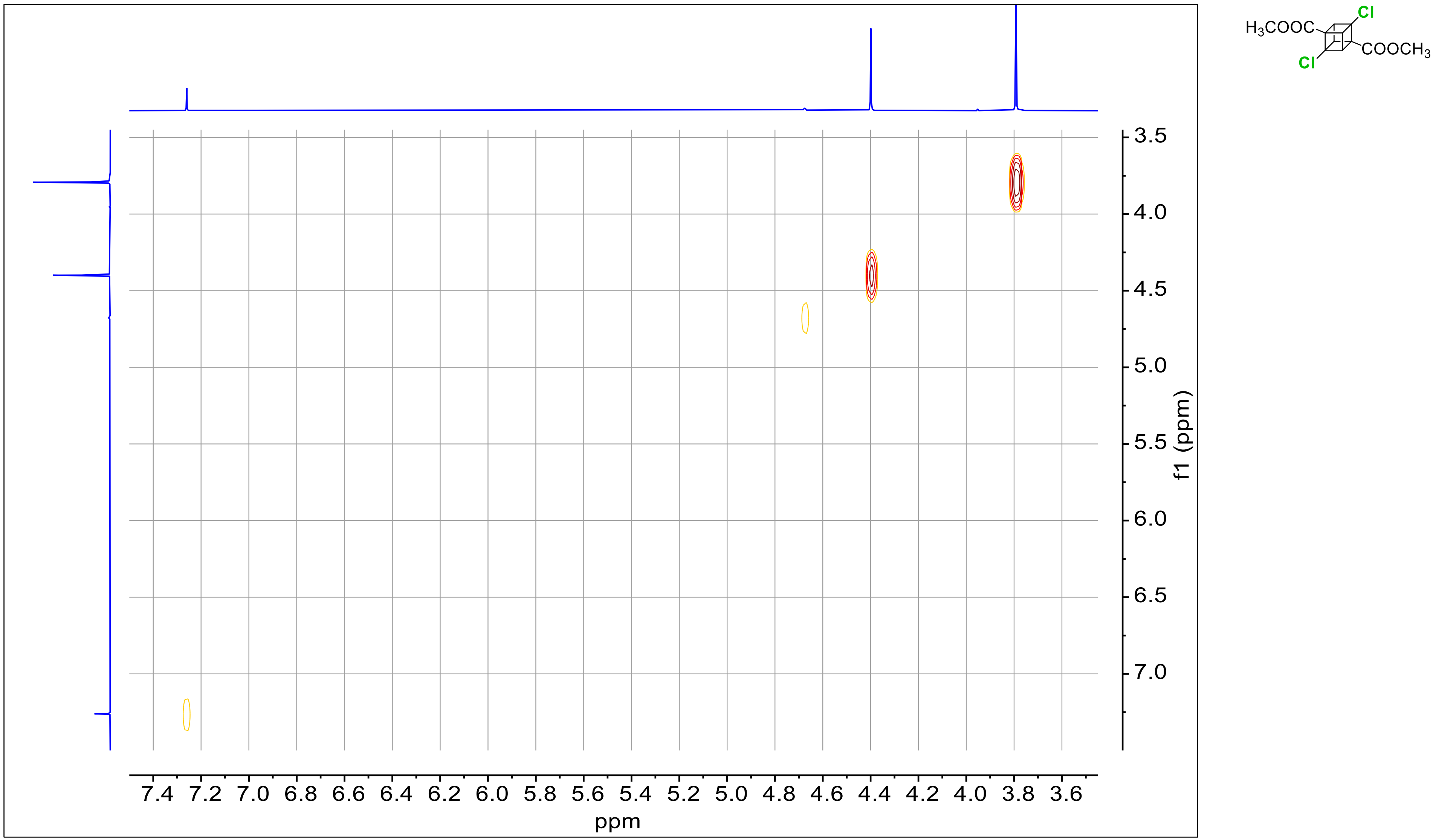

HSQC ($CDCl_3$): Dimethyl 2,7-Dichlorocubane-1,4-dicarboxylate (**2bMe**)

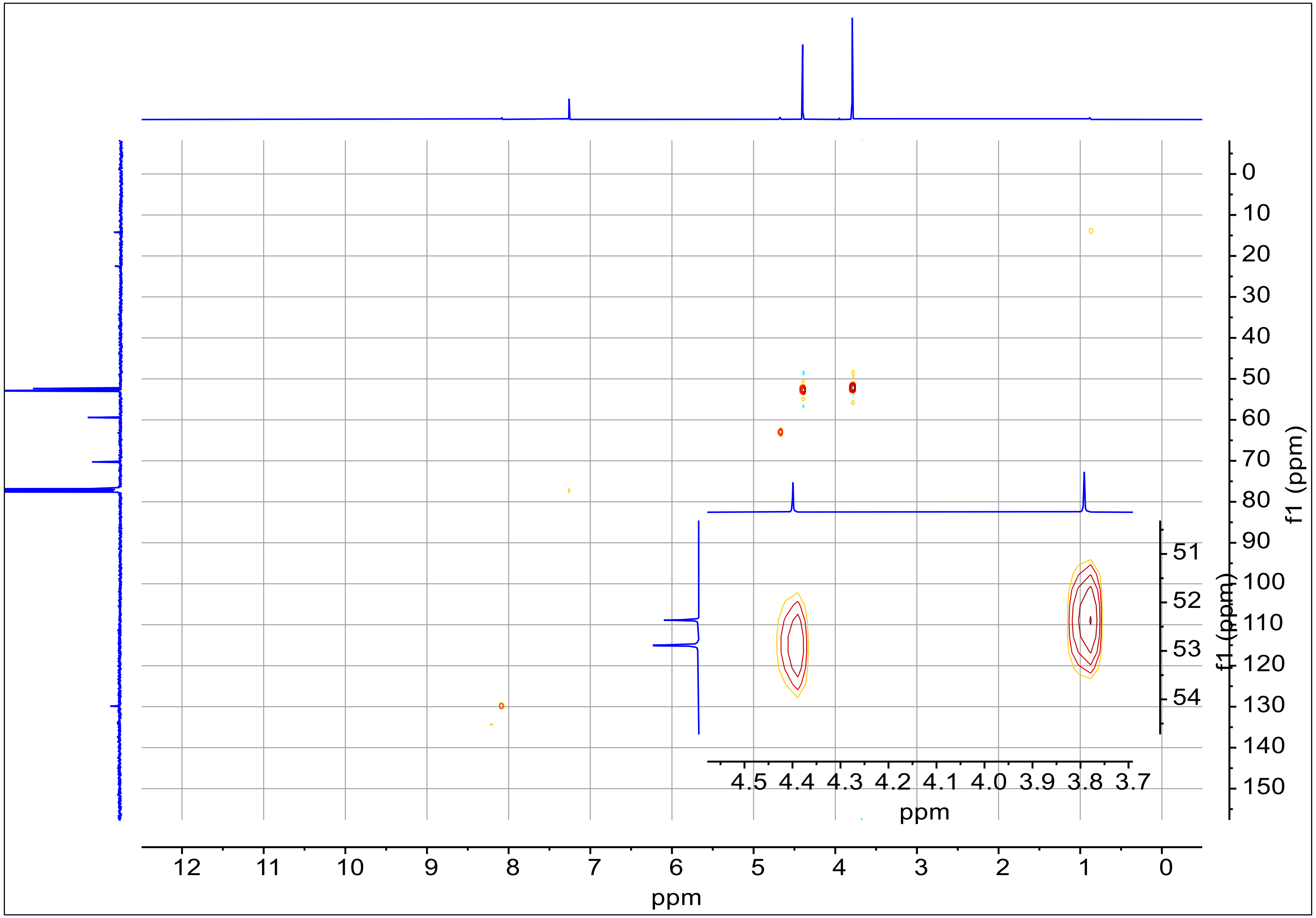

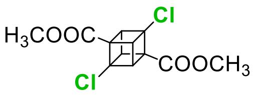

HMBC ($CDCl_3$): Dimethyl 2,7-Dichlorocubane-1,4-dicarboxylate (**2bMe**)

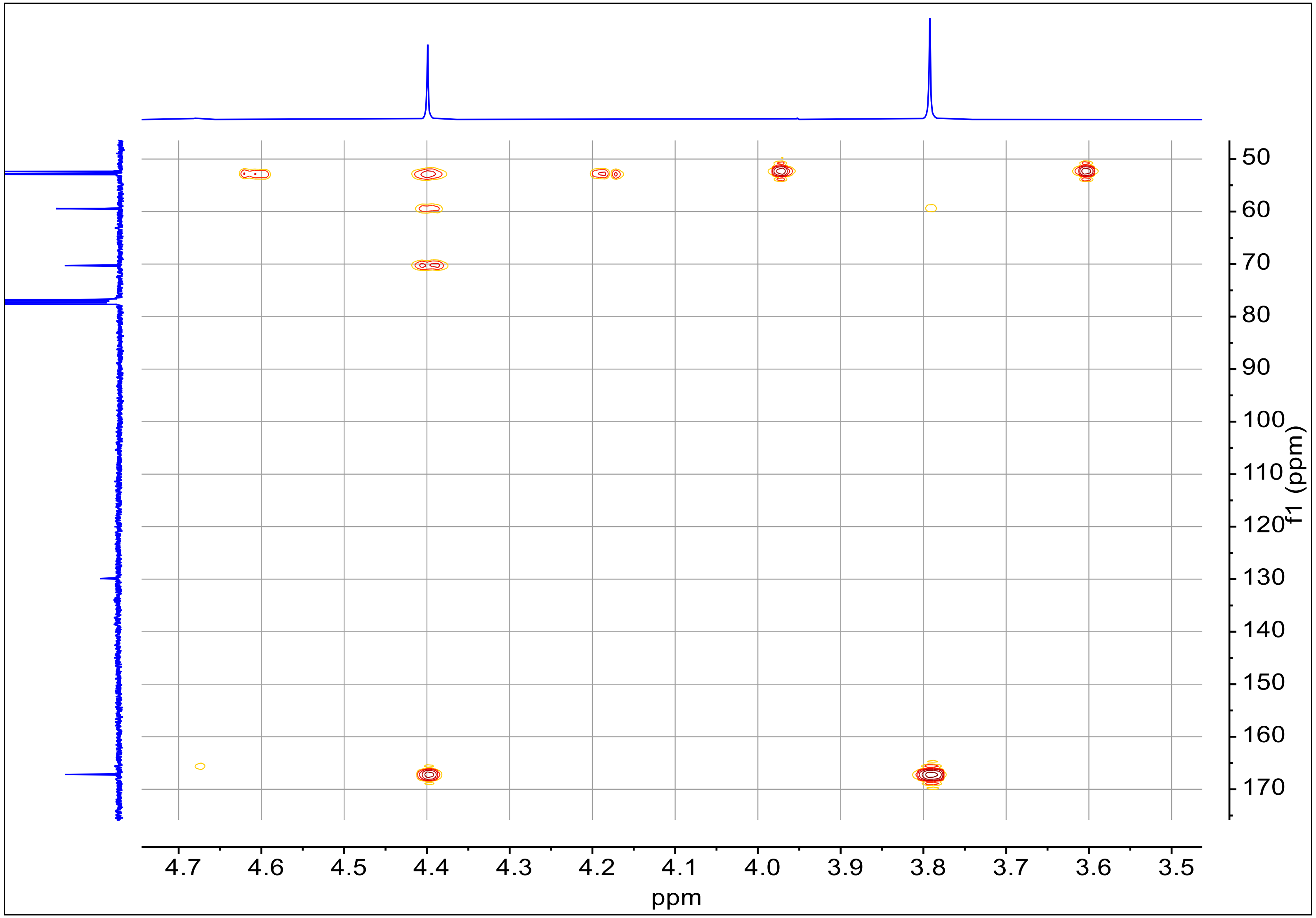

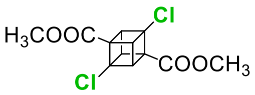

^{1}H NMR (400 MHz, $CDCl_3$): Dimethyl 2,6-Dichlorocubane-1,4-dicarboxylate (**2cMe**)

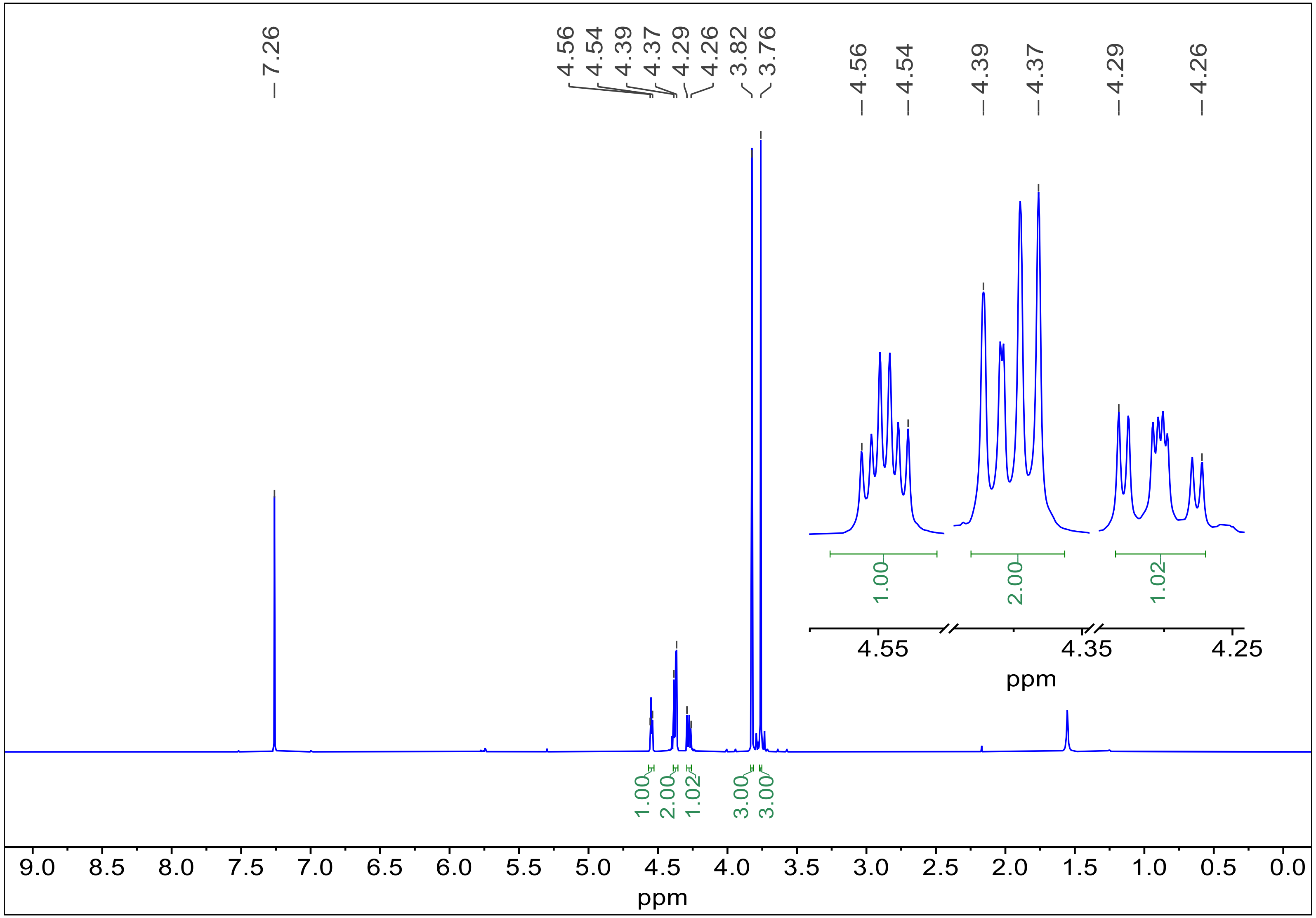

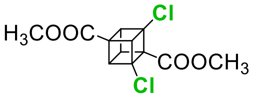

^{13}C {^1H} NMR (100 MHz, $CDCl_3$): Dimethyl 2,6-Dichlorocubane-1,4-dicarboxylate (**2cMe**)

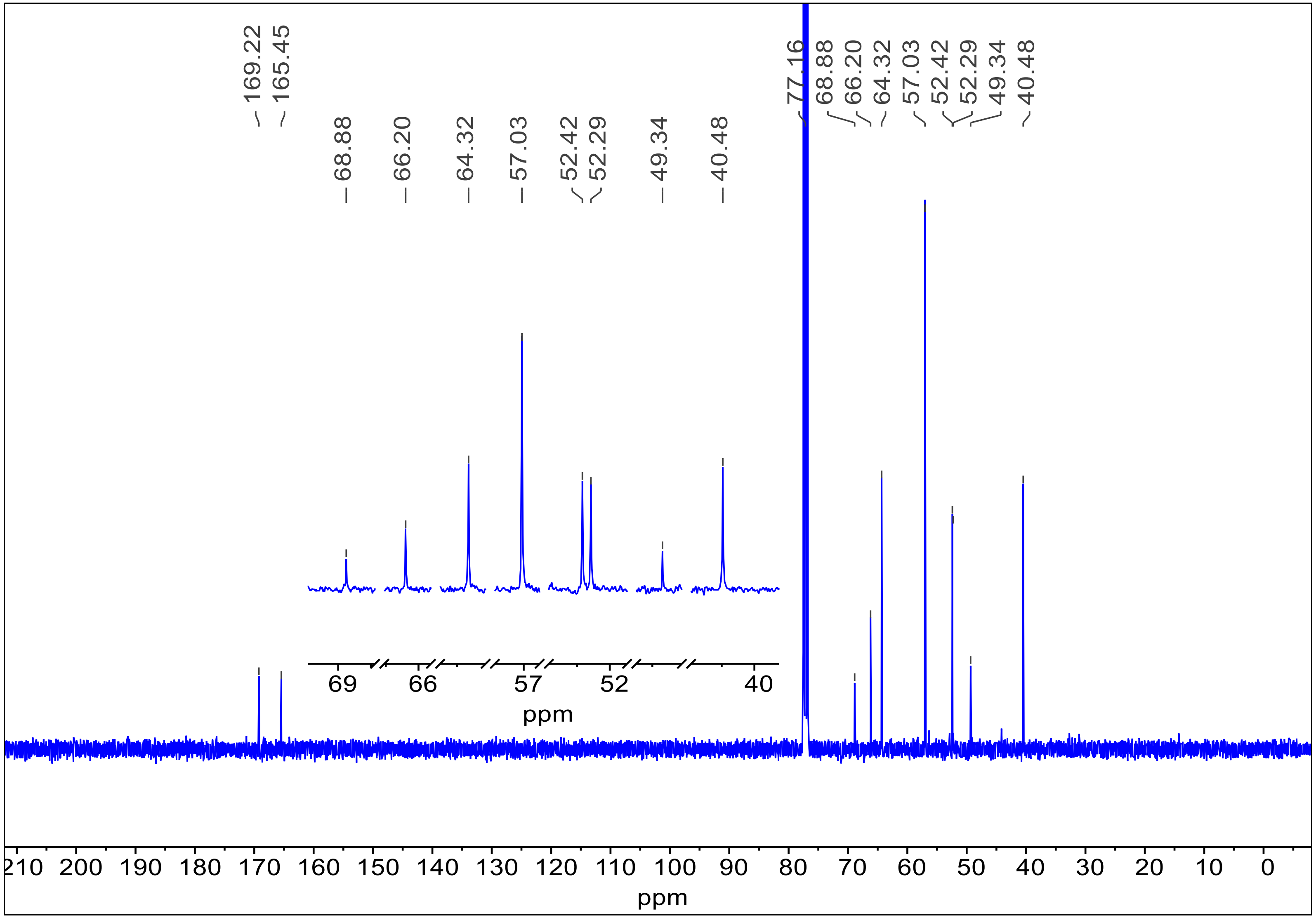

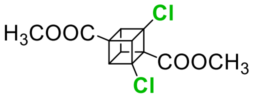

^{13}C APT NMR (100 MHz, $CDCl_3$): Dimethyl 2,6-Dichlorocubane-1,4-dicarboxylate (**2cMe**)

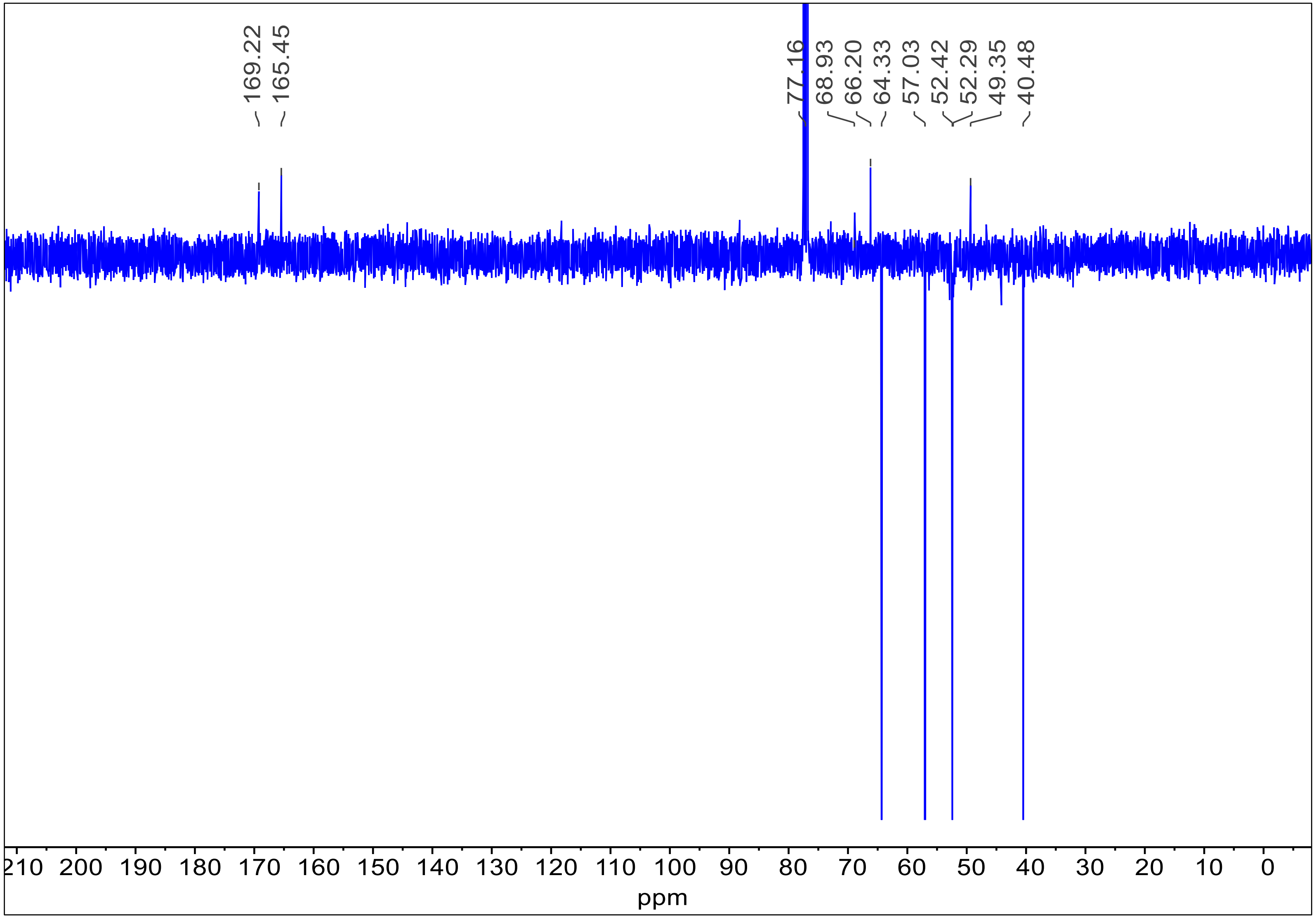

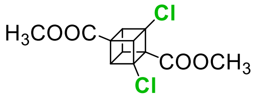

^{1}H – ^{1}H COSY ($CDCl_3$): Dimethyl 2,6-Dichlorocubane-1,4-dicarboxylate (**2cMe**)

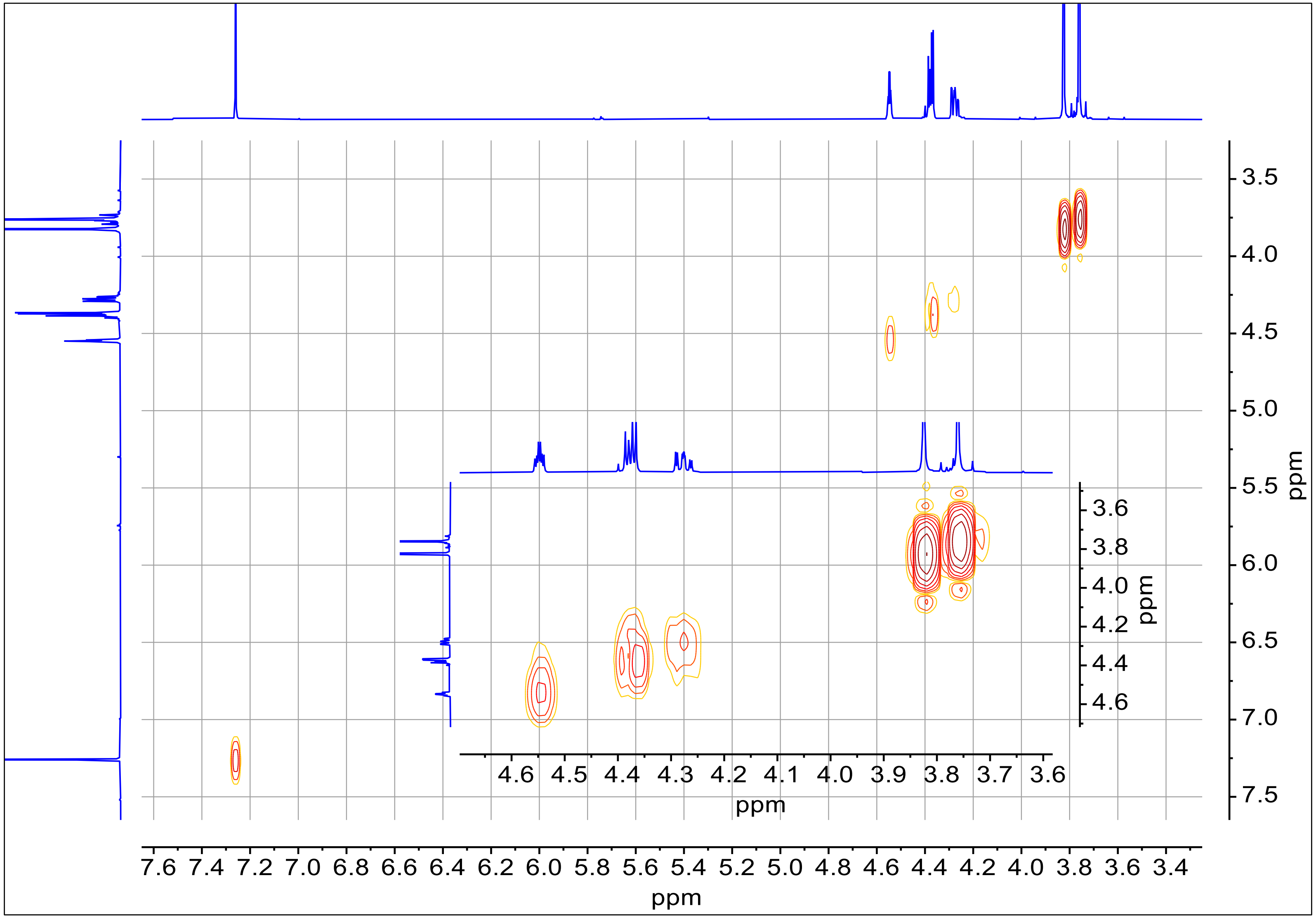

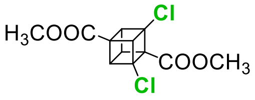

HSQC ($CDCl_3$): Dimethyl 2,6-Dichlorocubane-1,4-dicarboxylate (**2cMe**)

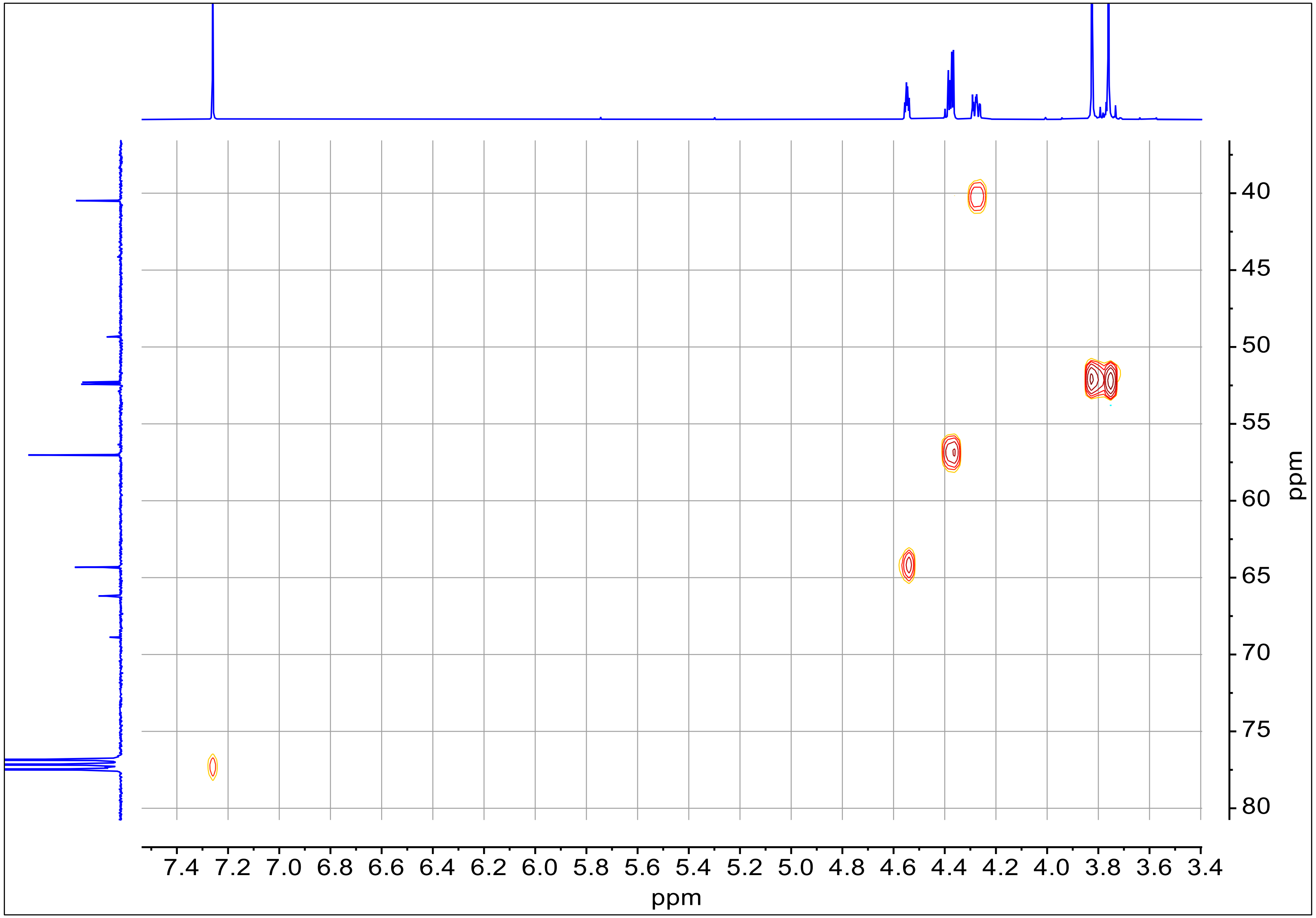

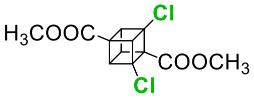

HMBC ($CDCl_3$): Dimethyl 2,6-Dichlorocubane-1,4-dicarboxylate (**2cMe**)

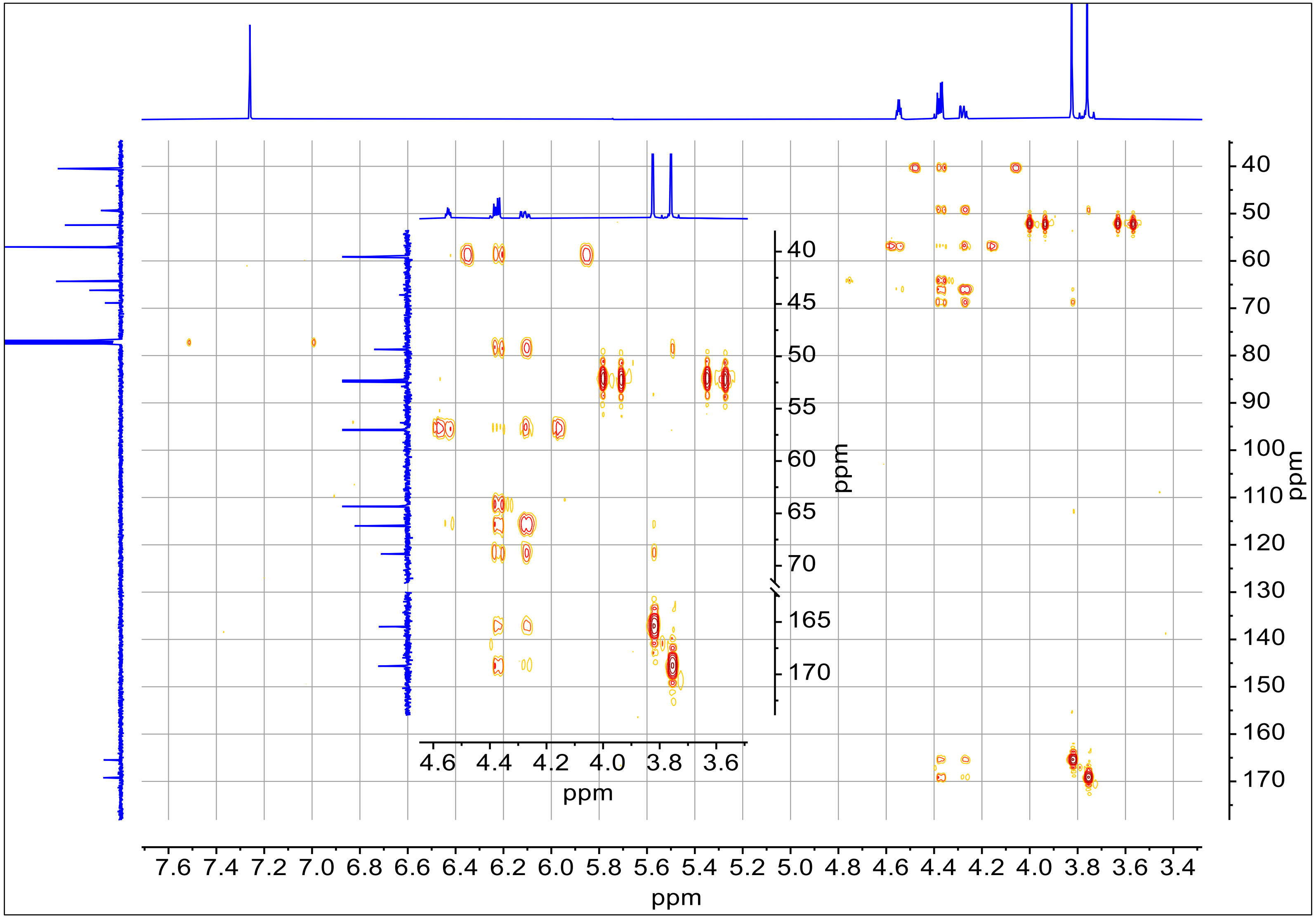

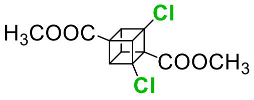

^{1}H NMR (400 MHz, DMSO-d_6): Cubane-1,4-dicarboxylic acid (**0**)

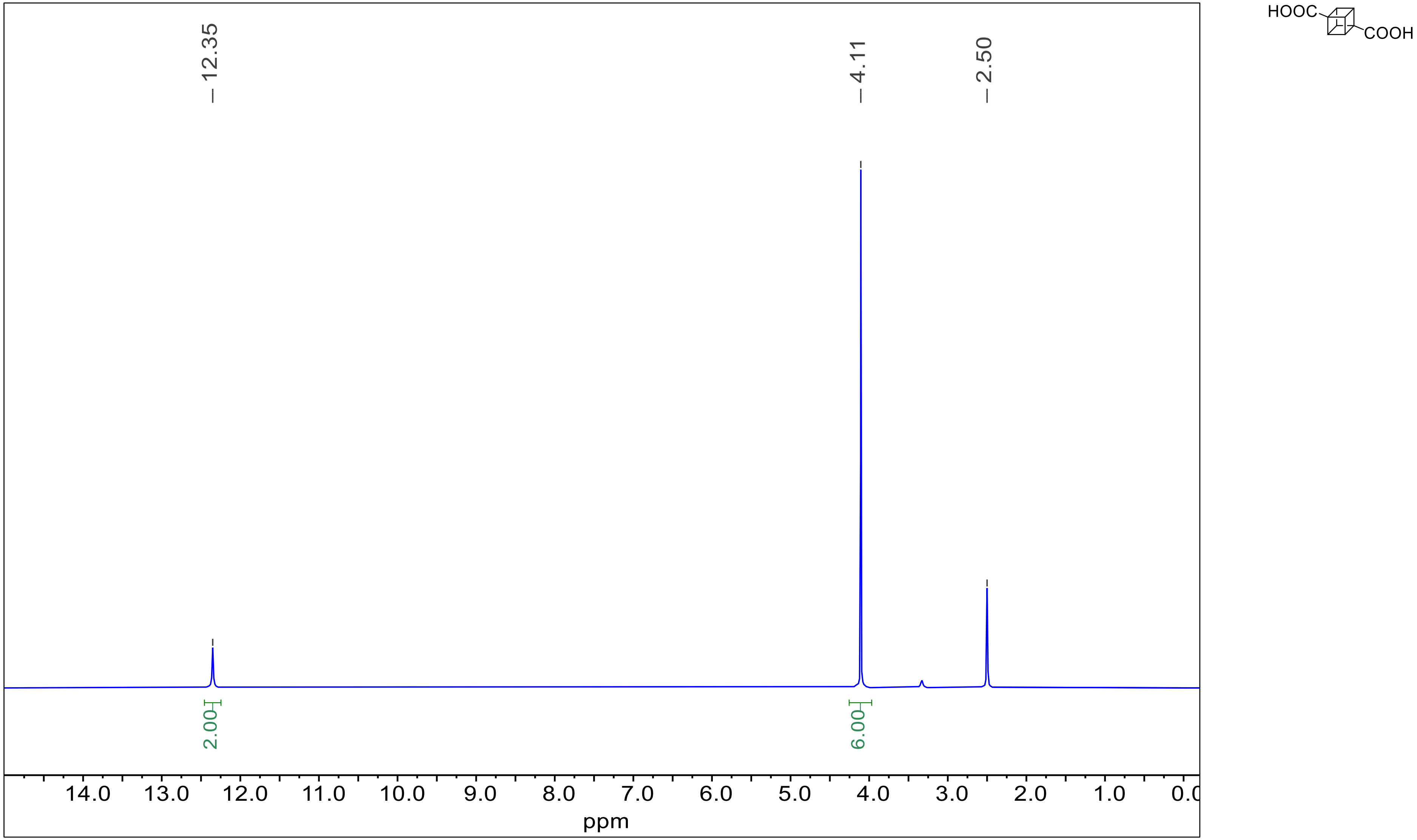

^{13}C {^{1}H} NMR (100 MHz, DMSO-d_6): Cubane-1,4-dicarboxylic acid (**0**)

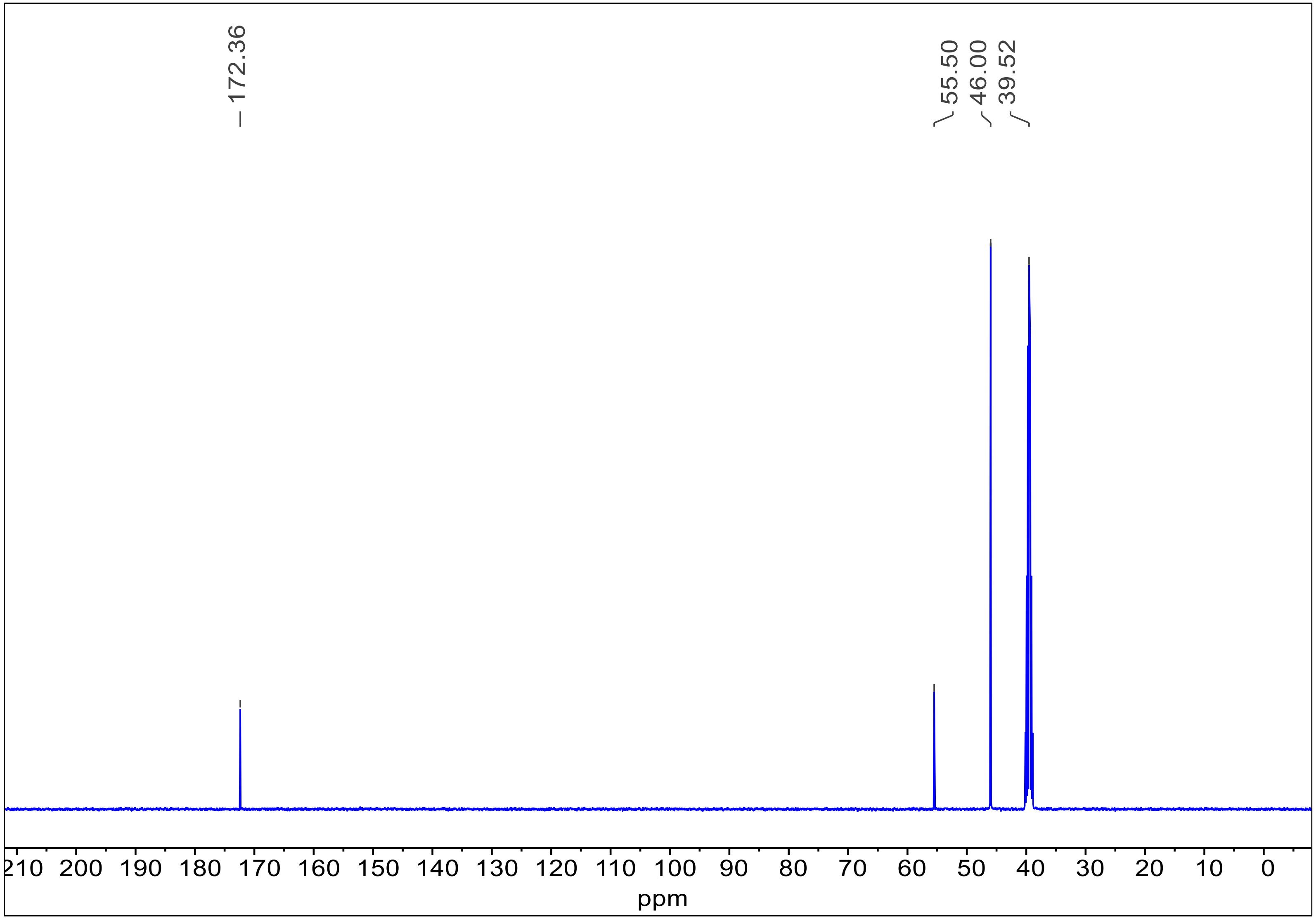

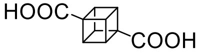

^{13}C APT NMR (100 MHz, DMSO-d_6): Cubane-1,4-dicarboxylic acid (**0**)

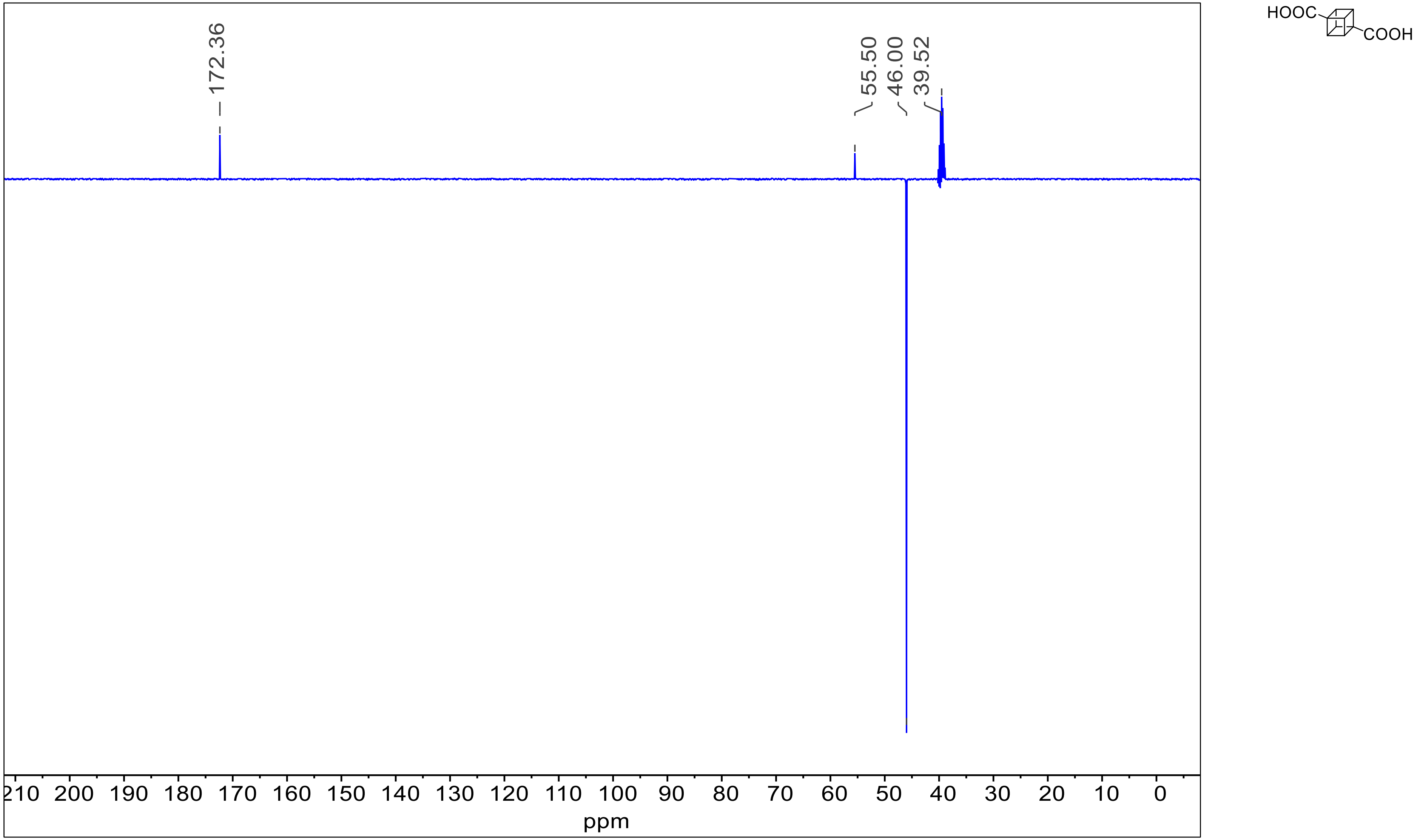

^1H – ^1H COSY (DMSO-d_6): Cubane-1,4-dicarboxylic acid (**0**)

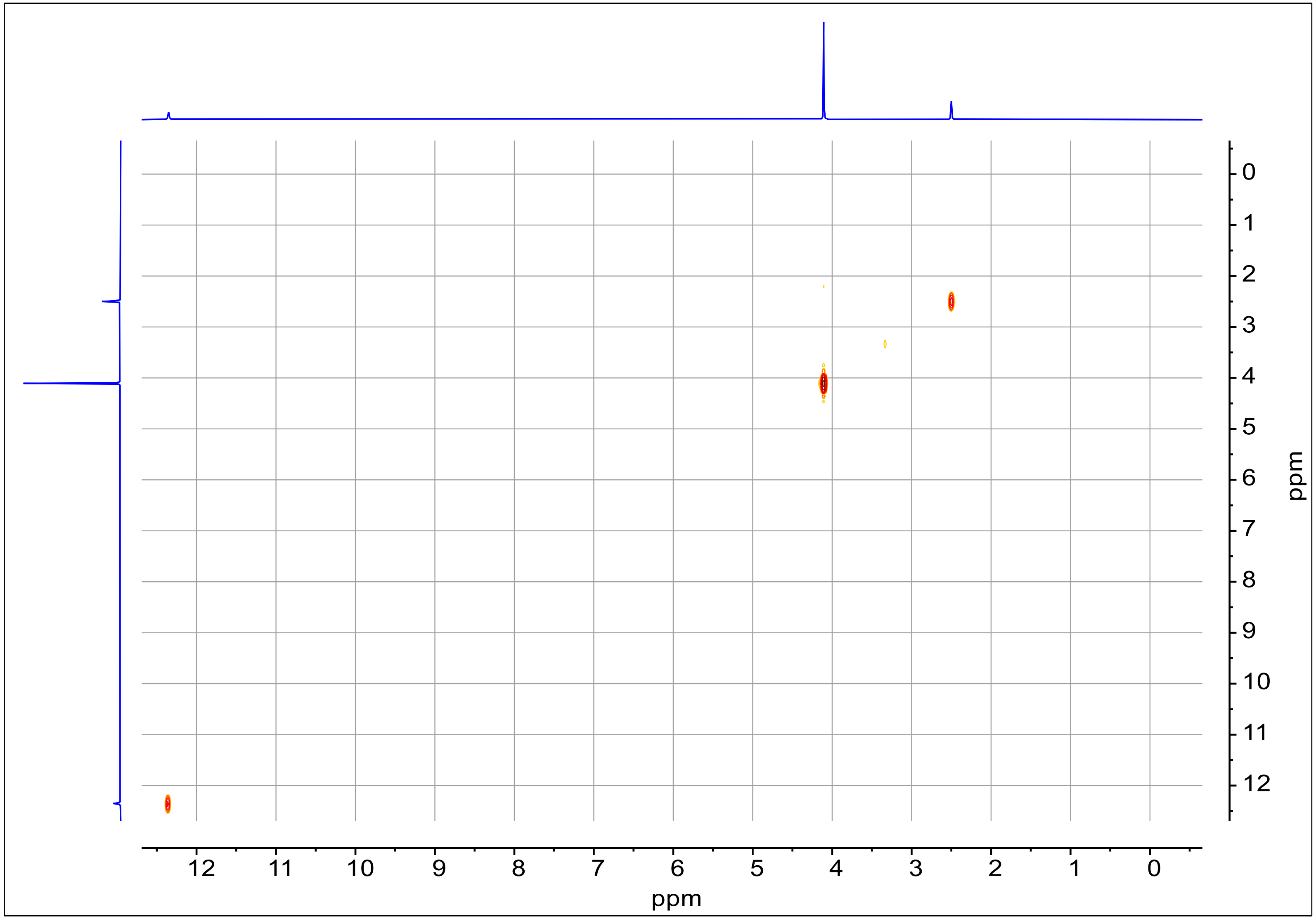

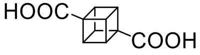

HSQC (DMSO-d_6): Cubane-1,4-dicarboxylic acid (**0**)

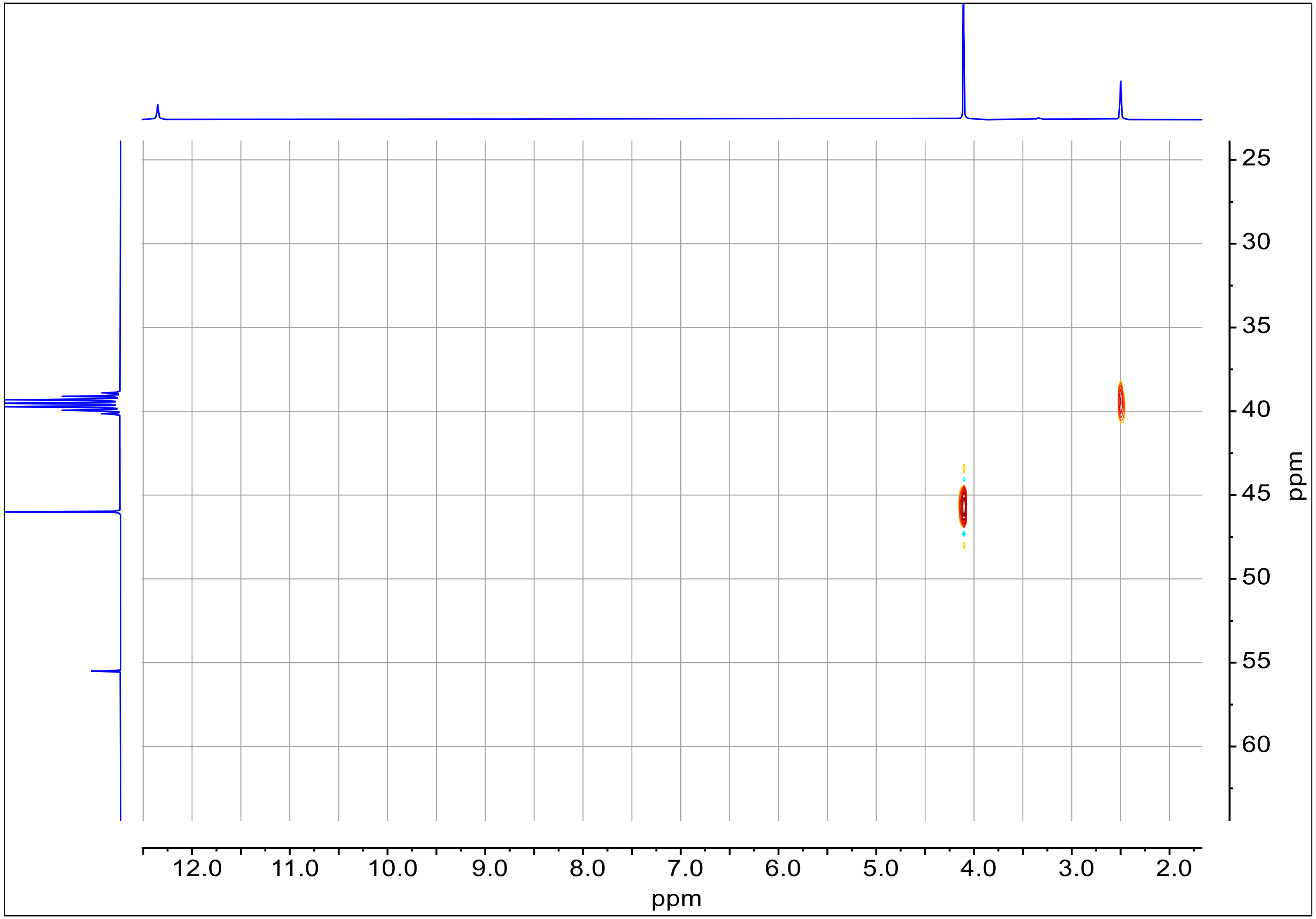

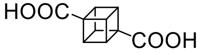

HMBC (DMSO-d_6): Cubane-1,4-dicarboxylic acid (**0**)

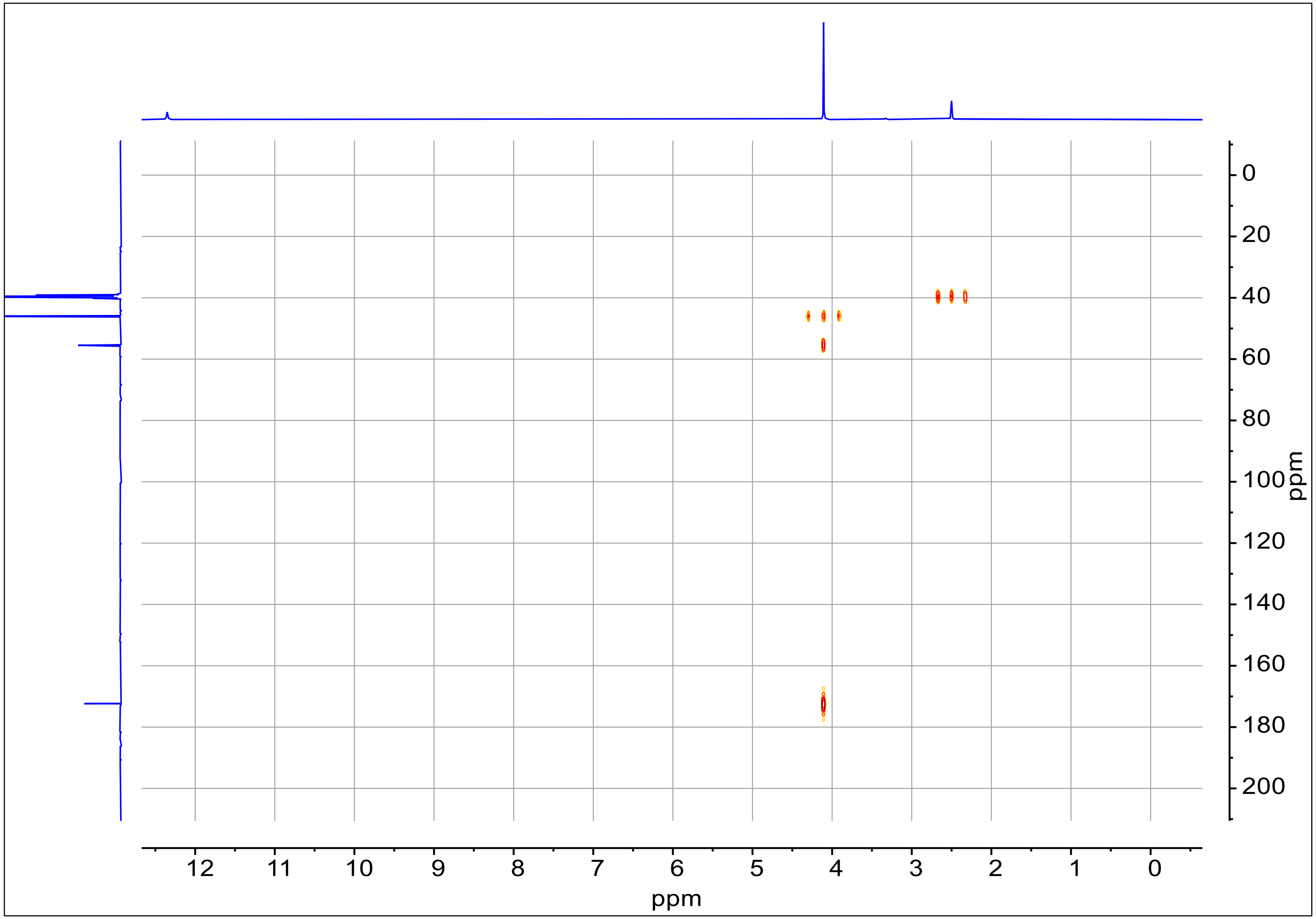

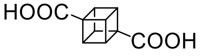

^{1}H NMR (400 MHz, acetone-d_6): 2-Chlorocubane-1,4-dicarboxylic acid (**1**)

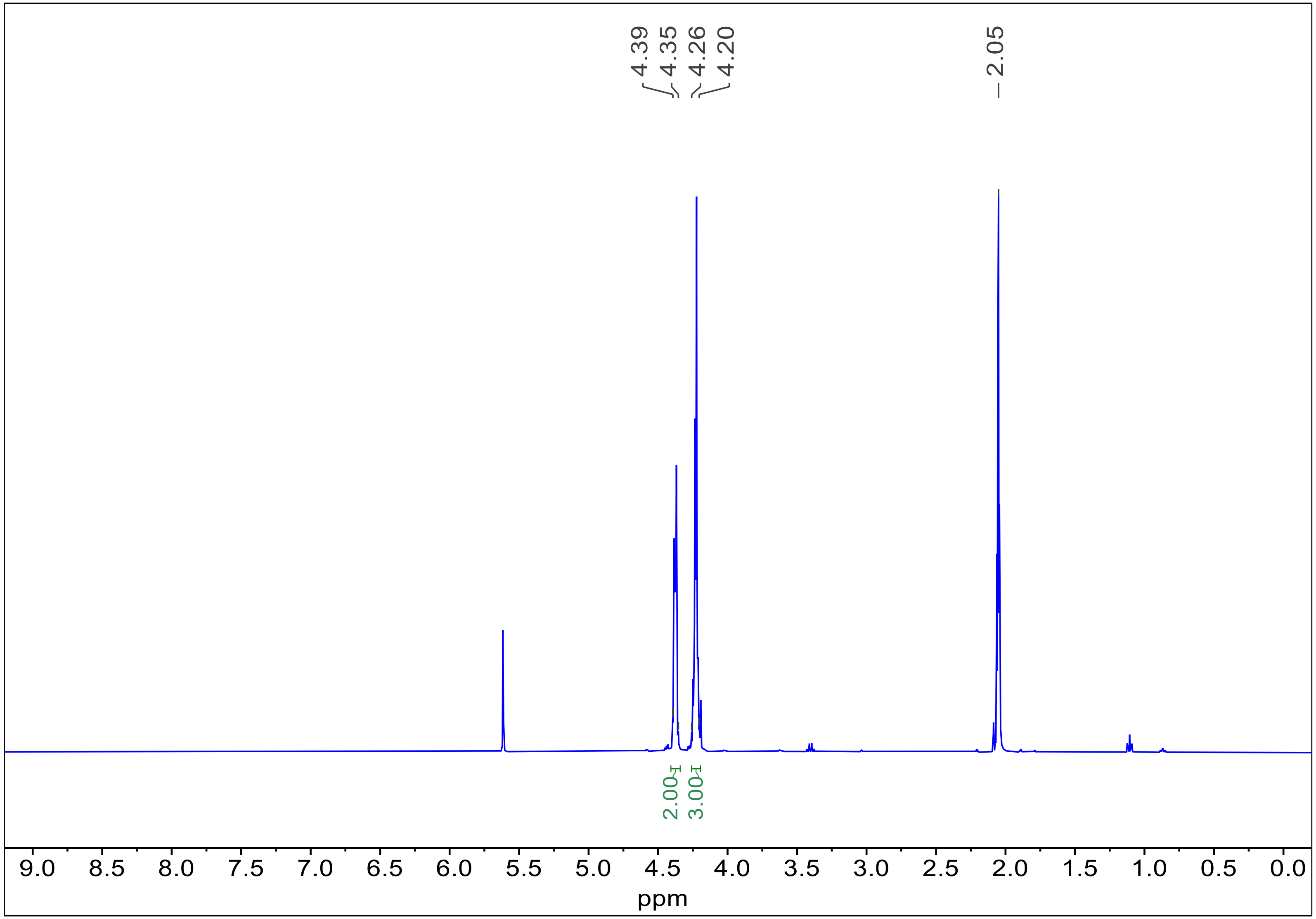

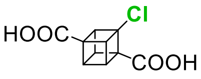

^{13}C {^{1}H} NMR (100 MHz, acetone-d_6): 2-Chlorocubane-1,4-dicarboxylic acid (**1**)

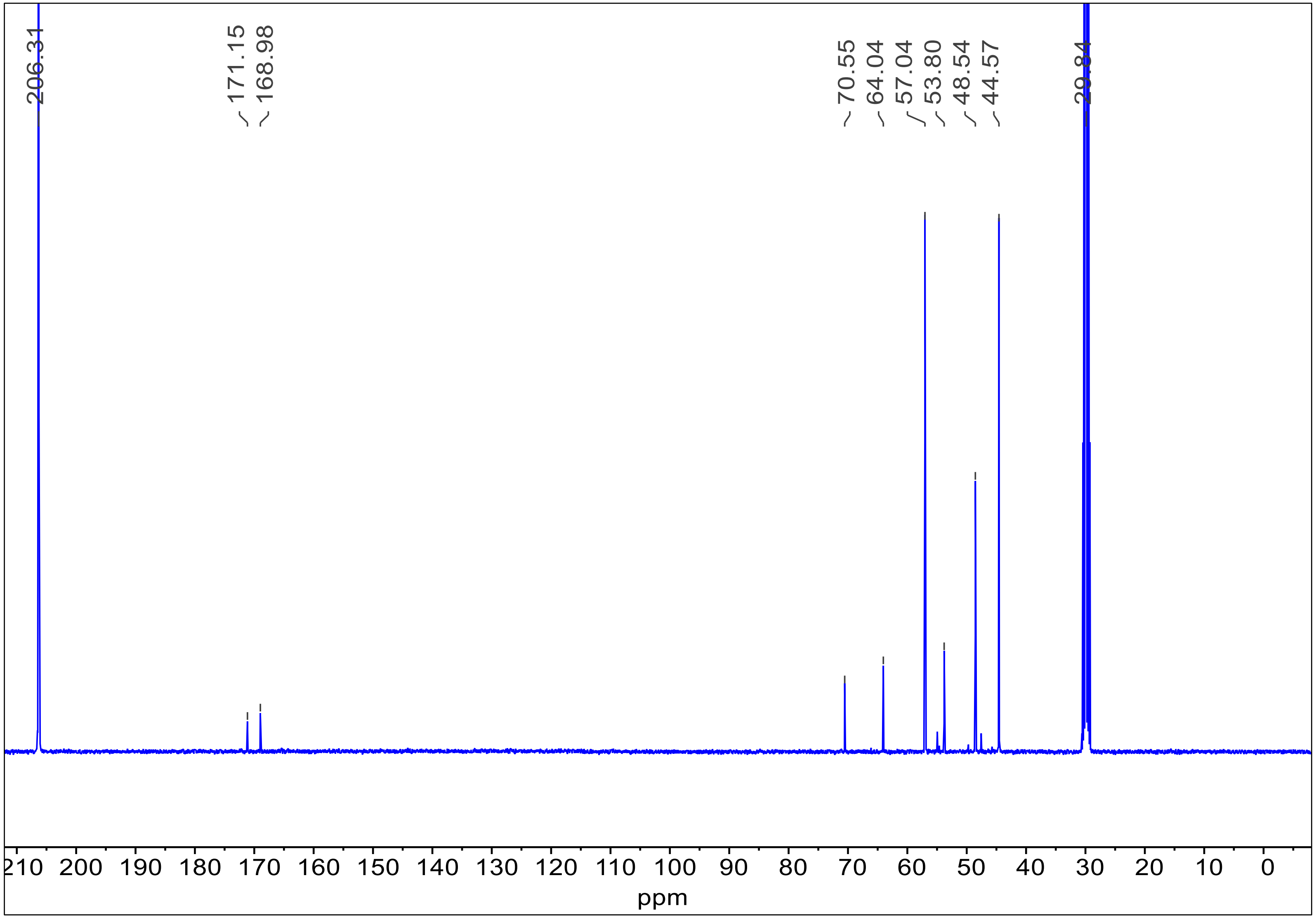

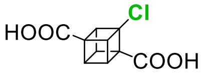

^{13}C APT NMR (100 MHz, acetone-d_6): 2-Chlorocubane-1,4-dicarboxylic acid (**1**)

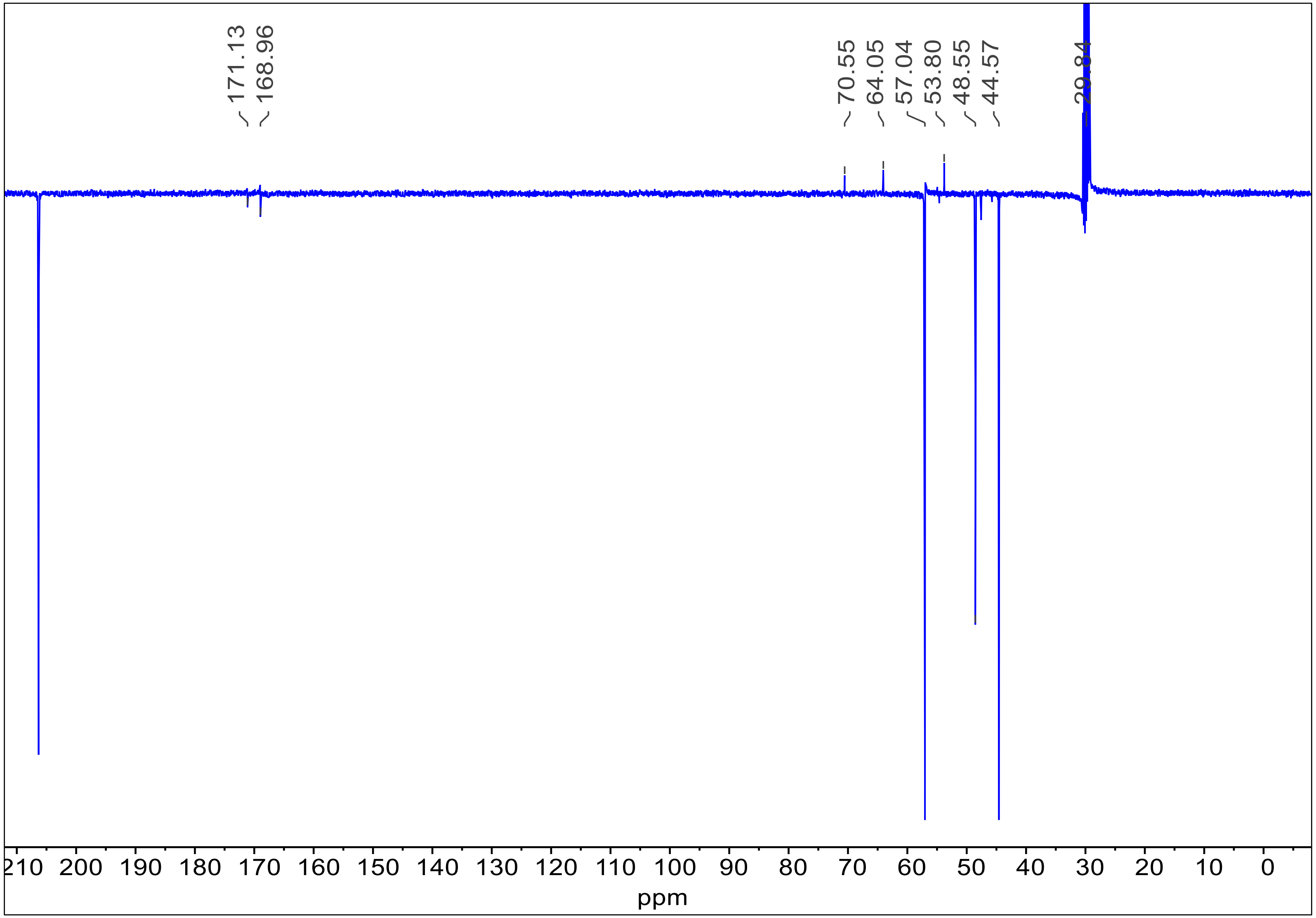

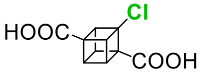

^{1}H – ^{1}H COSY (acetone-d_6): 2-Chlorocubane-1,4-dicarboxylic acid (**1**)

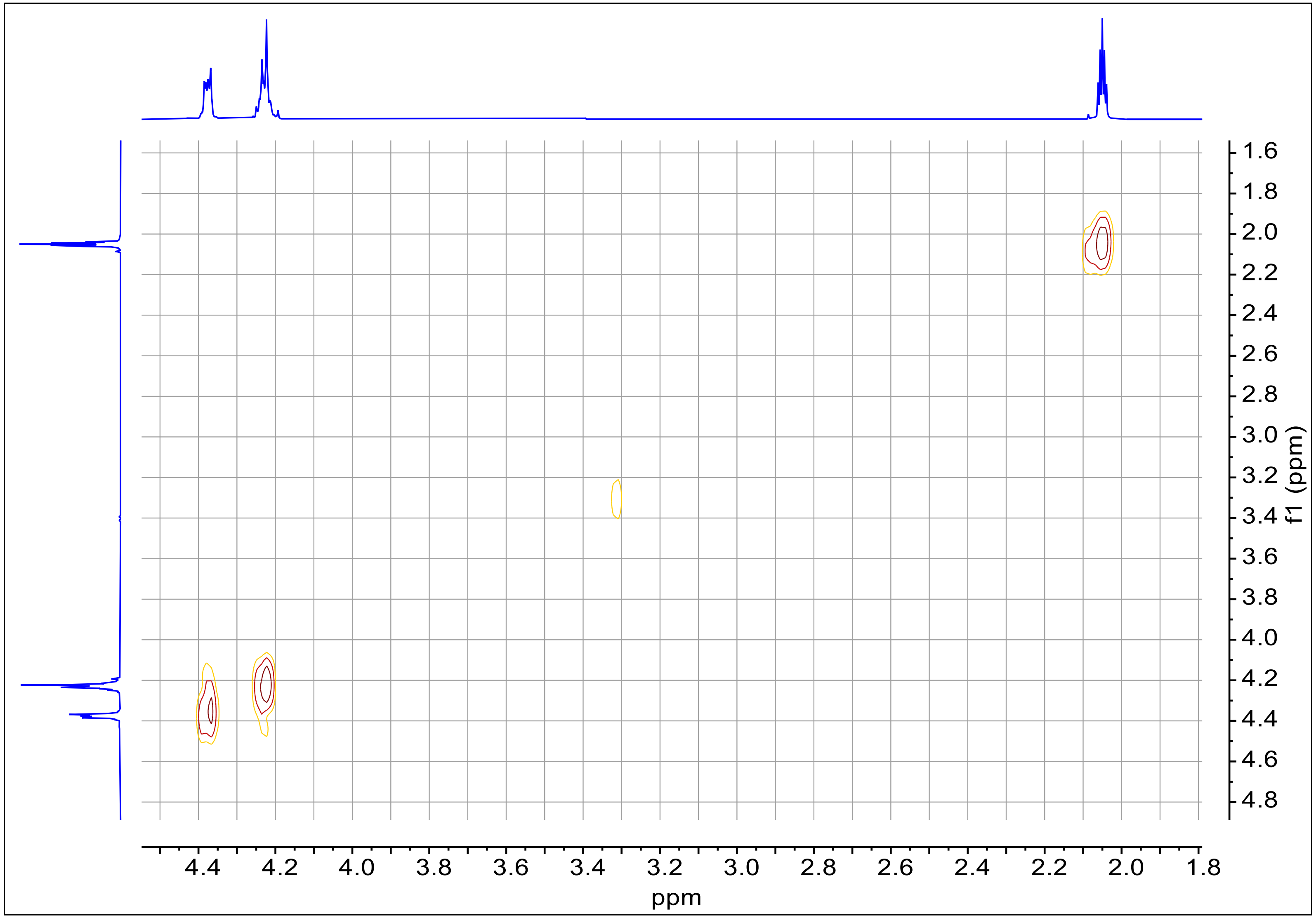

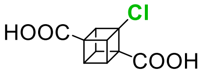

HSQC (acetone-d_6): 2-Chlorocubane-1,4-dicarboxylic acid (**1**)

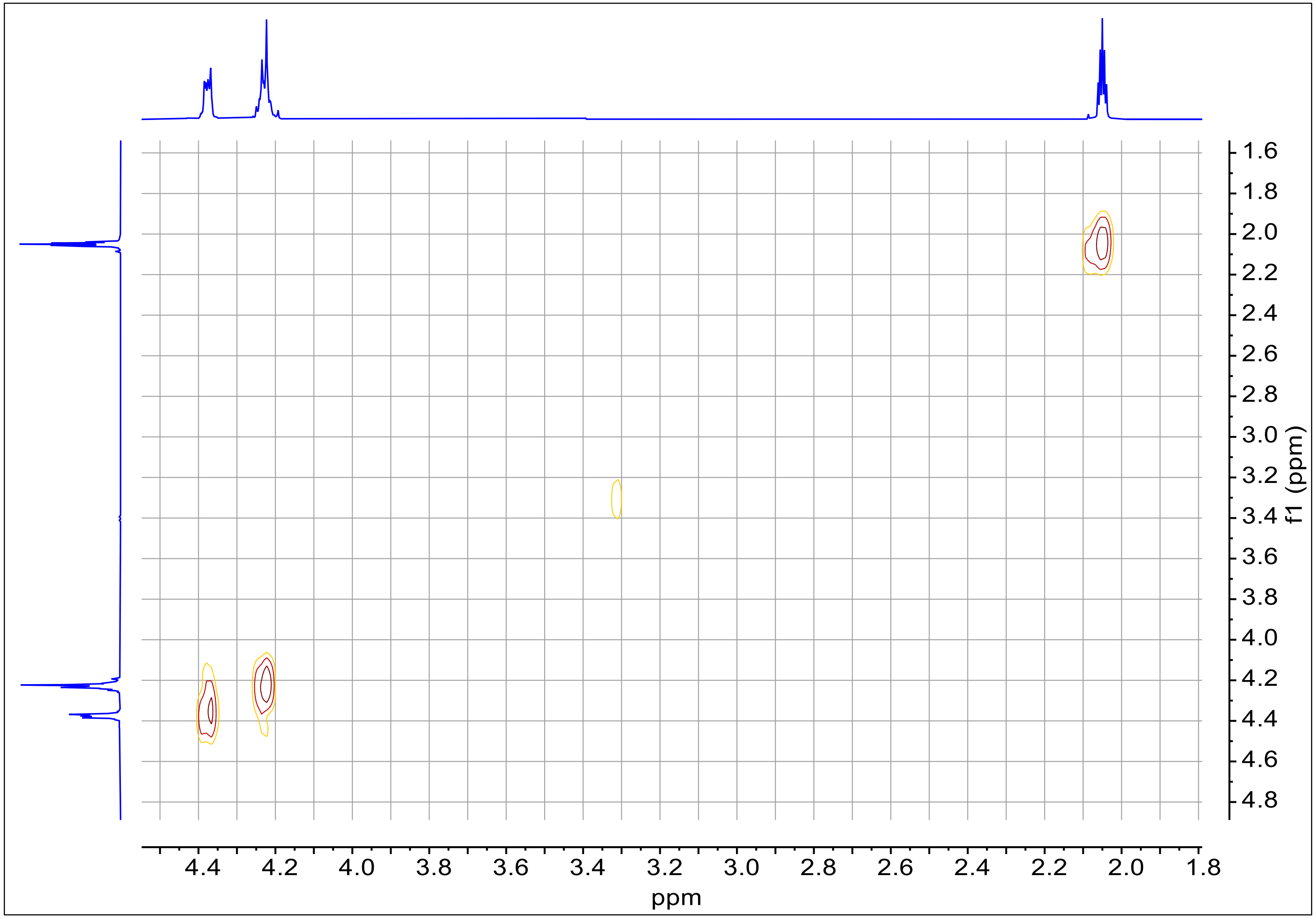

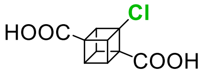

HMBC (acetone-d_6): 2-Chlorocubane-1,4-dicarboxylic acid (**1**)

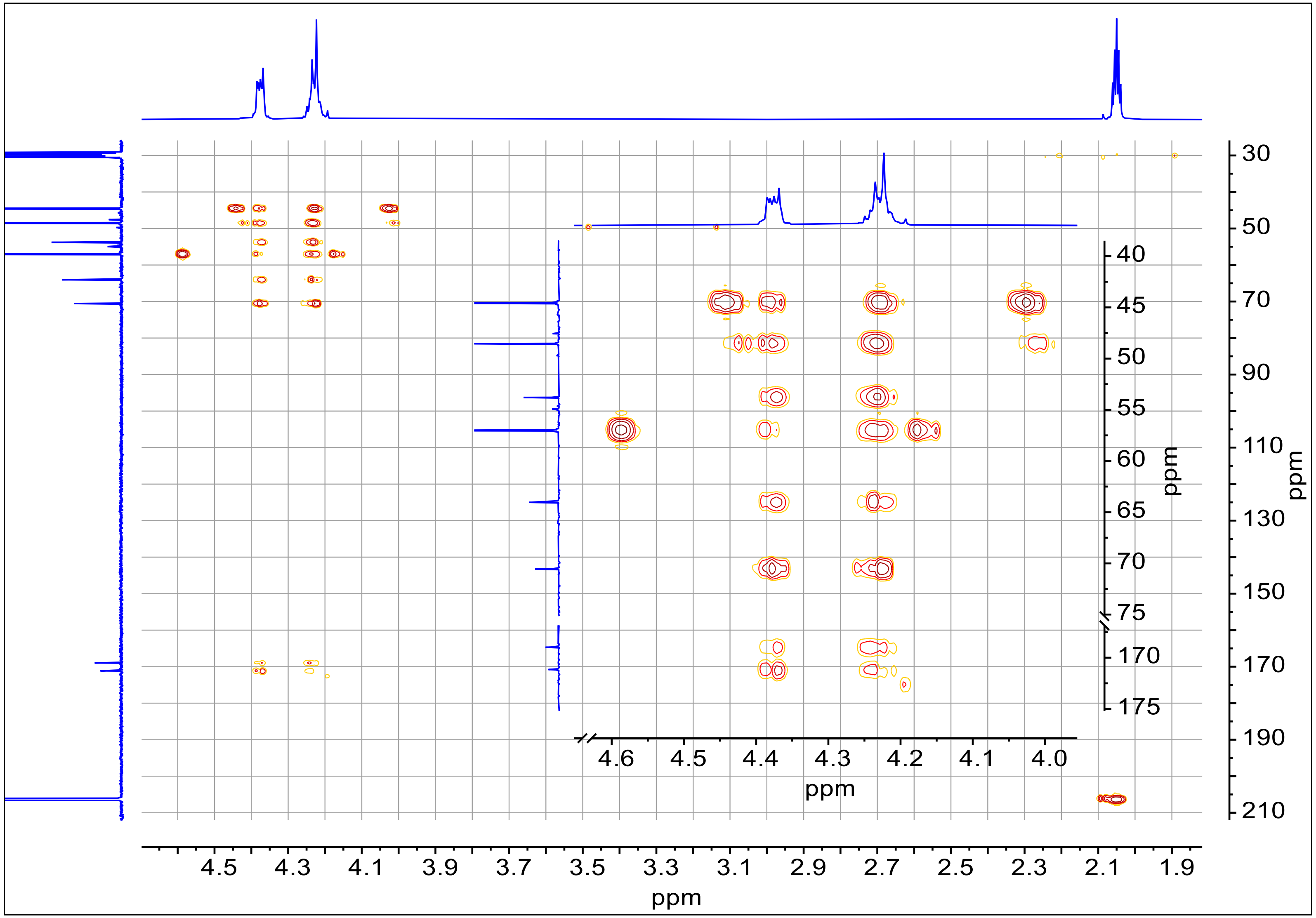

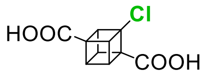

^{1}H NMR (400 MHz, acetone-d_6): (1*S*,2*R*,3*R*,4*S*,5*R*,6*R*,7*R*,8*R*)-2,3-dichlorocubane-1,4-dicarboxylic acid (**(-)-2a**) and (1*R*,2*S*,3*S*,4*R*,5*S*,6*S*,7*S*,8*S*)-2,3-dichlorocubane-1,4-dicarboxylic acid (**(+)-2a**)

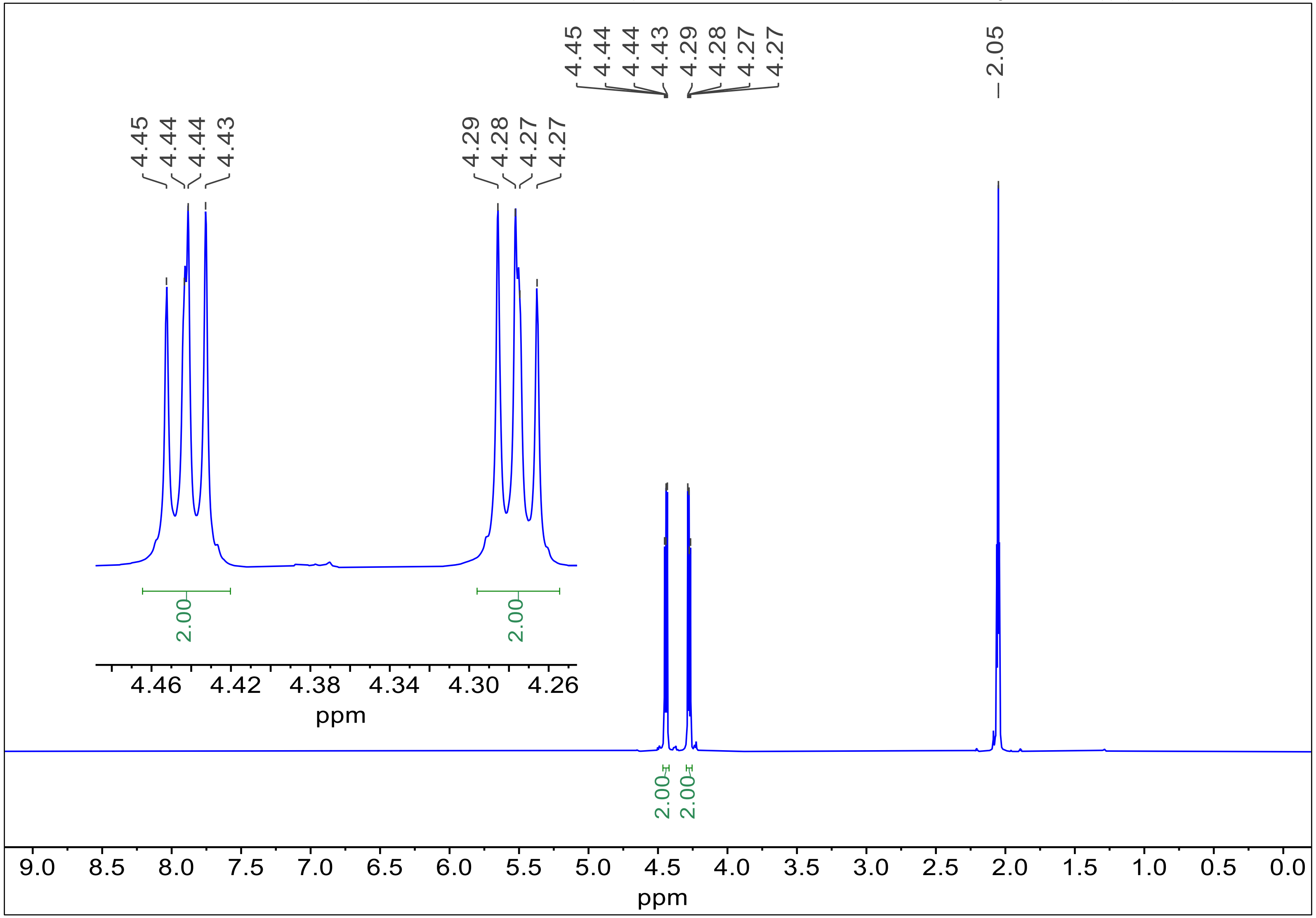

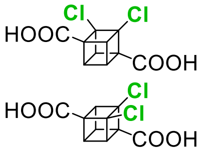

^{13}C {^{1}H} NMR (100 MHz, acetone-d_6): (1*S*,2*R*,3*R*,4*S*,5*R*,6*R*,7*R*,8*R*)-2,3-dichlorocubane-1,4-dicarboxylic acid (**(-)-2a**) and (1*R*,2*S*,3*S*,4*R*,5*S*,6*S*,7*S*,8*S*)-2,3-dichlorocubane-1,4-dicarboxylic acid (**(+)-2a**)

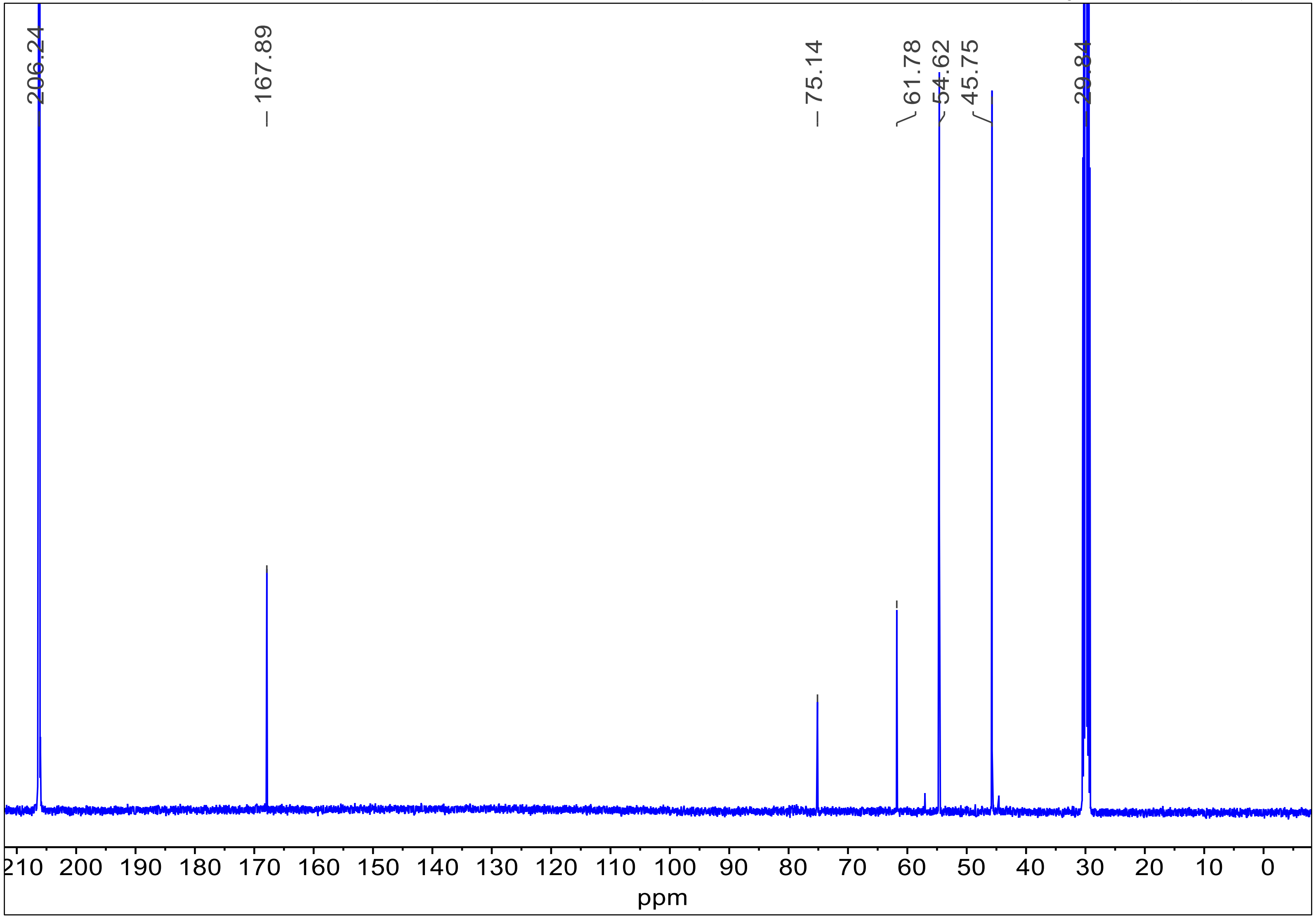

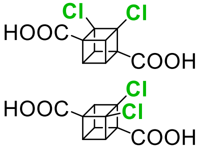

^{13}C APT NMR (100 MHz, acetone-d_6): (1*S*,2*R*,3*R*,4*S*,5*R*,6*R*,7*R*,8*R*)-2,3-dichlorocubane-1,4-dicarboxylic acid (**(-)-2a**) and (1*R*,2*S*,3*S*,4*R*,5*S*,6*S*,7*S*,8*S*)-2,3-dichlorocubane-1,4-dicarboxylic acid (**(+)-2a**)

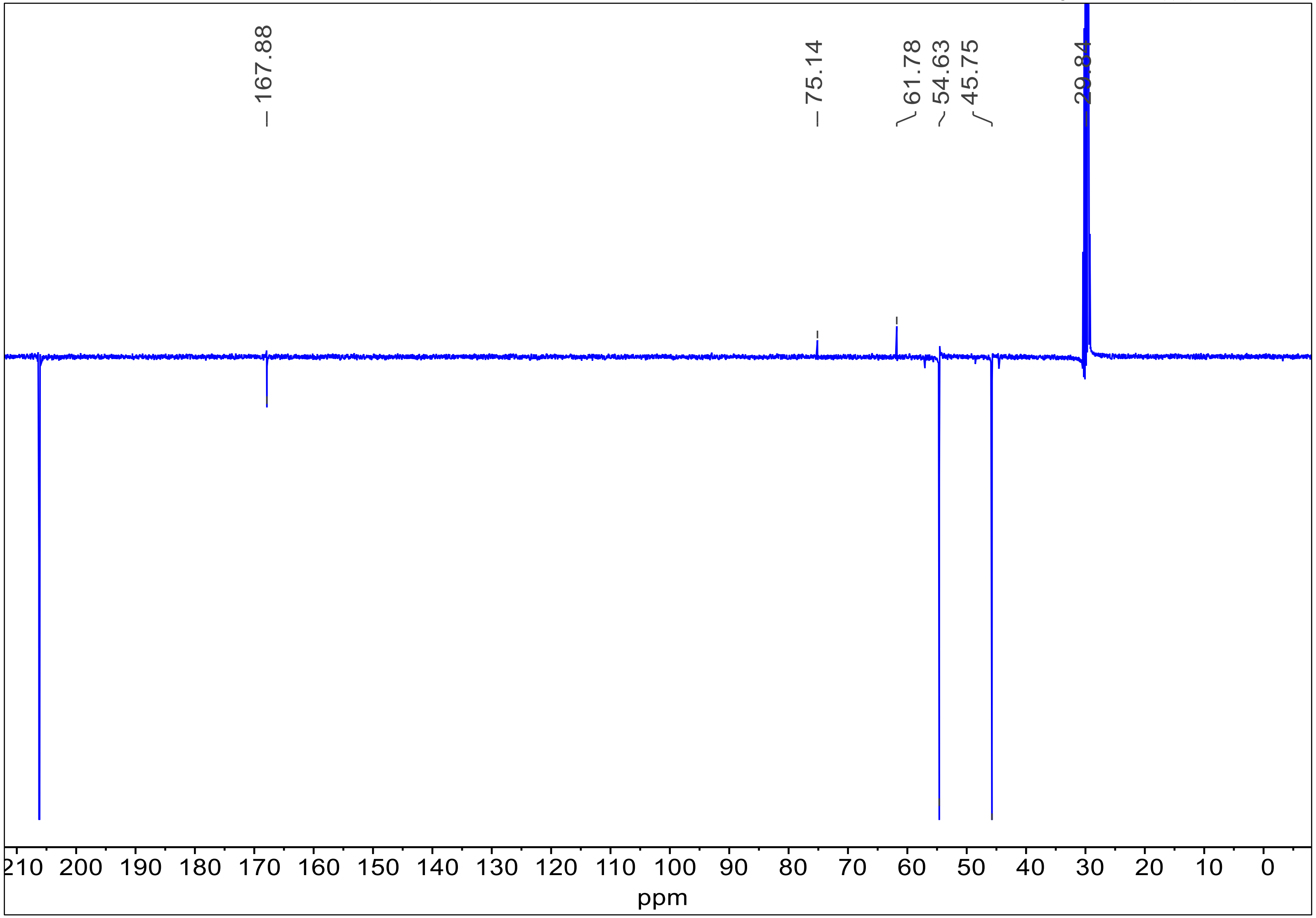

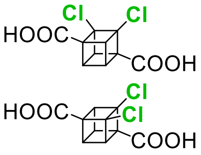

^1H – ^1H COSY (acetone-d_6): (1*S*,2*R*,3*R*,4*S*,5*R*,6*R*,7*R*,8*R*)-2,3-dichlorocubane-1,4-dicarboxylic acid (**(-)-2a**) and (1*R*,2*S*,3*S*,4*R*,5*S*,6*S*,7*S*,8*S*)-2,3-dichlorocubane-1,4-dicarboxylic acid (**(+)-2a**)

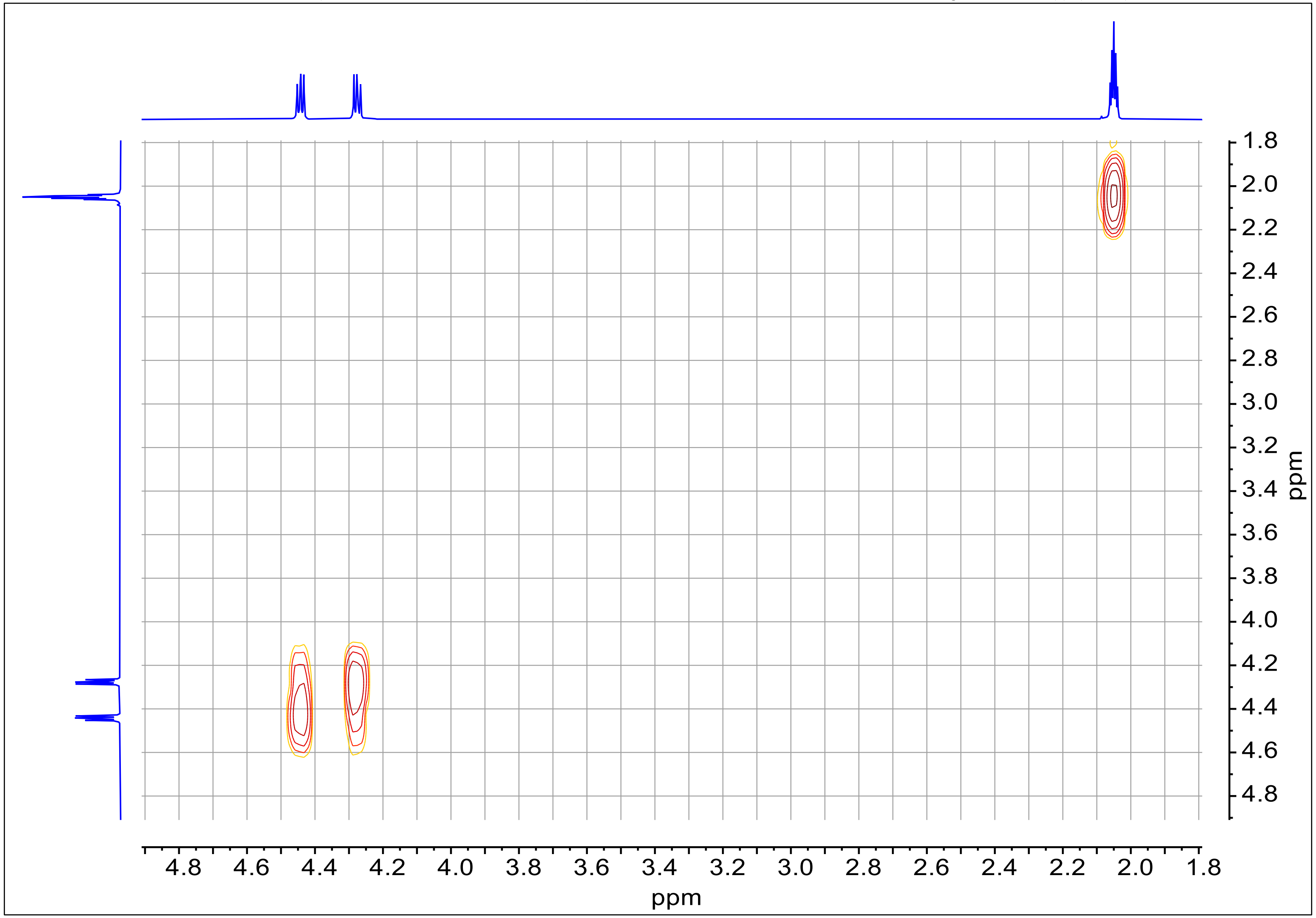

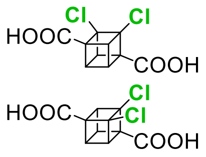

HSQC (acetone-d_6): (1*S*,2*R*,3*R*,4*S*,5*R*,6*R*,7*R*,8*R*)-2,3-dichlorocubane-1,4-dicarboxylic acid (**(-)-2a**) and (1*R*,2*S*,3*S*,4*R*,5*S*,6*S*,7*S*,8*S*)-2,3-dichlorocubane-1,4-dicarboxylic acid (**(+)-2a**)

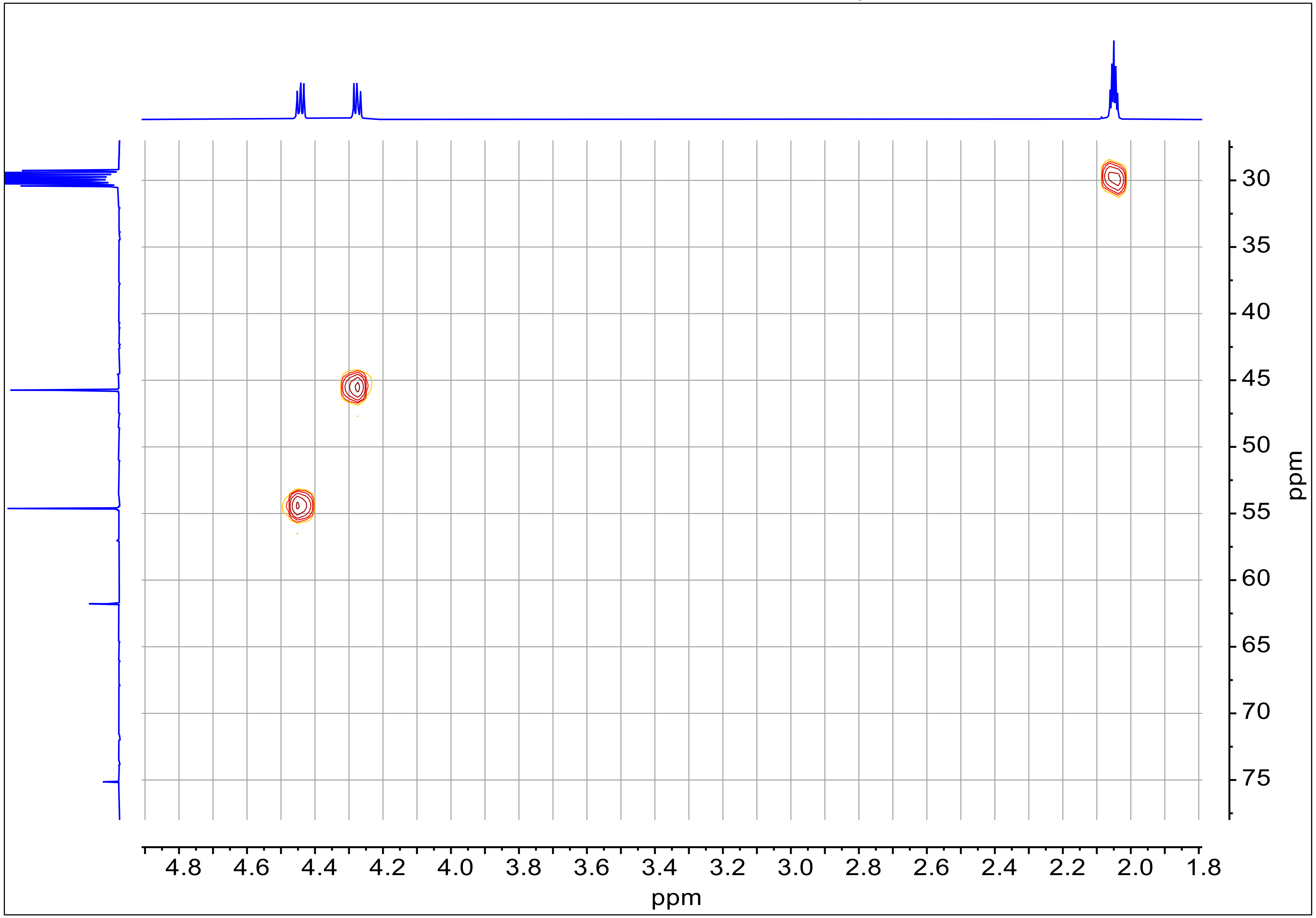

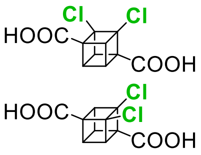

HMBC (acetone-d_6): (1*S*,2*R*,3*R*,4*S*,5*R*,6*R*,7*R*,8*R*)-2,3-dichlorocubane-1,4-dicarboxylic acid (**(-)-2a**) and (1*R*,2*S*,3*S*,4*R*,5*S*,6*S*,7*S*,8*S*)-2,3-dichlorocubane-1,4-dicarboxylic acid (**(+)-2a**)

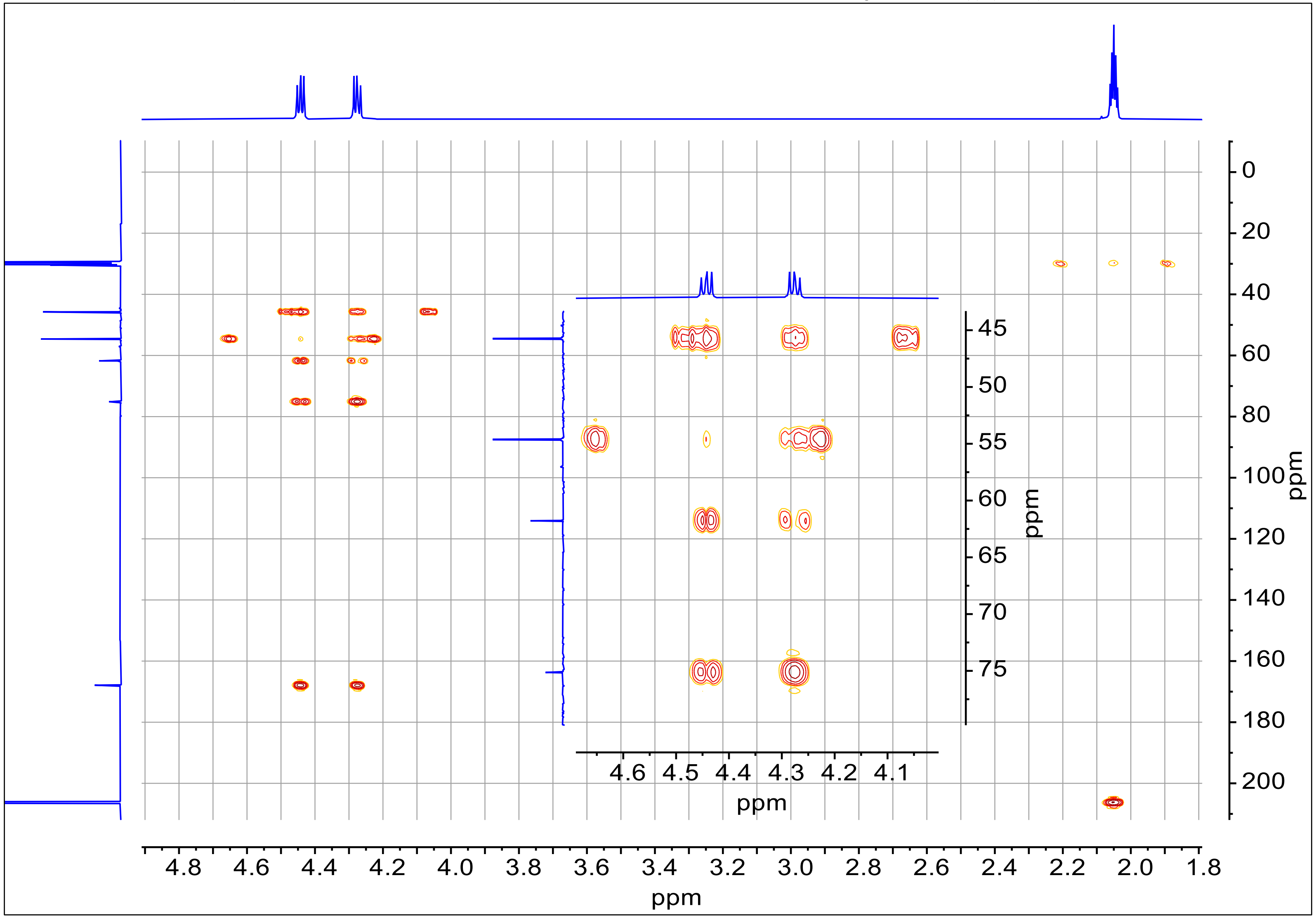

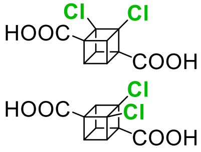

^{1}H NMR (400 MHz, DMSO-d_6): 2,5-Dichlorocubane-1,4-dicarboxylic Acid (**2b**)

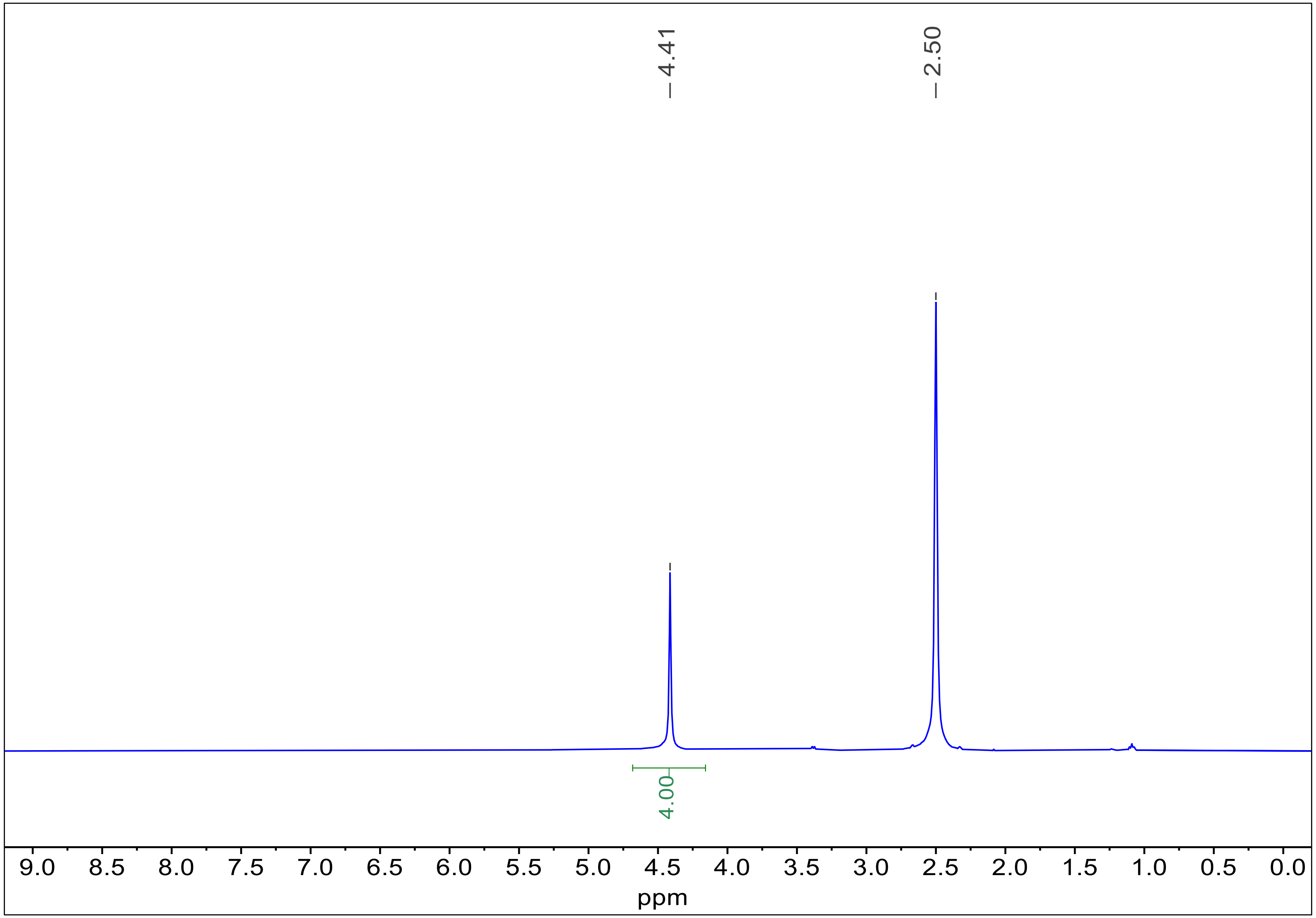

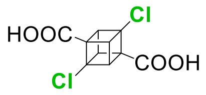

^{13}C {^{1}H} NMR (100 MHz, DMSO-d_6): 2,5-Dichlorocubane-1,4-dicarboxylic Acid (**2b**)

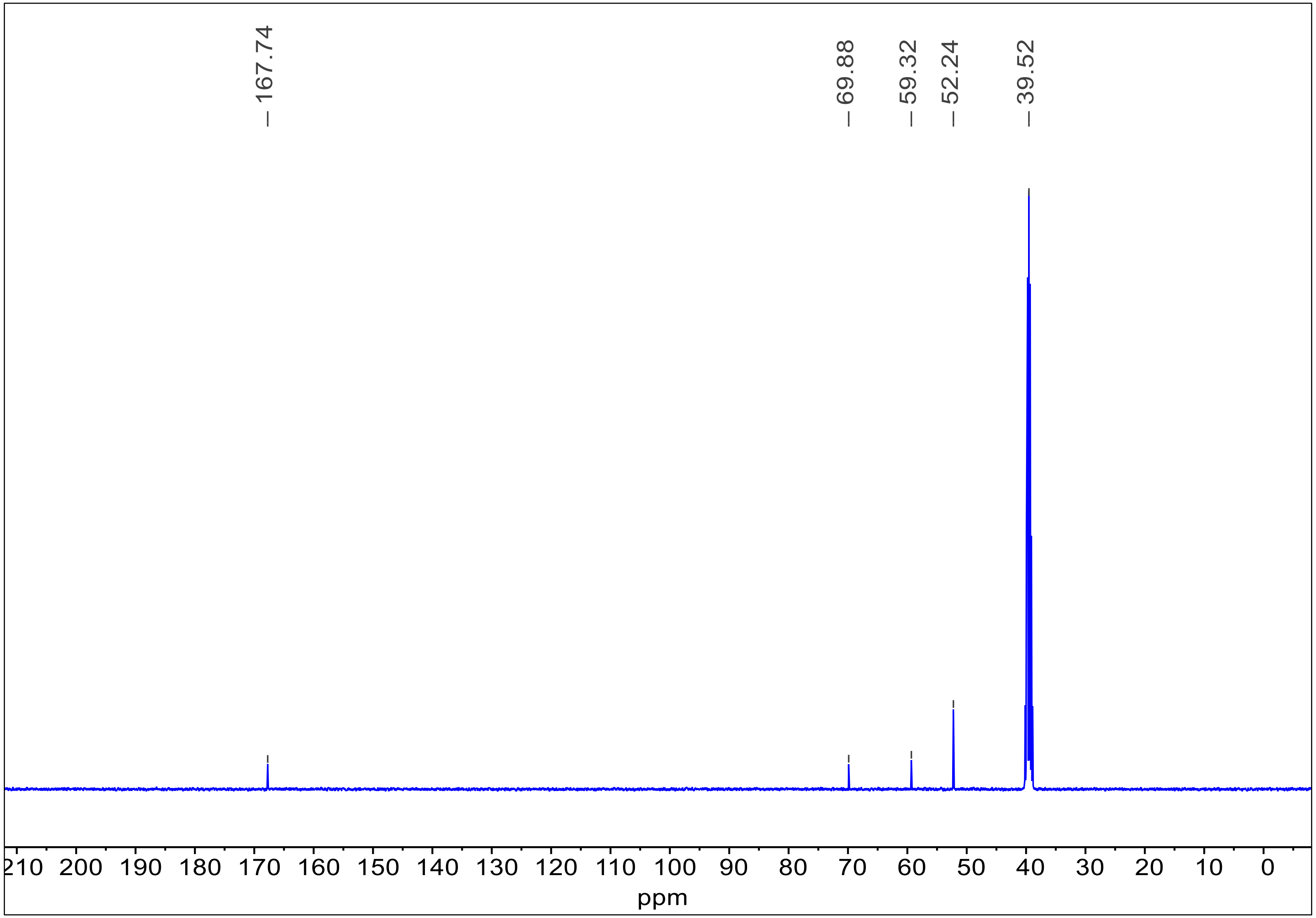

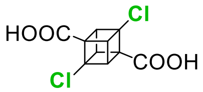

^{13}C APT NMR (100 MHz, DMSO-d_6): 2,5-Dichlorocubane-1,4-dicarboxylic Acid (**2b**)

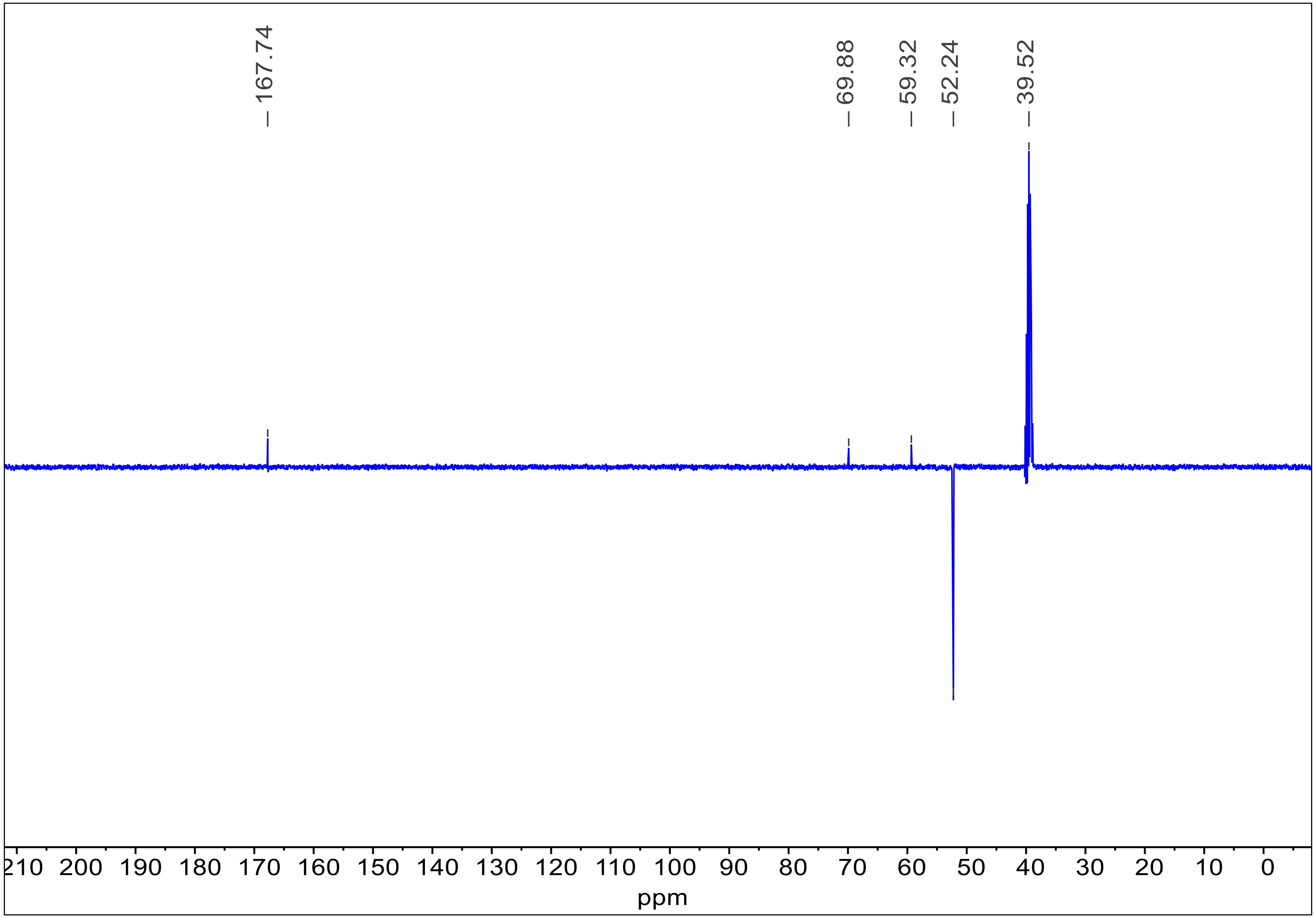

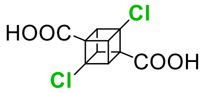

$^1H – ^1H$ COSY (DMSO-d_6): 2,5-Dichlorocubane-1,4-dicarboxylic Acid (**2b**)

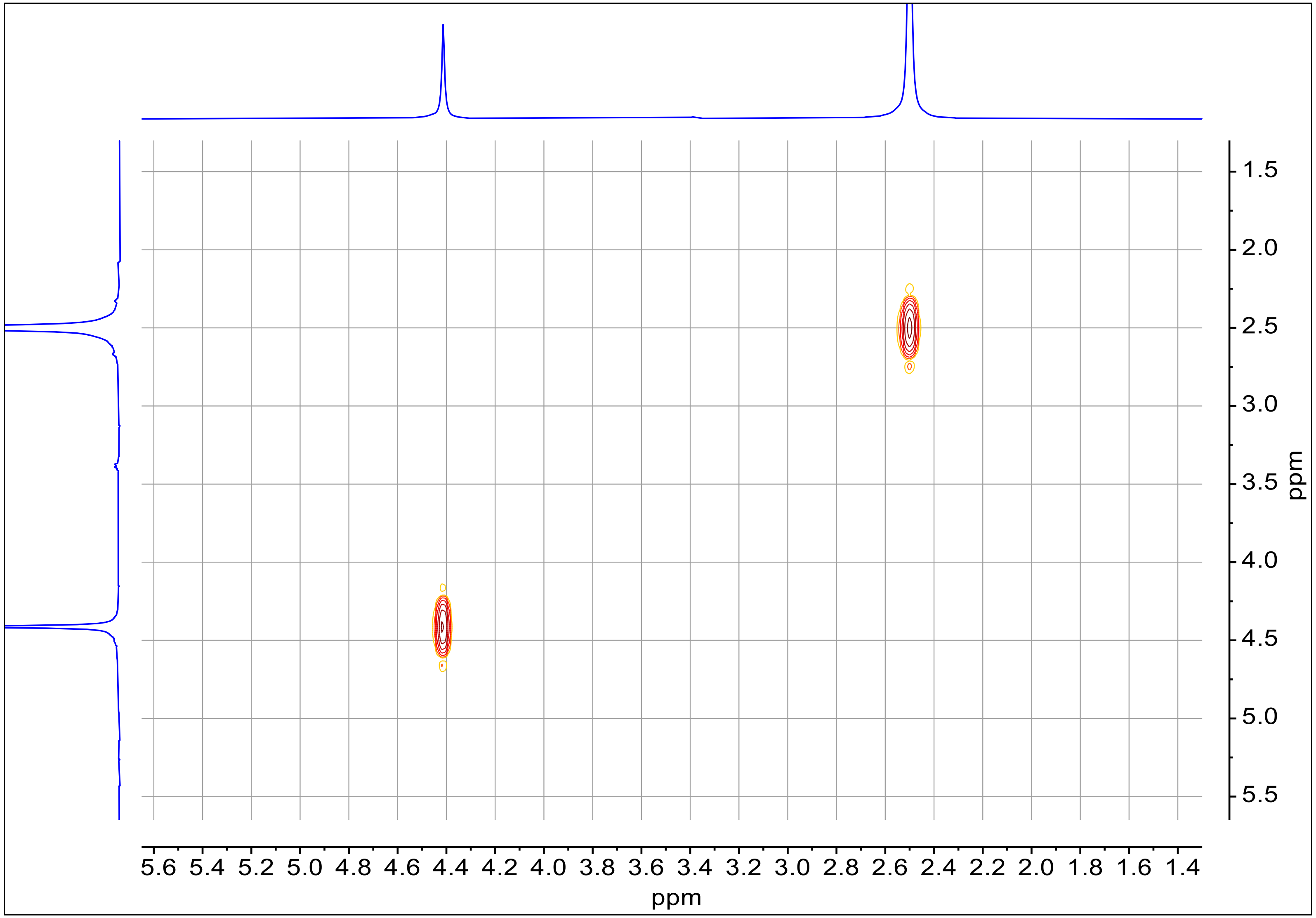

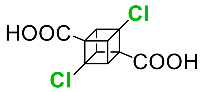

HSQC (DMSO-d_6): 2,5-Dichlorocubane-1,4-dicarboxylic Acid (**2b**)

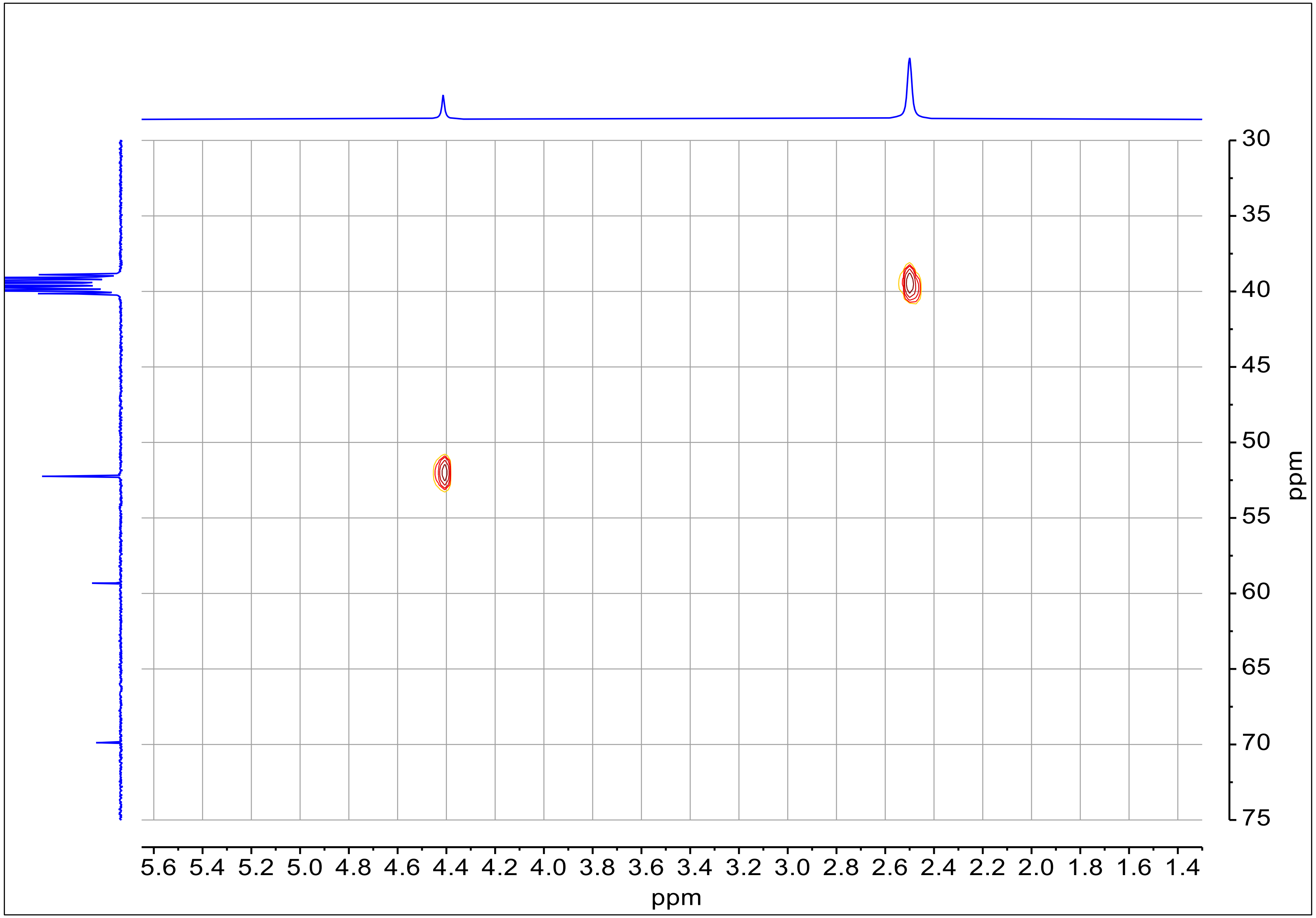

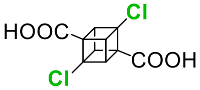

HMBC (DMSO-d_6): 2,5-Dichlorocubane-1,4-dicarboxylic Acid (**2b**)

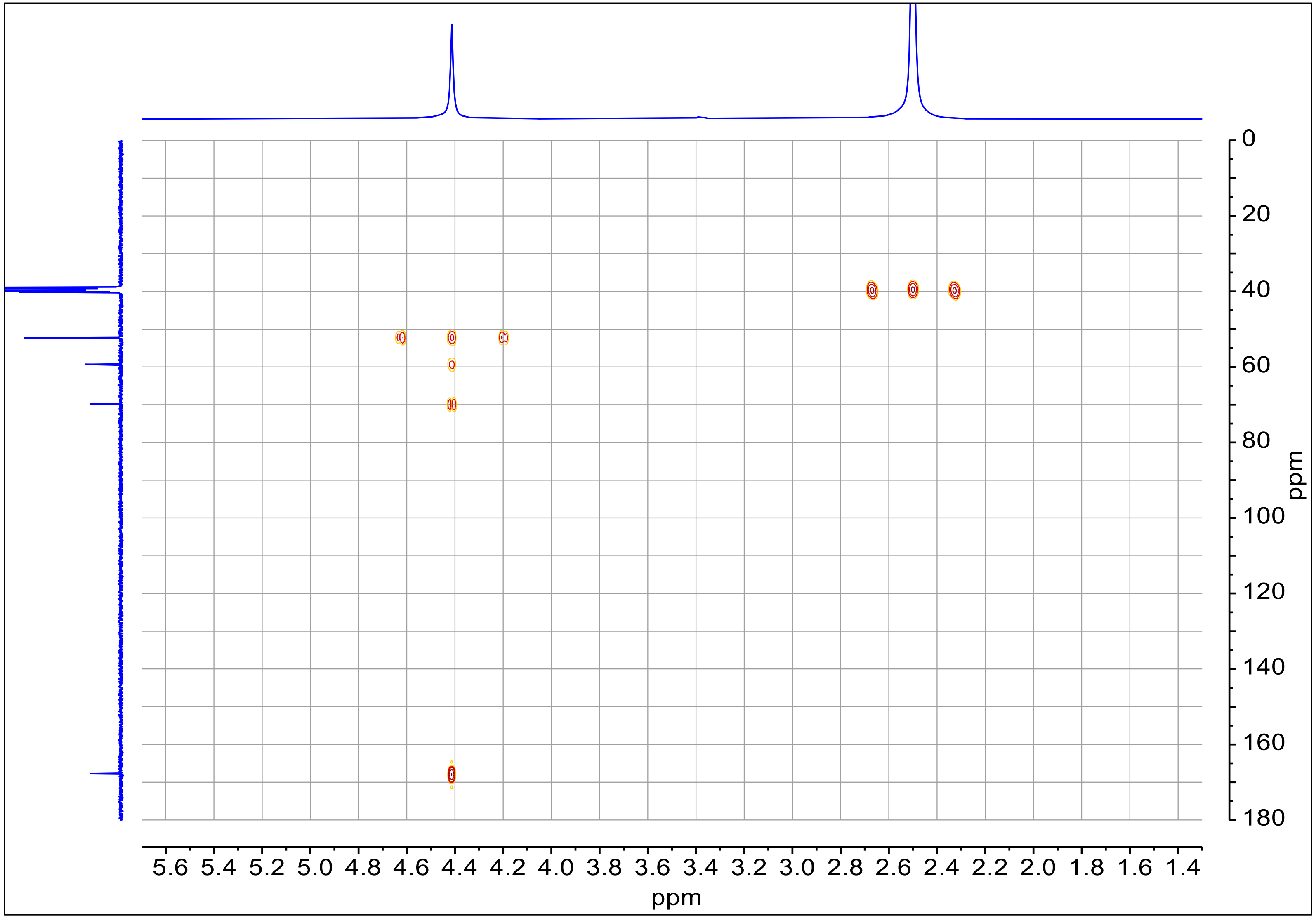

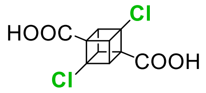

^{1}H NMR (400 MHz, acetone-d_6): 2,6-Dichlorocubane-1,4-dicarboxylic Acid (**2c**)

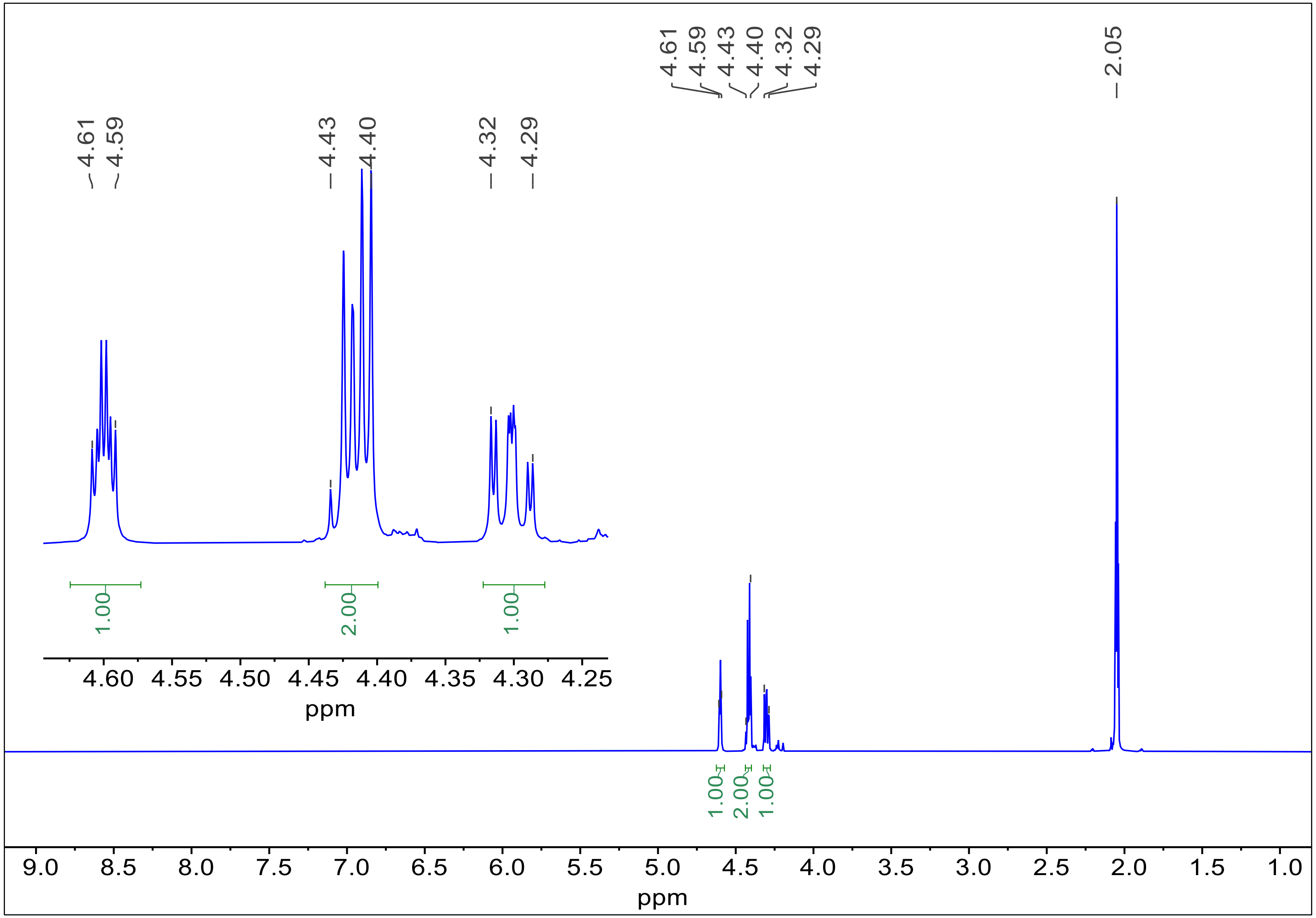

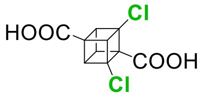

^{13}C {^{1}H} NMR (100 MHz, acetone-d_6): 2,6-Dichlorocubane-1,4-dicarboxylic Acid (**2c**)

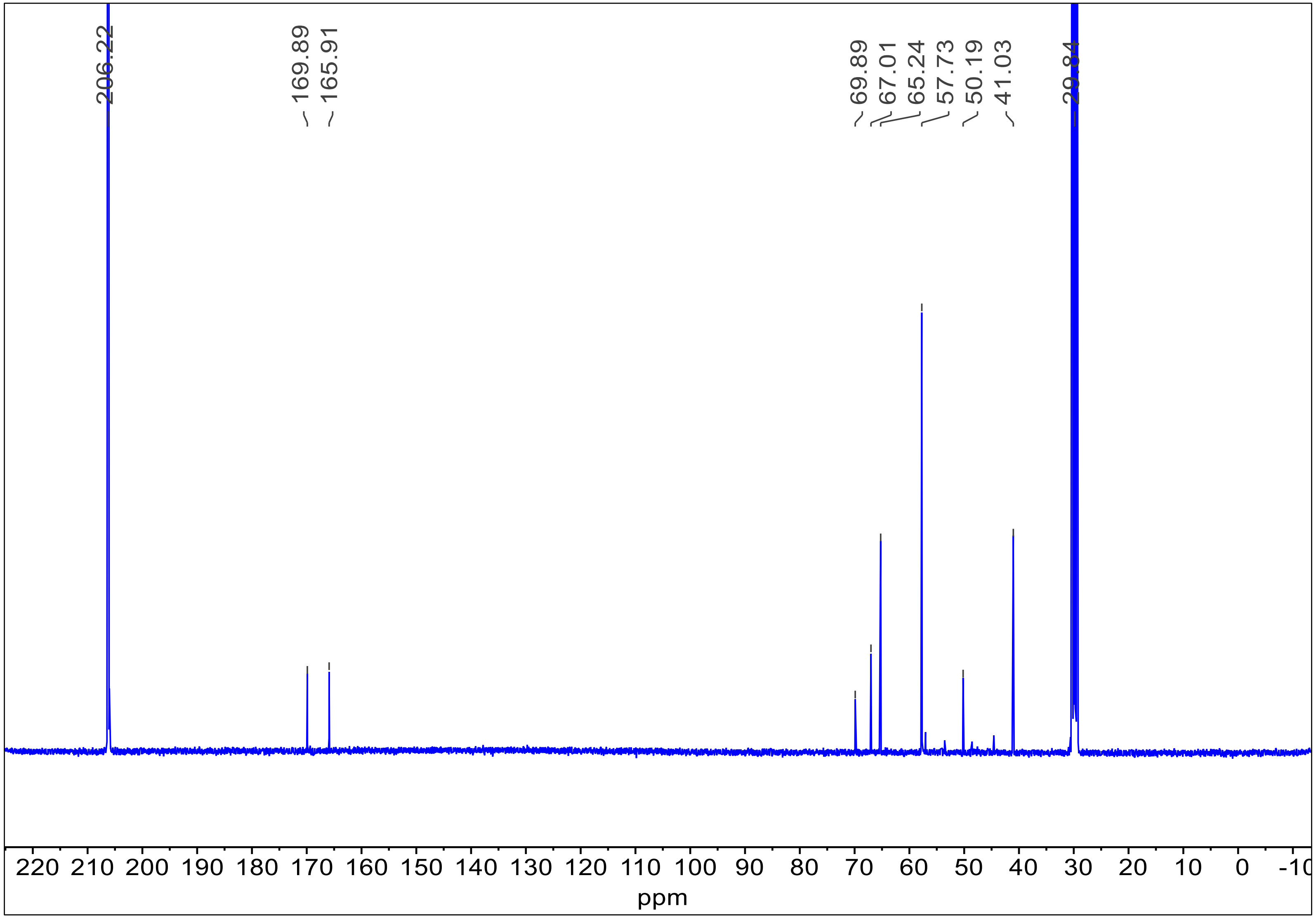

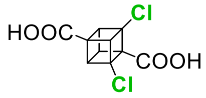

^{13}C APT NMR (100 MHz, acetone-d_6): 2,6-Dichlorocubane-1,4-dicarboxylic Acid (**2c**)

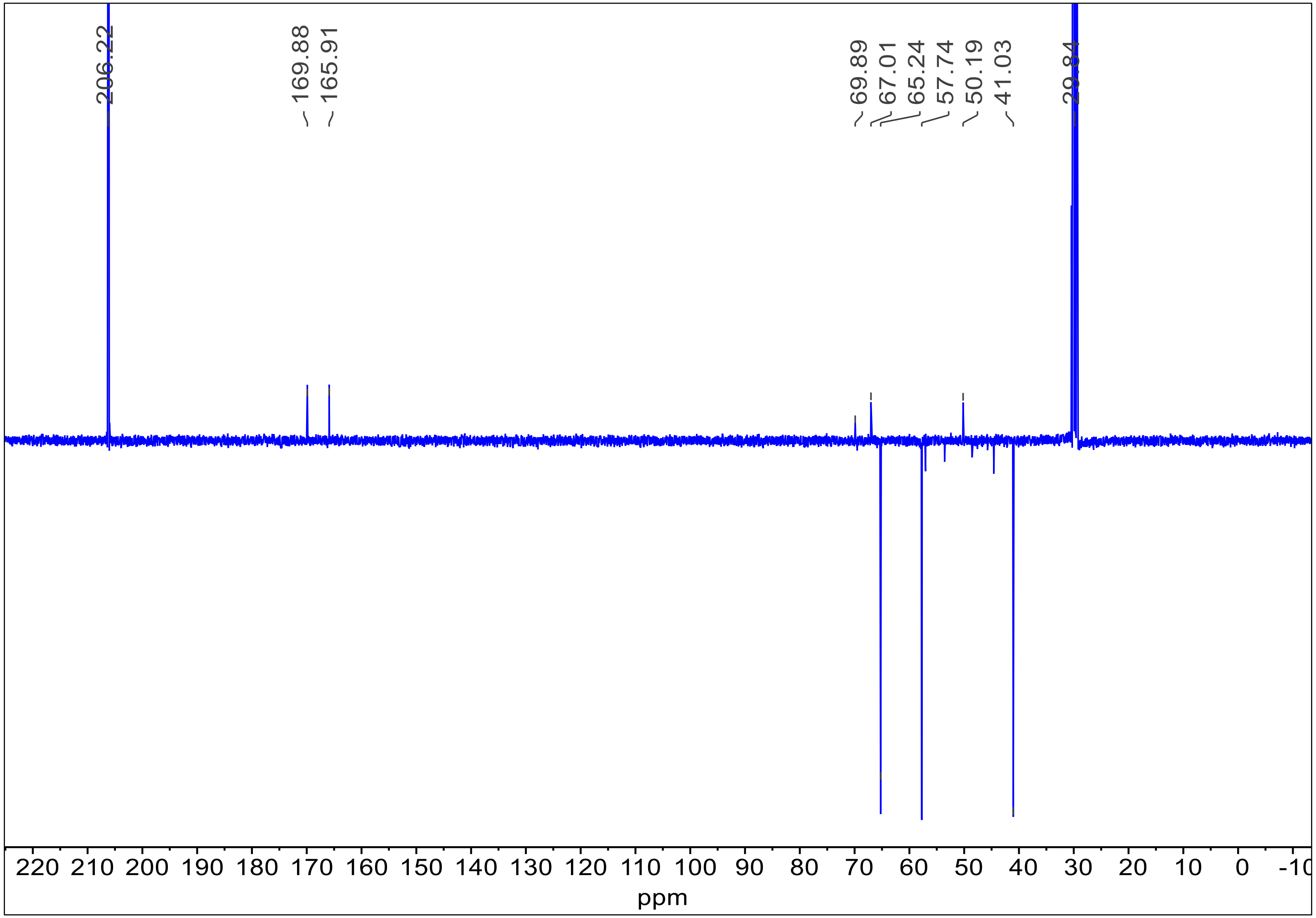

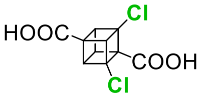

$^1H – ^1H$ COSY (acetone-d_6): 2,6-Dichlorocubane-1,4-dicarboxylic Acid (**2c**)

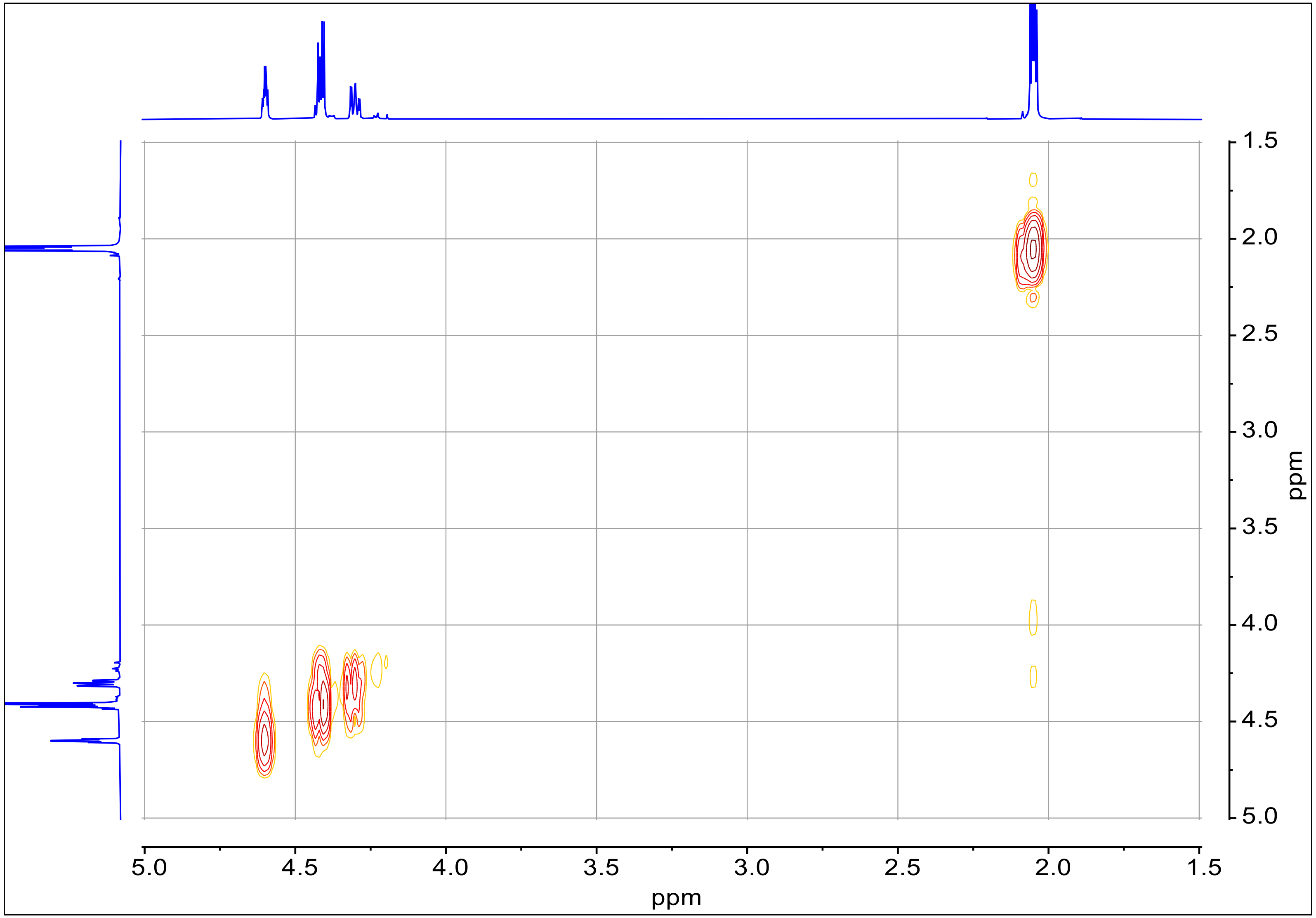

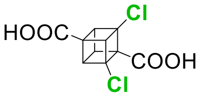

HSQC (acetone-d_6): 2,6-Dichlorocubane-1,4-dicarboxylic Acid (**2c**)

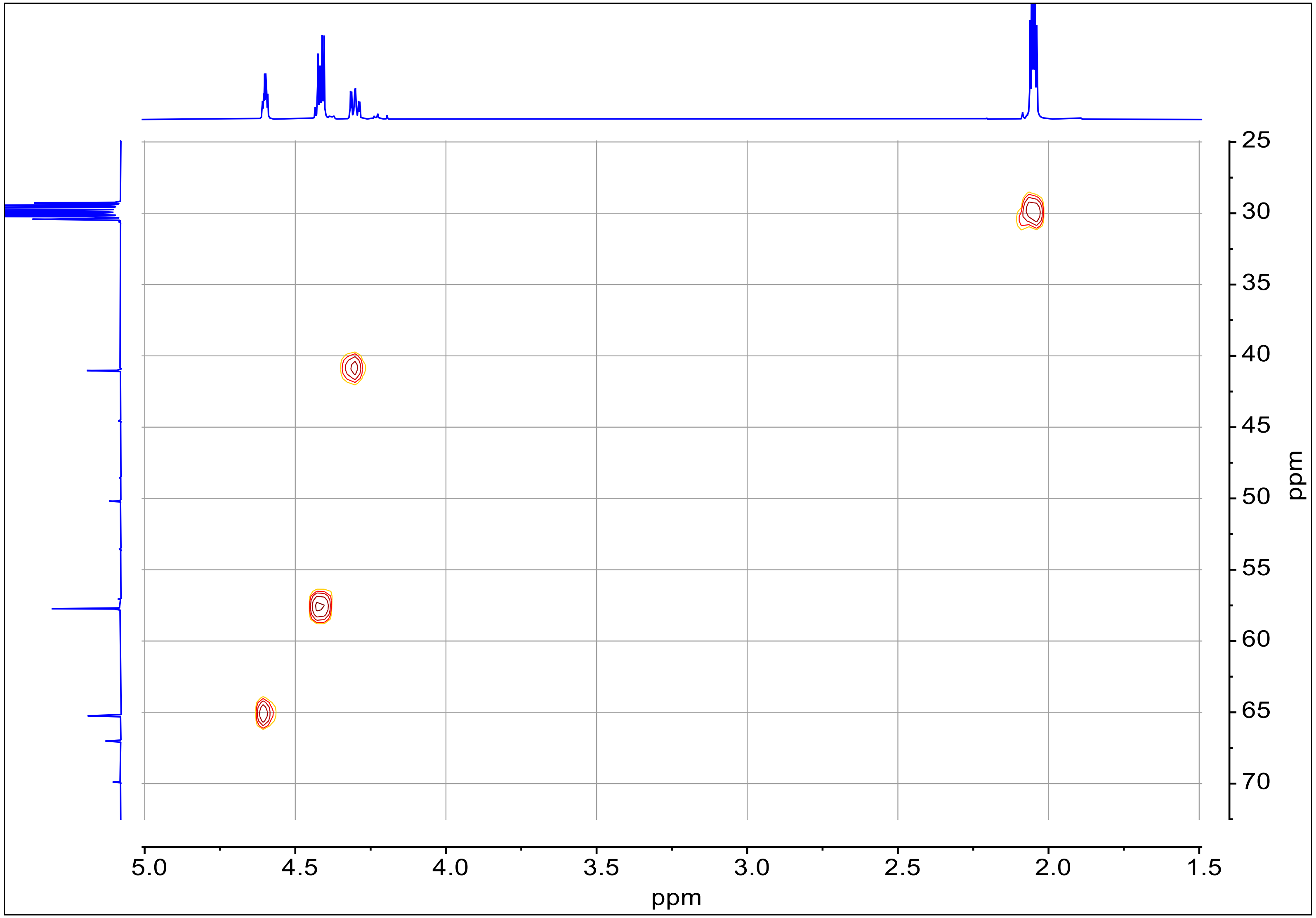

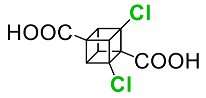

HMBC (acetone-d_6): 2,6-Dichlorocubane-1,4-dicarboxylic Acid (**2c**)

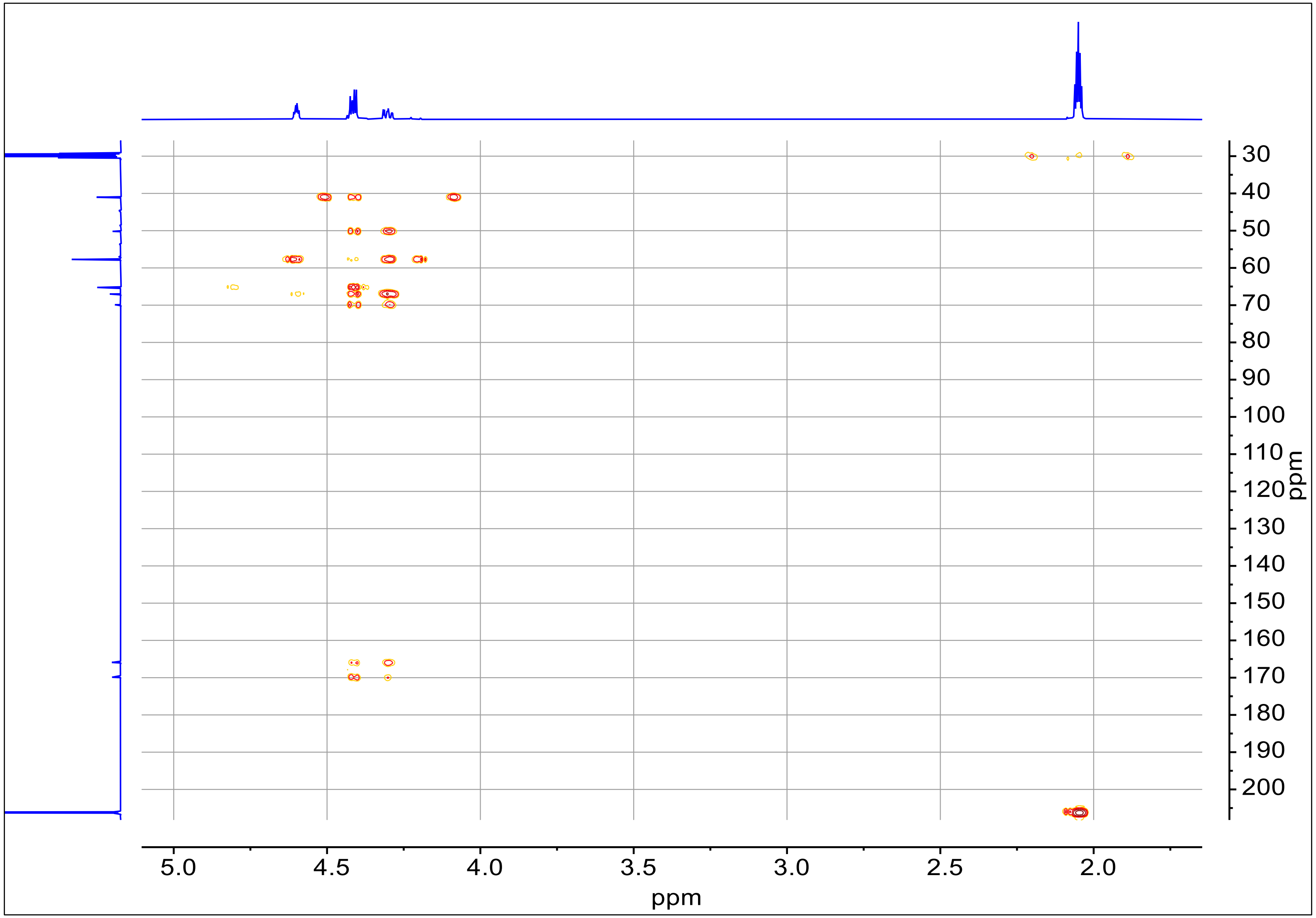

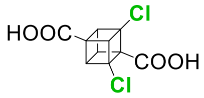

X-ray: Dimethyl 2-Chlorocubane-1,4-dicarboxylate (**1Me**) (displacement ellipsoids are shown at the 30% probability level)

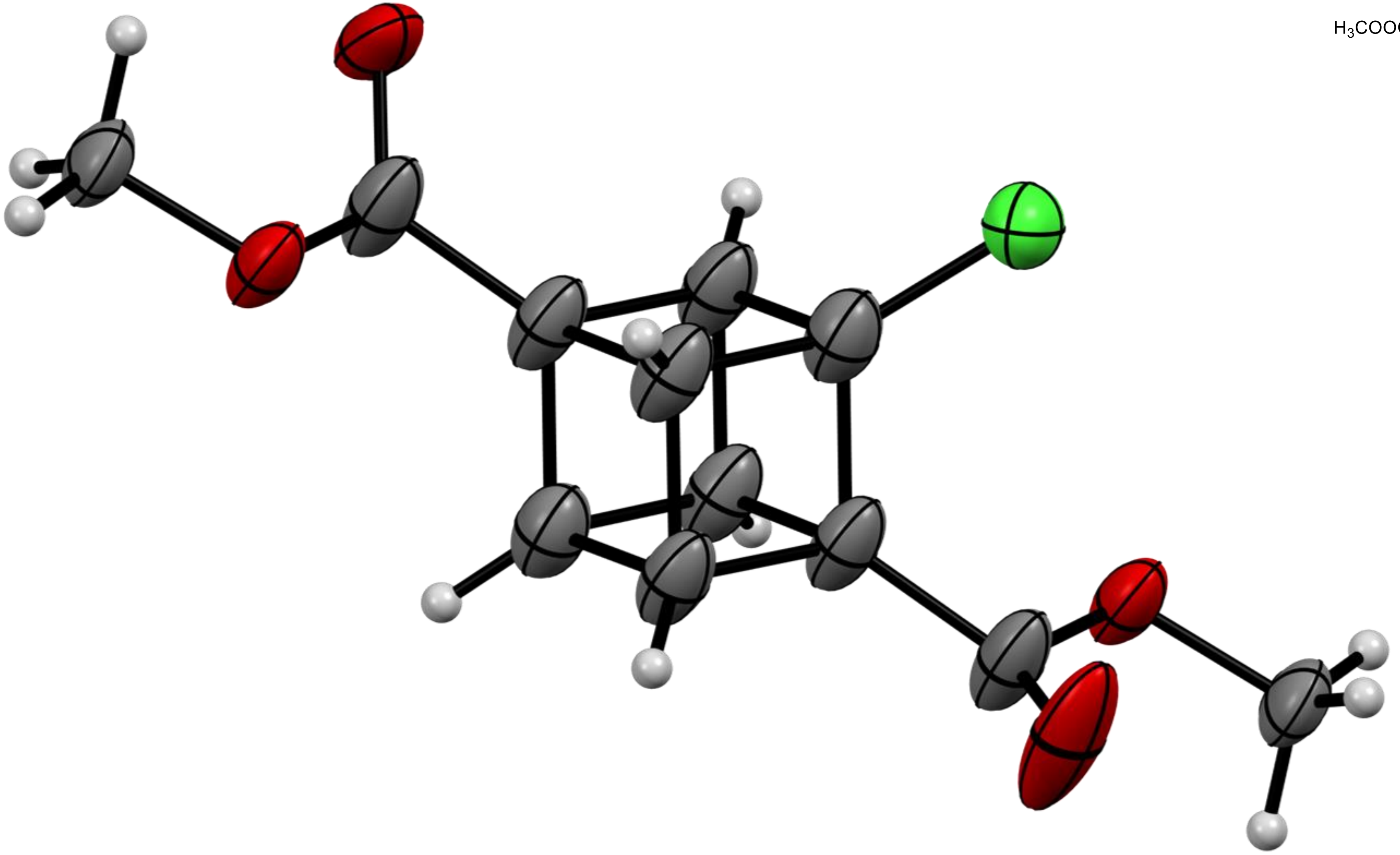

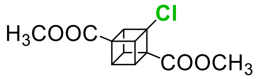

X-ray: Crystal packing of dimethyl 2-chlorocubane-1,4-dicarboxylate (**1Me**) (hydrogen atoms are omitted for clarity)

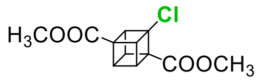

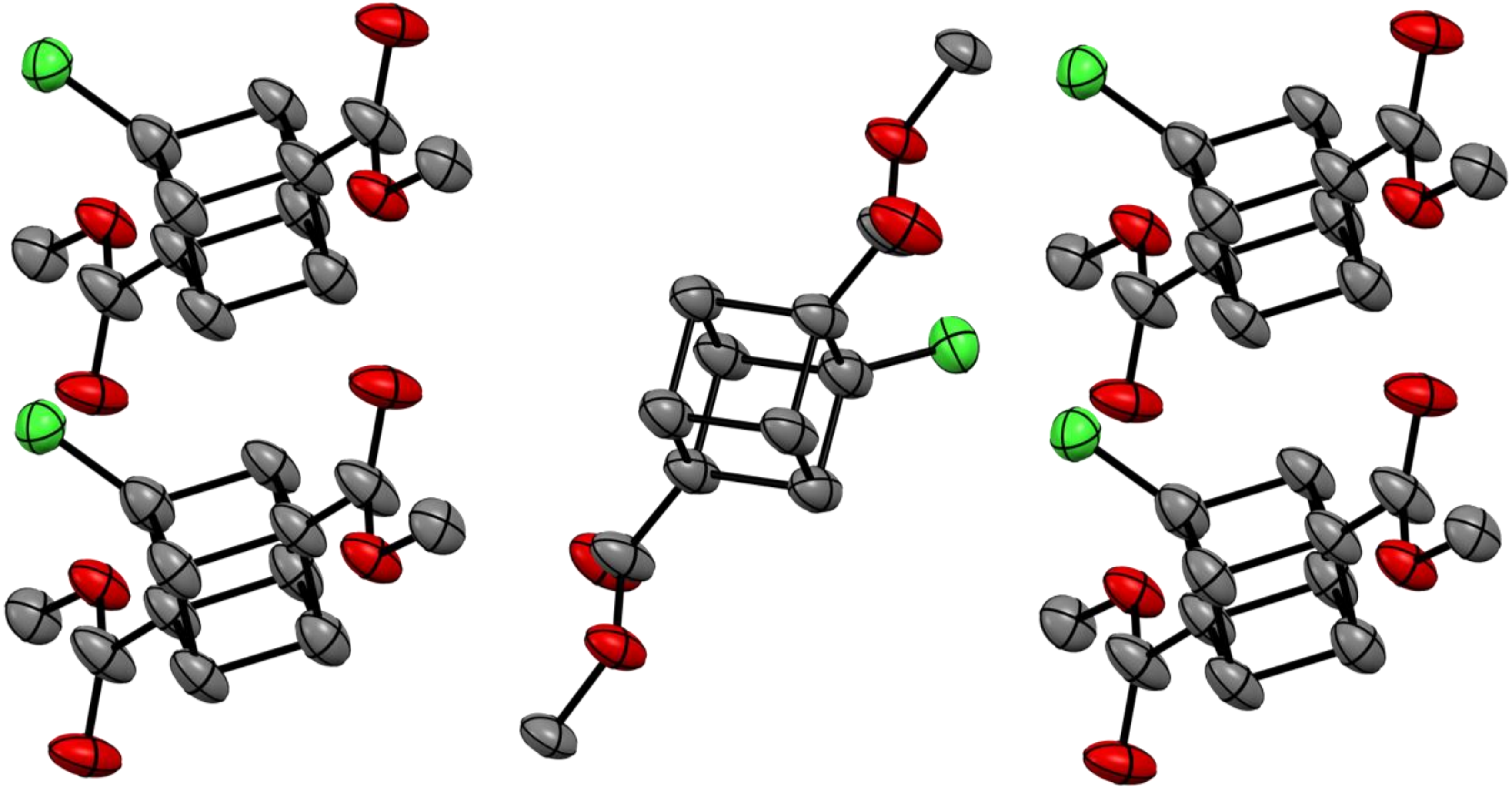

X-ray: Dimethyl (1*S*,2*R*,3*R*,4*S*,5*R*,6*R*,7*R*,8*R*)-2,3-Dichlorocubane-1,4-dicarboxylate (**(-)-2aMe**) and Dimethyl (1*R*,2*S*,3*S*,4*R*,5*S*,6*S*,7*S*,8*S*)-2,3-Dichlorocubane-1,4-dicarboxylate (**(+)-2aMe**) (displacement ellipsoids are shown at the 30% probability level)

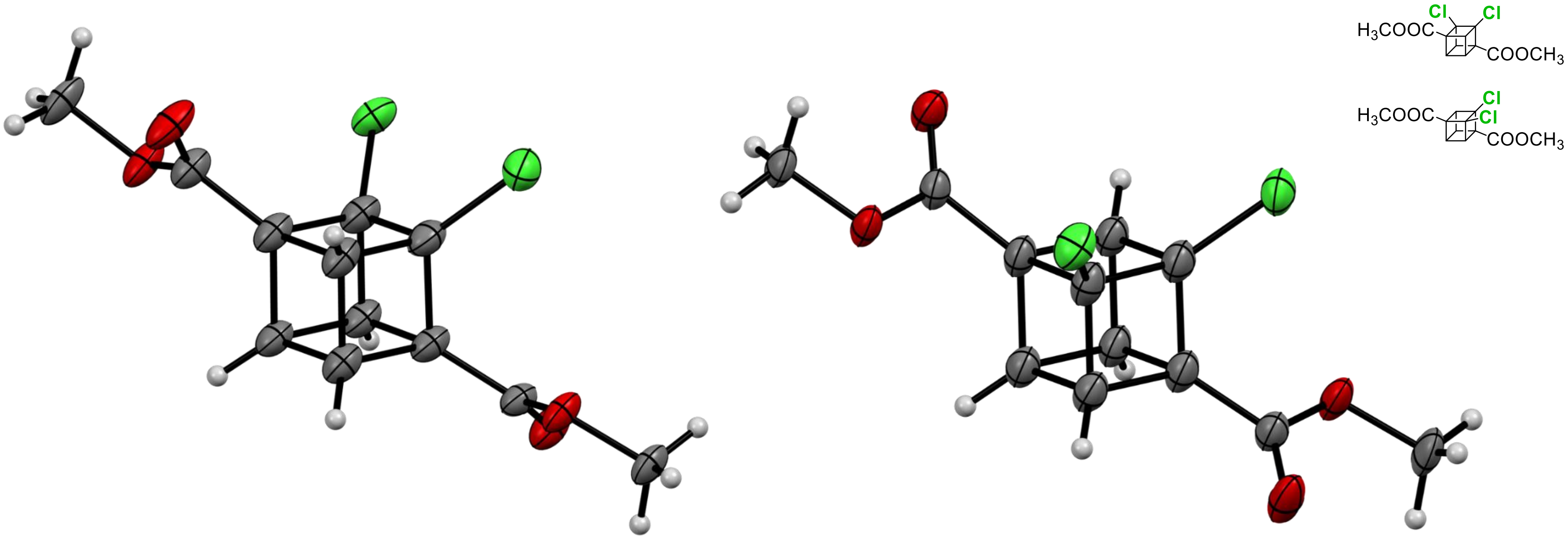

X-ray: Crystal packing of dimethyl (1*S*,2*R*,3*R*,4*S*,5*R*,6*R*,7*R*,8*R*)-2,3-dichlorocubane-1,4-dicarboxylate (**(-)-2aMe**) and dimethyl (1*R*,2*S*,3*S*,4*R*,5*S*,6*S*,7*S*,8*S*)-2,3-dichlorocubane-1,4-dicarboxylate (**(+)-2aMe**) (hydrogen atoms are omitted for clarity)

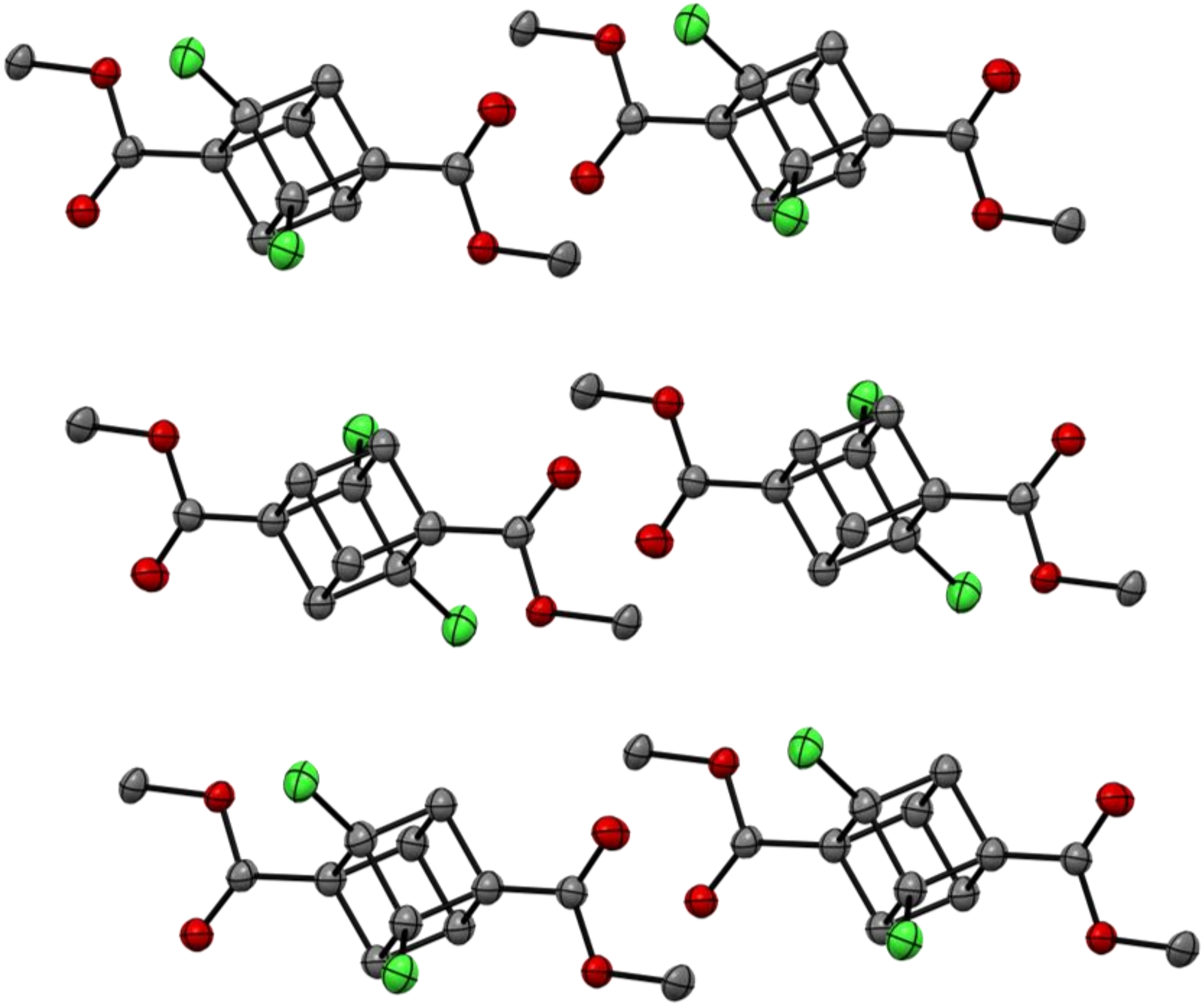

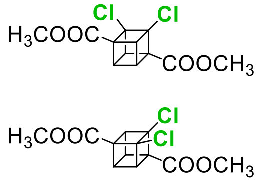

X-ray: Dimethyl 2,7-Dichlorocubane-1,4-dicarboxylate (**2bMe**) (displacement ellipsoids are shown at the 30% probability level)

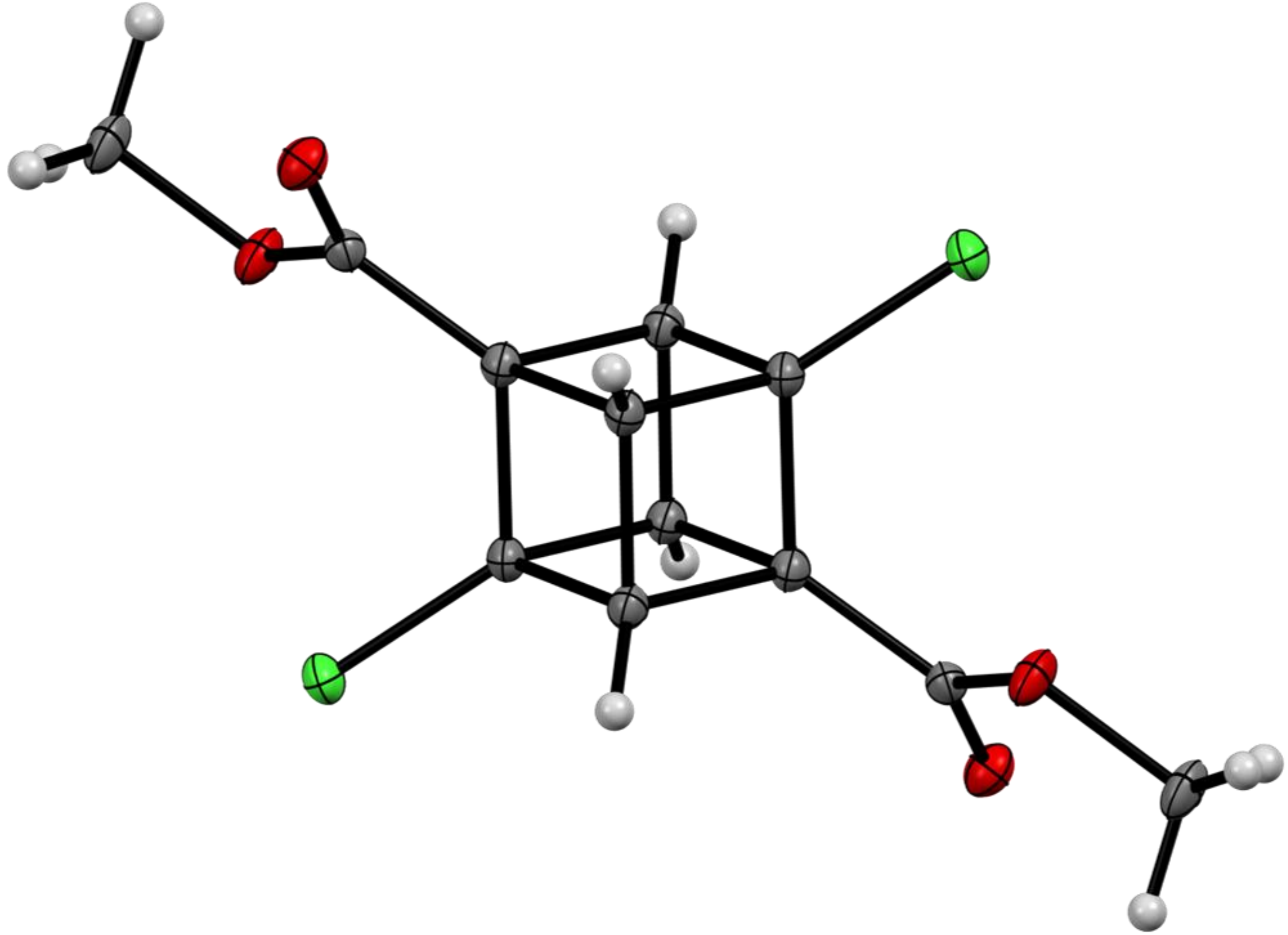

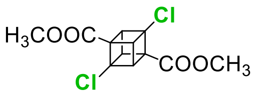

X-ray: Crystal packing of dimethyl 2,7-dichlorocubane-1,4-dicarboxylate (**2bMe**) (hydrogen atoms are omitted for clarity)

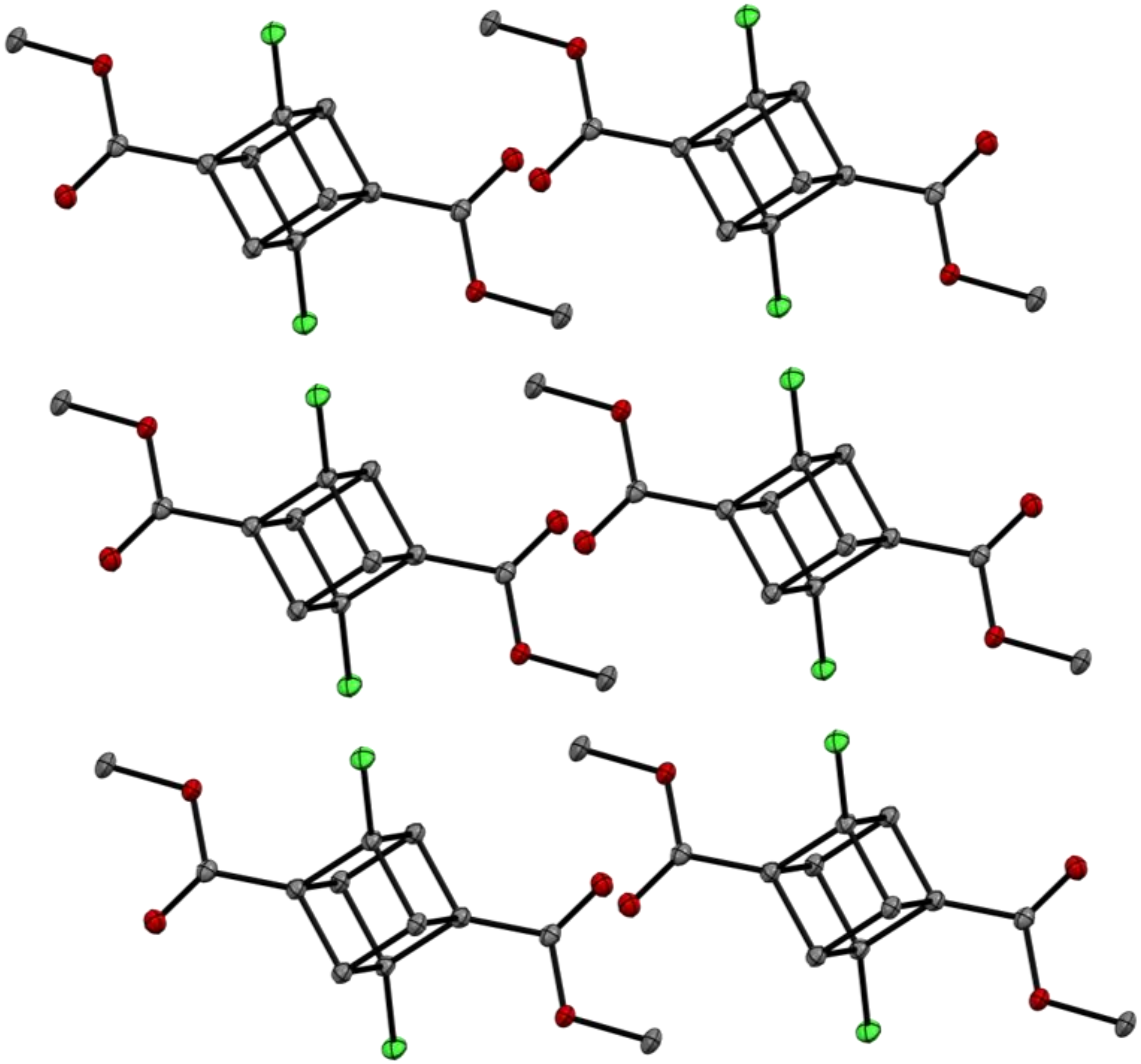

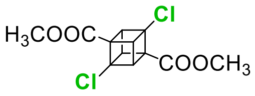

X-ray: Dimethyl 2,6-Dichlorocubane-1,4-dicarboxylate (**2cMe**) (displacement ellipsoids are shown at the 30% probability level)

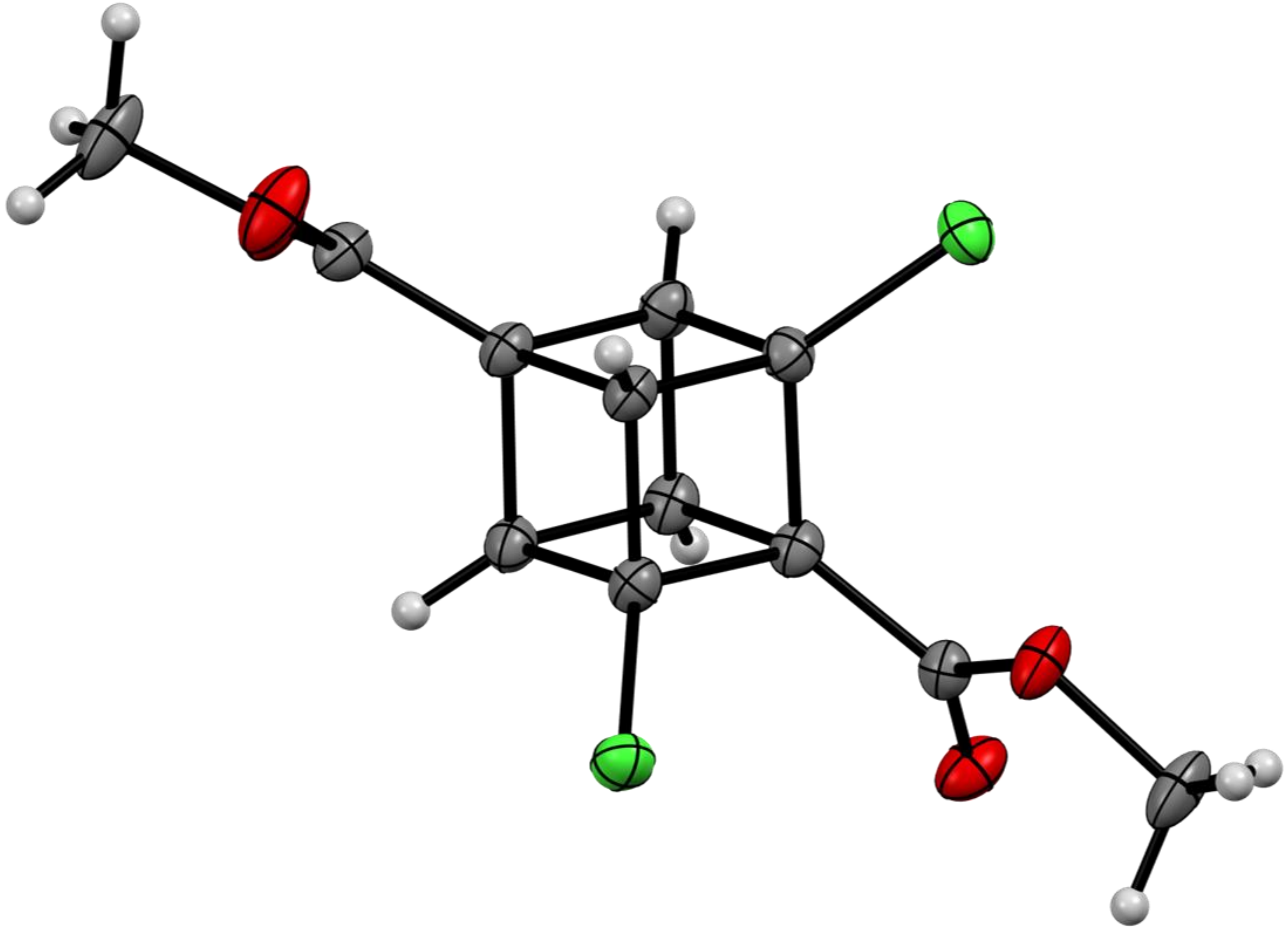

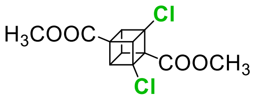

X-ray: Crystal packing of dimethyl 2,6-dichlorocubane-1,4-dicarboxylate (**2cMe**) (hydrogen atoms are omitted for clarity)

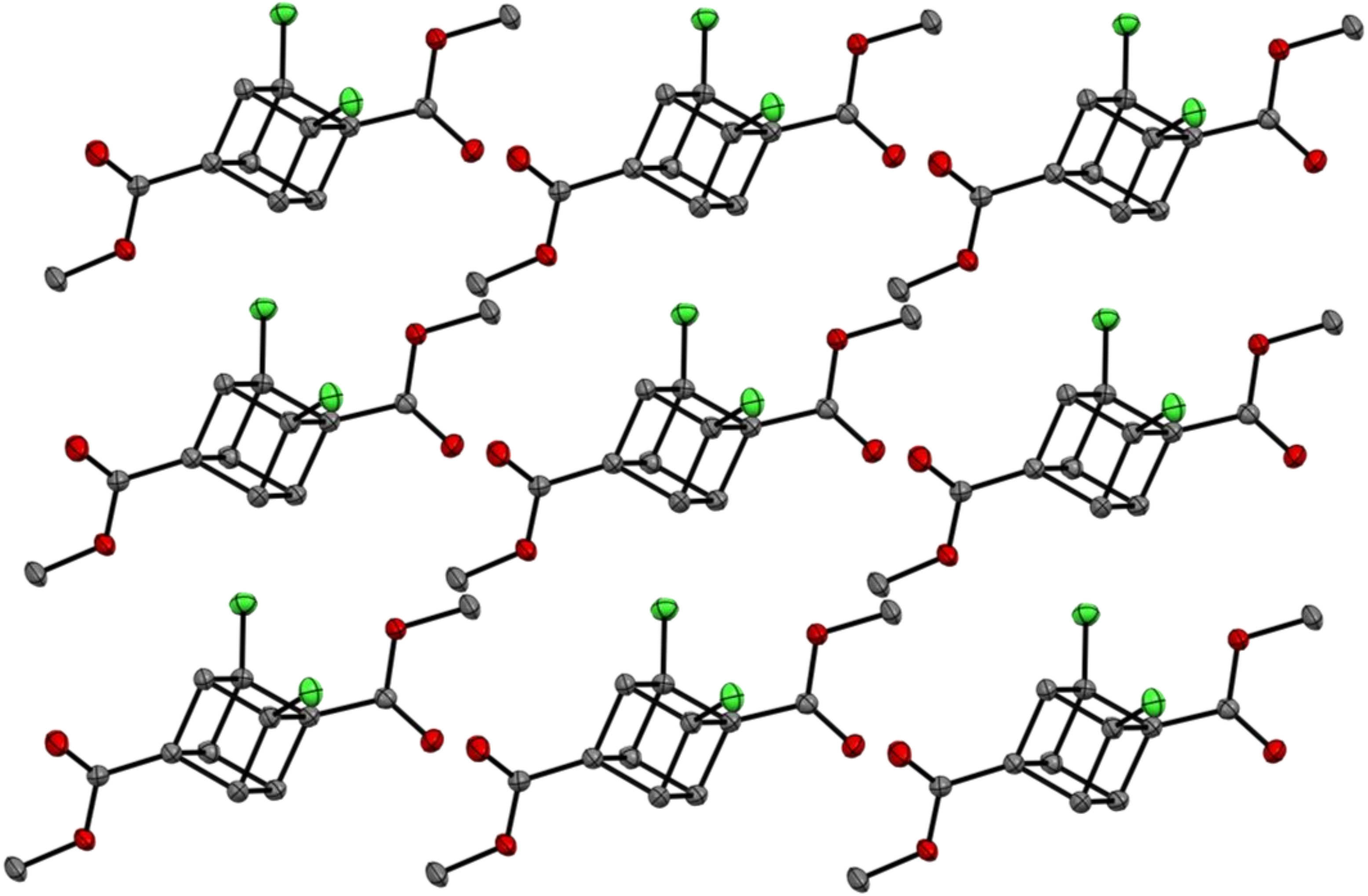

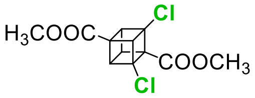

Supplement: Supplementary file 1 — jo2c02872_si_001.pdf [file jo2c02872_si_001.pdf]
